# QTL Mapping and Heterosis Analysis for Fiber Quality Traits Across Multiple Genetic Populations and Environments in Upland Cotton

**DOI:** 10.3389/fpls.2018.01364

**Published:** 2018-10-15

**Authors:** Cong Li, Hurong Yu, Cheng Li, Tianlun Zhao, Yating Dong, Xiaolei Deng, Jiahui Hu, Yi Zhang, Fan Zhang, M. K. Daud, Jinhong Chen, Shuijin Zhu

**Affiliations:** ^1^Department of Agronomy, Zhejiang University, Hangzhou, China; ^2^Department of Biotechnology and Genetic Engineering, Kohat University of Science and Technology, Kohat, Pakistan

**Keywords:** upland cotton (*Gossypium hirsutum* L.), heterosis, fiber quality, multiple genetic populations, quantitative trait loci mapping

## Abstract

An “immortalized F_2_” (IF_2_) population and two reciprocal backcross (HSBCF_1_ and MARBCF_1_) populations were constructed to investigate the genetic bases of fiber quality traits in upland cotton across four different environments. A relatively high level of heterosis for micronaire (MIC) in IF_2_ population as well as fiber length (FL) and MIC in MARBCF_1_ population was observed. A total of 167 quantitative trait loci (QTLs) were detected in the three related experimental populations and their corresponding midparental heterosis (MPH) datasets using the composite interval mapping (CIM) approach. An analysis of genetic effects of QTLs detected in different populations and their MPH datasets showed 16 (24.24%) QTLs of partial dominance, and 46 (69.70%) QTLs of overdominance were identified in an IF_2_ population; 89 (62.68%) additive QTLs, three (2.11%) partial dominant QTLs, and 49 (34.51%) over-dominant QTLs were detected in two BCF_1_ populations. Multi-environment analysis showed 48 and 56 main-QTLs (m-QTLs) and 132 and 182 epistasis-QTLs (e-QTLs), by inclusive composite interval mapping (ICIM) in IF_2_ and two BCF_1_ populations, respectively. Phenotypic variance explained by e-QTLs, except for MARBCF_1_ population, was higher than that by m-QTLs. Thus, the overdominant, partial dominant, and epistasis effects were the main causes of heterosis in the IF_2_ population, whereas the additive, overdominant, and epistasis effects were the primary genetic basis of heterosis in the two BCF_1_ populations. Altogether, additive effect, partial dominance, overdominance, and epistasis contributed to fiber quality heterosis in upland cotton, but overdominance and epistasis were the most important factors.

## Introduction

Cotton (*Gossypium* sp.) is the most widely cultivated natural fiber source worldwide. There are two cultivated tetraploid cotton species, *Gossypium hirsutum* L. and *G. barbadense* L. The most important cotton species, upland cotton (*G. hirsutum* L.), accounts for ~95% of the overall cotton production (Sun et al., 2012). Fiber quality is the most important factor in the textile industry. Poor fiber quality is the greatest concern for the major cotton producing countries, including China, and its improvement has been a priority for cotton breeders. Fiber quality traits are quantitative in nature and possess complex genetic mechanisms. They show different genetic characteristics in different populations and are easily influenced by varied environments (Shen et al., 2005; Sun et al., 2012; Zhang et al., 2012; Tan et al., 2014). Furthermore, significant heterosis exists in fiber quality traits of upland cotton (Meredith and Bridge, 1972; Tang et al., 1993). Marker-assisted selection (MAS) is one of the most effective methods to improve such complex traits and has been greatly exploited by cotton breeders over the past three decades. To date, more than 1,000 quantitative trait loci (QTLs) related to fiber quality traits have been published (Shen et al., 2005; Wu et al., 2008; Sun et al., 2012; Zhang et al., 2012; Said et al., 2013, 2015; Tan et al., 2014; Wang et al., 2016). Most of these QTLs have been detected based on interspecific segregating populations between *G. hirsutum* L. and *G. barbadense* L. However, the use of these fiber quality QTLs in upland cotton breeding has some limitations. For example, some significant QTLs (alleles) from *G. barbadense* L. often cannot be found in *G. hirsutum* L., and markers with polymorphisms between the two species are likely to be monomorphic in *G. hirsutum* L. (Lacape et al., 2005; Yu et al., 2013). On the other hand, owing to the low level of intraspecific polymorphism among the upland cotton cultivars, genetic maps that use simple sequence repeat (SSR) markers in upland cotton intraspecific populations present low density (Zhang et al., 2012; Tan et al., 2014). Recently, based on intraspecific populations, QTL detection using single nucleotide polymorphism (SNP) markers, with their abundant genetic variation, has been developed as a superior strategy (Li et al., 2016; Zhang et al., 2016).

Hybrids show more vigor and adaptivity than their parents, a common phenomenon known as heterosis (Shull, 1914). Heterosis contributes greatly to the production of hybrid cultivars; however, there is limited understanding about its genetic basis. Early explanations focused on the fact that two parents frequently donate different alleles at any given locus in the hybrid. It has been proposed that dominance and/or overdominance provides the heterozygote an advantage by operating between these alleles (Richey, 1942; Stuber et al., 1992; Li et al., 2001; Birchler et al., 2010), while a competing hypothesis states that epistasis is the main contributor of heterosiss (Hull, 1945; Crow, 1948; Williams, 1959; Minvielle, 1987; Yu et al., 1997; Li et al., 2001). There have been numerous studies on the genetic analyses of different crops, favoring one or the other of these hypotheses (Richey, 1942; Hull, 1945; Crow, 1948; Williams, 1959; Minvielle, 1987; Stuber et al., 1992; Li et al., 2001; Birchler et al., 2010).

Genetic analysis of hybrid cultivars based on genetic maps is an effective strategy. It is necessary to have a suitable experimental design to examine the molecular basis of heterosis. In 1952, Comstock and Robinson devised the North Carolina design III (Design III) mating scheme, which was the original use of backcross designs to analyze heterosis. Stuber et al. (1992) studied maize heterosis based on a modified Design III produced from F_3_ families and concluded that overdominance was the main cause of grain yield heterosis. Other maize researchers also reached a similar conclusion (Lu et al., 2003; Lariepe et al., 2012). Xiao et al. (1995) explored heterosis in two rice BC_1_F_7_ populations and observed that dominance was the major cause. By reanalyzing the observed phenotype values of maize (Stuber et al., 1992) and rice (Xiao et al., 1995) using the method of multiple-interval mapping (MIM), Garcia et al. (2008) indicated that the dominant effect was the main contributor to the heterosis of maize, whereas in rice, additive × additive epistatic interactions could be the major cause. Luo et al. (2009) explored the basis of heterosis in rice based on recombinant inbred lines (RILs) and two BCF_1_ populations and found that additive and overdominant effects of epistatic loci were the main cause. Jiang et al. (2014) developed a double haploid (DH) population and two BCF_1_ populations to detect QTLs for the chlorophyll content of rice leaves. Their results showed that overdominance could adequately explain the genetic basis of heterosis in rice.

An “immortalized F_2_” (IF_2_) population derived from pair crosses of RILs was constructed; some researchers used it to detect heterotic loci (HL), instead of traditional QTLs, to explain the genetic basis of complete or partial dominance, overdominance, and epistasis (Hua et al., 2002, 2003; Tang et al., 2010; Zhou et al., 2012; Guo et al., 2014). Hua et al. (2002, 2003) first introduced the rice IF_2_ population and discovered that heterotic effects at the single-locus level, in combination with dominance by dominance epistatic effects, were the main cause of the genetic basis of yield heterosis. In maize, based on an IF_2_ population, Tang et al. (2010) demonstrated that dominance effects of HL, at the single-locus level as well as additive × additive interactions, was important for heterosis of grain yield and its components. With the same experimental design and materials, Guo et al. (2014) reanalyzed the genetic basis of yield heterosis based on a reconstructed high-density linkage map. The results showed that dominance contributed more to heterosis than to other genetic effects among all traits. In addition, overdominance and epistasis contributed to yield heterosis as well.

Both IF_2_ and BCF_1_ populations are good materials for studying heterosis, owing to the advantages of stable genotype and repeatable experiments, which can detect additive and dominant effects simultaneously. Usually, stably expressed QTLs in multiple environments are highly favored in MAS. Thus, constructing different segregating populations from the same parental combination, identifying QTLs, and evaluating their expression levels and genetic basis of heterosis under multiple environments will allow us to map stable QTLs and accelerate the breeding process of better fiber quality species. Recently, a few studies on QTL mapping across multiple populations have been reported (Shen et al., 2005; Sun et al., 2012; Yu et al., 2013; Zhang et al., 2015), but the study of heterosis with related segregating populations has not been reported in upland cotton.

Most of the previous studies have focused on the heterosis of yield traits in upland cotton (Meredith, 1990; Guo et al., 2013; Shang et al., 2016), but little attention has been paid to the heterosis of fiber traits (Meredith and Bridge, 1972; Meredith, 1990; Tang et al., 1993). In the present study, based on the high-density SNP intraspecific genetic map, the IF_2_ population, two BCF_1_ populations, and their midparental heterosis (MPH) datasets were used simultaneously to analyze the genetic effects of heterosis for fiber quality traits. The main objectives were to characterize the genetic components in cotton, including additive effect, partial dominance/dominance, overdominance, and epistasis as well as their relative contributions to fiber quality heterosis. This study may have meaningful implications in exploring the genetic basis of fiber quality heterosis in upland cotton.

## Materials and methods

### Plant materials and construction of population

A population of 188 F_8_ RILs, derived by a modified single-seed procedure (Wu et al., 2008) from a cross between two elite upland cotton germplasms, HS46 and MARCABUCAG8US-1-88, was used to produce three new genetic populations based on the experimental design. To produce the IF_2_ population, 188 RILs were planted randomly and 188 crosses were made as follows: the 1st line was used as the female crossed with the 2nd line to produce the 1st cross, and the 2nd line was used as the female at the same time crossed with the 3rd line to produce the 2nd cross. Thus, each RIL was used as a female parent in one cross and a male parent in another cross. This procedure was repeated twice and a total of 376 crosses were developed to form the IF_2_ population, including 376 hybrids. The second and third populations were two backcross populations derived from a modified Design III based on RILs (Comstock and Robinson, 1952; Frascaroli et al., 2007), in which two parents were used as the male parents backcrossed with the RILs. Each of the two backcross populations contained 188 lines named as HSBCF_1_ and MARBCF_1_, referring to 188 RILs backcrossed with HS46 (HS) and MARCABUCAG8US-1-88 (MAR), respectively.

### Field planting and phenotypic measurement

Parent, IF_2_, and the two BCF_1_ populations were planted in two different regions, Yacheng (inland climate) and Baogang (coastal climate) of Sanya, Hainan Province, China, during two winter seasons of 2014 and 2015. Each location and the populations of IF_2_, HSBCF_1_, and MARBCF_1_ were arranged independently in the same field; each population was evaluated in a completely randomized block design with two replications. Each plot included one row of 5.6 m^2^. Management of the fields followed normal agricultural practices.

In each line, 20 normally open bolls were hand-harvested to measure fiber quality traits with HVI 1000 (USTER®HVISPECTRUM, SPINLAB, United States) in the Cotton Quality Supervision, Inspection and Testing Center, Ministry of Agriculture, Anyang, Henan province, China. Fiber quality traits were fiber length (FL, mm), fiber length uniformity (FU, %), micronaire (MIC), fiber elongation (FE, %), and fiber strength (FS, cN.tex^−1^).

### Genotype analysis and linkage maps

The molecular marker data for the RIL population were as previously described (Li et al., 2016). A total of 3,120 SNP markers were selected to genotype the RILs, and a high-density linkage map was constructed, including 2,618 loci with a total length of 1784.28 cM. The genotypes for each cross in the IF_2_, HSBCF_1_, and MARBCF_1_ populations were deduced from the RILs and the original parents that were used as the parents for the crosses.

### Data analysis and QTL mapping

Each year-location was analyzed as an independent environment. A one-way ANOVA was performed to calculate the significance of difference for each trait between the two parent lines, and descriptive statistics, including mean value, maximal value, and minimal value, was performed to analyze the univariate phenotypic data of the IF_2_, HSBCF_1_, and MARBCF_1_ populations using SPSS 20.0. Broad-sense heritability (*H*^2^) was estimated as *H*^2^ = *V*_G_/(*V*_*G*_+ *V*_*GE*_ */e*+ *V*_ε_*/re*), where *V*_G_ is genetic variance, *V*_*GE*_ is genotype × environment interaction variance, *V*_ε_ is error variance, and *e* and *r* are the numbers of environments and replicates, respectively. The minimum norm quadratic unbiased estimation (MINQUE) approach was used to estimate the *V*_*G*_, *V*_*GE*_, and *V*_ε_ (Zhu, 1989) using QGA Station 2.0 (http://ibi.zju.edu.cn/software/index.html).

The MPH of each F_1_ in the IF_2_, HSBCF_1_, and MARBCF_1_ populations was estimated as MPH = F_1_ − MP (Hua et al., 2003), and the MPH percent was calculated as MPH (%) = 100 × (F_1_ − MP)/MP, where F_1_ represented the observations of each line in the IF_2_, HSBCF_1_, and MARBCF_1_ populations, and MP represented the average trait value between the corresponding parents. The direct measured trait values and MPH of the IF_2_, HSBCF_1_, and MARBCF_1_ populations were used separately as input data in each environment (Mei et al., 2005).

The analysis of QTL was conducted independently for the IF_2_, HSBCF_1_, and MARBCF_1_ experiments. Single-locus QTL detection was performed with the composite interval mapping (CIM) approach using the WinQTL Cartographer 2.5 (Wang et al., 2012). The logarithm of odds (LOD) threshold of significant QTLs was performed by 1,000 permutation tests (*P* < 0.05). The MPH datasets only identified the dominance effect under the genetic model of CIM, where the QTLs showed significant difference in heterosis between F_1_ hybrids and the mean values of their two parents (Hua et al., 2003). The QTLs were named as “q + trait abbreviation + chromosome number + QTL number.” A diagrammatic representation of the genetic map and QTLs was made using Map Chart 2.2 (Voorrips, 2002).

The definition of gene actions in the IF_2_ and BCF_1_ populations were as follows: *a* = (P_1_P_1_ − P_2_P_2_)/2, *d* = (P_1_P_2_ − (P_1_P_1_ + P_2_P_2_)/2), BCF_1_ = (*a* + *d*). Here, P_1_ and P_2_ indicate the parents, P_1_P_1_ and P_2_P_2_ stand for the effects of homozygous genotype observed in the IF_2_ or BCF_1_ populations, and P_1_P_2_ indicates the effects of the heterozygous genotype in hybrids. The mode of action was estimated for each QTL based on the absolute value of the ratio of dominance and additive effects (*|d/a|*) (Luo et al., 2009; Liu et al., 2011; Guo et al., 2013; Shang et al., 2016). The assessment of the degree of dominance showed that difference existed between the IF_2_ and BCF_1_ populations. For the IF_2_ population, if |*d*/*a*| > 1 or if it was only identified in MPH data, the QTL was considered as an overdominant locus. Otherwise, the QTLs were considered to be a complete or partial dominant locus. |*d*/*a*| was estimated in two ways, both *a* and *d* were estimated from the QTL detected in the IF_2_ dataset when a QTL was only found in the IF_2_ dataset; *a* was from the RILs and *d* was from the MPH dataset for a QTL detected simultaneously in the RILs and the IF_2_MPH datasets and not for the IF_2_ dataset. When a QTL was present in all three datasets, the value of |*d*/*a*| in the IF_2_ dataset is the criterion. For the BCF_1_ populations, the overdominant locus was expected for a QTL meeting the following conditions: (1) only detectable for MPH dataset; (2) 2 × *d* in the MPH dataset was higher than an estimate in the BCF_1_ performance (*a* + *d*), that is, 2|*d*| (MPHs) > |*a* + *d*| (BCF_1_s) (equal to |*d/a*| > 1); (3) *a* was from the RILs and *d* was from the MPH dataset with |*d/a*| >1 for a QTL detected simultaneously in the RILs and the MPH dataset. Otherwise, the QTL was referred to as a complete or partial dominant locus. The QTLs detected only in the BCF_1_ dataset were referred to as additive. When a QTL was present in all three datasets, the calculated value based on the BCF_1_ dataset and MPH dataset is the criterion. The RILs data were from our previous report (Li et al., 2016).

Based on the direct measurements of the traits and MPH datasets of the IF_2_, HSBCF_1_, and MARBCF_1_ populations, a combined multiple-environment model analysis that tests the main-effect QTLs (m-QTLs), epistatic QTLs (e-QTLs), and their environmental interactions (QTL × environment, QE), was performed with the inclusive composite interval mapping (ICIM) method using IciMapping 4.1 (Li et al., 2007). The analyses of additive effect and epistasis were performed with pre-adjusted IciMapping parameters, Scan = 1 cM/PIN = 0.0001 and Scan = 5 cM/PIN = 0.0001, respectively. The threshold LOD score for declaring m-QTLs and e-QTLs was implemented by a 1,000-permutation test (*P* < 0.05). The naming of the detected m-QTLs used the form “dataset abbreviation + maq (multi-environment additive QTL) + trait abbreviation – chromosome number – QTL number.” The e-QTLs identified were named using the dataset abbreviation, followed by “meq” (multi-environment epistatic QTL) and then with the abbreviation of trait and, finally, the QTL pair number. The details of dataset abbreviations were as follows: the IF_2_, HSBCF_1_, and MARBCF_1_ populations were abbreviated to “I,” “B_1_,” and “B_2_,” respectively, and their corresponding MPH datasets were denoted by adding “M” after the population abbreviation, that is, “IM,” “B_1_M,” “B_2_M.”

## Results

### Performance of fiber quality traits

The measurements of fiber quality traits for the IF_2_, HSBCF_1_, and MARBCF_1_ populations as well as for the two parents are shown in Table 1. Except for FE, the other fiber quality traits of parent HS46 were significantly better than that of another parent, MARCABUCAG8US-1-88. In the IF_2_, HSBCF_1_, and MARBCF_1_ populations, a wide range of variation was found in fiber quality traits (Table 1). Furthermore, in all four environments, obvious transgressive segregation was observed.

**Table 1:** Phenotypic variation of fiber quality traits for the upland cotton IF_2_, HSBCF_1_, and MARBCF_1_ populations and their parents.

**Traits^a^**	**Environment^b^**	**Parents^c^**					**IF**_**2**_**s**			**HSBCF**_**1**_**s**			**MARBCF**_**1**_**s**		
		**P_1_**	**P_2_**	**MP**	**P_1_-P_2_**	***P*-value**	**Mean**	**Min**	**Max**	**Mean**	**Min**	**Max**	**Mean**	**Min**	**Max**
FL	2014Yc	31.00	29.84	30.42	1.17	0.0000	31.67	27.38	34.36	31.81	28.71	34.41	31.90	28.43	34.28
	2014Bg	30.47	29.17	29.82	1.31		30.47	27.38	33.64	30.57	28.40	33.10	30.47	27.80	32.50
	2015Yc	31.62	30.21	30.91	1.41		31.56	28.10	34.50	32.14	29.30	35.10	31.56	28.90	34.60
	2015Bg	31.51	30.19	30.85	1.32		31.50	28.00	34.70	31.98	29.30	34.90	31.96	28.90	34.80
FU	2014Yc	86.35	85.27	85.81	1.08	0.0000	87.21	84.60	90.00	87.08	84.30	90.10	87.28	84.10	89.90
	2014Bg	85.92	84.90	85.41	1.02		85.68	82.60	89.60	85.57	83.00	88.20	85.61	82.80	87.90
	2015Yc	86.61	85.59	86.10	1.02		86.09	82.90	88.40	84.93	81.10	88.40	85.62	82.10	88.30
	2015Bg	86.31	84.52	85.42	1.78		85.66	81.40	88.80	85.18	76.50	87.30	85.14	79.40	88.20
MIC	2014Yc	4.12	3.77	3.95	0.36	0.0079	4.41	3.07	5.34	4.04	3.22	4.87	4.18	3.47	5.13
	2014Bg	3.88	3.75	3.81	0.13		4.02	2.94	5.03	3.80	2.80	4.80	3.84	2.90	4.60
	2015Yc	4.24	4.12	4.18	0.12		4.18	2.40	5.20	4.03	3.10	4.80	3.99	2.60	4.90
	2015Bg	4.02	3.81	3.91	0.21		3.86	2.30	5.50	3.81	2.70	4.50	3.82	2.20	4.90
FE	2014Yc	6.43	6.52	6.47	−0.08	0.8636	5.85	4.00	8.20	5.20	3.60	8.70	5.40	3.90	7.00
	2014Bg	5.98	6.07	6.02	−0.09		6.16	4.30	9.40	5.61	4.30	7.20	5.66	4.20	8.00
	2015Yc	6.98	6.90	6.94	0.08		6.98	6.70	7.20	6.94	6.70	7.10	6.94	6.70	7.20
	2015Bg	7.03	6.94	6.98	0.09		6.95	6.60	7.20	6.98	6.70	7.20	7.00	6.50	7.30
FS	2014Yc	30.09	28.09	29.09	2.01	0.0000	31.82	27.40	37.70	32.13	28.60	38.60	31.89	28.40	37.50
	2014Bg	29.45	28.67	29.06	0.78		30.64	26.17	36.36	30.67	27.00	36.00	30.20	25.30	37.50
	2015Yc	32.84	30.46	31.65	2.38		32.77	28.70	39.30	32.76	28.40	37.90	32.78	26.80	38.90
	2015Bg	33.15	30.41	31.78	2.75		32.76	24.80	38.10	33.04	27.00	38.00	33.16	25.80	37.90

a FL, fiber length; FU, fiber uniformity; MIC, micronaire; FE, fiber elongation; FS, fiber strength.

b 2014Yc, Yacheng of Hainan Province in 2014; 2014Bg, Baogang of Hainan Province in 2014; 2015Yc, Yacheng of Hainan Province in 2015; 2015Bg, Baogang of Hainan Province in 2015.

c *P_1_, HS56; P_2_, MARCABUCAG8US-1-88*.

In the IF_2_, HSBCF_1_, and MARBCF_1_ populations, the heterosis value varied widely in all fiber quality traits, that is, from negative to highly positive (Table 2 and Supplementary Table S1). Higher levels and positive heterosis for FL, MIC, FS, and FU were observed in the IF_2_ and two BCF_1_ populations, whereas FE showed lower levels or negative heterosis in these three populations.

**Table 2:** MPH percent of fiber quality traits in IF_2_, HSBCF_1_, and MARBCF_1_ populations across four environments.

**Traits^a^**	**IF**_**2**_**MPHs (%)**			**HSBCF**_**1**_**MPHs (%)**			**MARBCF**_**1**_**MPHs (%)**		
	**Mean**	**Min**	**Max**	**Mean**	**Min**	**Max**	**Mean**	**Min**	**Max**
FL	5.51	−10.94	73.51	9.22	−77.98	125.59	12.77	−50.46	130.07
FU	2.71	−5.18	35.53	9.16	−64.44	41.68	8.24	−5.94	33.47
MIC	16.54	−42.50	93.35	8.71	−24.34	49.56	10.23	−18.55	57.06
FE	2.14	−37.50	74.07	−0.04	−42.12	38.57	−0.71	−39.60	52.42
FS	9.37	−22.62	46.42	9.58	−10.07	56.01	9.83	−11.44	47.44

a *FL, fiber length; FU, fiber uniformity; MIC, micronaire; FE, fiber elongation; FS, fiber strength*.

Some differences in heterosis for the same trait were found in different populations across the four environments. For FL, the order of the mean values of heterosis was MARBCF_1_ (12.77%) > HSBCF_1_ (9.22%) > IF_2_ (5.51%). The mean values of heterosis of FS showed the same trend as FL in different populations, which were 9.83, 9.58, and 9.37% in the IF_2_, HSBCF_1_, and MARBCF_1_ populations, respectively. For FU, the HSBCF_1_ (9.16%) and MARBCF_1_ (8.24%) populations showed higher heterosis, whereas the IF_2_ population exhibited lower heterosis (2.71%). For MIC, high levels of heterosis were observed in all three populations, and the order of the mean values of heterosis was IF_2_ (16.54%) > MARBCF_1_ (10.23%) > HSBCF_1_ (8.71%). In contrast, FE exhibited low levels of heterosis in the IF_2_ populations and negative heterosis in the two BCF_1_ populations.

Several inconsistencies were found between the different environments (Supplementary Table S1). In all three populations, lower MPH (%) was observed for MIC and FS in 2015Yc and 2015Bg than in 2014Yc and 2014Bg. In the 2014Yc environment, the heterosis values were 0.53 (18.98%), 0.45 (14.18%), and 0.68 (15.14%) for MIC in the IF_2_, HSBCF_1_, and MARBCF_1_ populations, respectively, and they were 0.49 (19.64%), 0.41 (12.54%), and 0.39 (12.66%), respectively, in the 2014Bg environment; whereas in the 2015Yc and 2015Bg environments, the heterosis values of MIC in all three populations were lower. The same trend was found for FS, probably attributed to high temperatures and rainy conditions in 2015 in Sanya, which affected the cotton fiber development. Low levels or negative heterosis was exhibited by FE in all environments, probably due to the lack of significant difference between the two original parents.

Within each of the populations, MPH values of hybrids varied considerably (Supplementary Table S2). Most of the trait values of extreme lines exceeded those of the MP value of their parents and showed high MPH in all environments. For example, the mean heterosis of the top 10 high-heterosis hybrids of MIC were more than 30% in the four environments of all three populations, except in the 2014Yc experiment of the HSBCF_1_ population.

The broad-sense heritability was also analyzed using measurement data of the four environments (Table 3). In the IF_2_, HSBCF_1_, and MARBCF_1_ populations, data related to fiber quality exhibited a similar range of heritability from 30.02 to 79.60%, 24.58 to 80.96%, and 26.87 to 80.05%, respectively, which showed significant genetic and environmental effects. Fiber length had almost the highest heritability in all three populations, which was generally consistent with the literature (Qin et al., 2008; Wang et al., 2015; Li et al., 2016). Interestingly, the heritability of all traits in the two BCF_1_ populations was highly consistent, which might be related to their closer genetic basis.

**Table 3:** Analysis of variance (ANOVA) for fiber quality traits in three populations across four environments.

**Population**	**Components of variation^a^**	**Traits ^b^**				
		**FL**	**FU**	**MIC**	**FE**	**FS**
IF_2_	*V_*G*_*	0.697	0.183	0.068	0.029	1.056
	*V _*GE*_*	0.004	0.202	0.001	0.106	0.146
	*V_*e*_*	1.421	1.984	0.268	0.340	5.561
	*H^2^* (%)	79.60	37.92	66.54	30.02	59.07
HSBCF_1_	*V_*G*_*	0.762	0.109	0.056	0.039	1.019
	*V _*GE*_*	0.031	0.335	0.011	0.059	0.153
	*V_*e*_*	1.372	2.012	0.270	0.345	5.729
	*H^2^* (%)	80.96	24.58	60.45	40.42	57.47
MARBCF_1_	*V_*G*_*	0.733	0.122	0.059	0.043	0.884
	*V _*GE*_*	0.045	0.324	0.001	0.052	0.163
	*V_*e*_*	1.371	2.014	0.270	0.345	5.737
	*H^2^* (%)	80.05	26.87	63.43	43.36	53.83

a *V_G_, genetic variance; V_GE_, genotype × environment interaction variance; Ve, error variance; H^2^, the broad-sense heritability*.

b *FL, fiber length; FU, fiber uniformity; MIC, micronaire; FE, fiber elongation; FS, fiber strength*.

### QTL analysis of fiber quality in IF_2_ population, two BCF_1_ populations, and their MPH datasets

**Figure 1:**
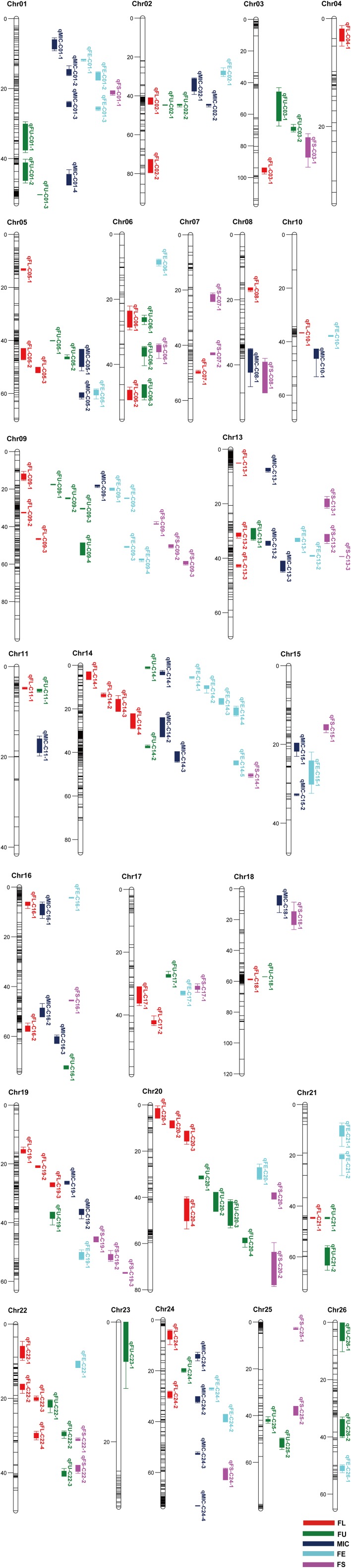
Chromosomal location of QTLs for fiber quality traits in IF_2_, HSBCF_1_, MARBCF_1_, IF_2_MPH, HSBCF_1_MPH, and MARBCF_1_MPH datasets across four environments. Map distances were given in centimorgans (cM). Solid bars with different colors represent different traits, and the legend is given at the end of figure. FL, fiber length; FU, fiber uniformity; MIC, micronaire; FE, fiber elongation; FS, fiber strength.

A genetic map was constructed in our previous study (Li et al., 2016). A total of 167 QTLs related to fiber quality were detected by CIM analysis in the IF_2_, HSBCF_1_, MARBCF_1_ datasets and their MPH datasets, explaining 3.00–24.73% of the total phenotypic variation (PV) (Figure 1 and Supplementary Table S3). Among the 167 QTLs, 68 QTLs were detected in more than two datasets or environments, 31 of which were detected in both years (Table 4).

**Table 4:** The detailed information of stable QTLs identified by CIM method.

**Trait^a^**	**QTL^b^**	**Env.^c^**	**Flanking markers**	**Position^d^**	**LOD^e^**	**A^f^**	**D^f^**	**A+D^f^**	**R^2^(%)^g^**	**Population**
FL	**qFL-C02-1**	2014Bg	i43421Gh-i24299Gh	41.21	5.00		−2.15		19.31	MARBCF_1_MPH
		2015Bg	i14776Gh-i16398Gh	43.91	2.59		−2.19		12.20	IF_2_MPH
	qFL-C02-2	2015Bg	i02276Gh-i01044Gh	75.71	2.80		0.56		6.28	IF_2_MPH
		2015Yc	i49488Gh-i14841Gh	78.11	3.03		0.18		9.51	HSBCF_1_MPH
	**qFL-C05-2**	2015Bg	i16671Gh-i29825Gh	43.21	3.39			0.04	7.96	HSBCF_1_
		2014Bg	i19536Gh-i34270Gh	46.91	2.59			0.03	5.90	MARBCF_1_
	**qFL-C05-3**	2014Yc	i16666Gh-i51323Gb	50.81	3.37		0.76		13.32	HSBCF_1_MPH
		2015Bg	i16666Gh-i22016Gh	50.81	4.60			0.24	11.52	HSBCF_1_
		2014Yc	i09095Gh-i46446Gh	51.61	3.48			1.25	9.71	HSBCF_1_
	**qFL-C06-1**	2015Bg	i21566Gh-i14061Gh	25.61	2.65		0.49		14.64	IF_2_MPH
		2014Bg	i06526Gh-i34827Gh	27.81	2.93		−2.47		10.60	MARBCF_1_MPH
		2014Yc	i06526Gh-i34827Gh	27.81	2.54	2.78	−2.86		20.89	IF_2_
	qFL-C09-2	2015Bg	i23159Gh-i47527Gh	32.71	4.78	0.48	−0.14		11.72	IF_2_
		2015Yc	i23159Gh-i47527Gh	32.71	3.90	0.36	−0.39		9.54	IF_2_
	**qFL-C14-1**	2014Bg	i05482Gh-i18840Gh	4.01	4.21		−2.61		19.34	MARBCF_1_MPH
		2014Yc	i05482Gh-i04837Gh	4.01	4.19	2.85	−2.75		16.87	IF_2_
		2015Bg	i22641Gh-i48509Gh	6.31	3.25	0.26	1.77		3.97	IF_2_
	**qFL-C14-3**	2015Yc	i15343Gh-i15345Gh	18.41	2.69		−0.30		7.93	MARBCF_1_MPH
		2014Yc	i18849Gh-i00465Gh	20.91	3.02			−0.19	8.31	MARBCF_1_
		2015Bg	i15345Gh-i00465Gh	20.91	5.39			−1.09	4.99	MARBCF_1_
	**qFL-C14-4**	2015Bg	i15340Gh-i34657Gh	23.21	3.84			−0.83	3.14	HSBCF_1_
		2015Bg	i34657Gh-i40518Gh	24.71	5.25			−1.18	3.01	MARBCF_1_
		2014Yc	i05040Gh-i31578Gh	28.21	4.93			−0.66	10.42	MARBCF_1_
	qFL-C16-2	2015Yc	i21384Gh-i42534Gh	57.01	3.28	−0.32	0.15		7.12	IF_2_
		2015Bg	i21384Gh-i31338Gh	57.21	2.68	−0.10	0.71		3.22	IF_2_
		2015Bg	i21384Gh-i42534Gh	57.21	4.20		0.79		6.93	IF_2_MPH
	qFL-C17-1	2014Bg	i03509Gh-i14513Gh	31.31	2.97			−0.08	8.61	HSBCF_1_
		2014Yc	i03218Gh-i03508Gh	35.11	2.79	2.74	−2.99		17.58	IF_2_
	qFL-C18-1	2014Bg	i31525Gh-i26380Gh	58.61	4.72		−2.30		20.92	MARBCF_1_MPH
		2014Bg	i48138Gh-i13492Gh	59.01	3.25		−2.68		10.35	HSBCF_1_MPH
	qFL-C19-1	2014Yc	i28797Gh-i50906Gb	16.01	5.48			0.51	11.35	HSBCF_1_
		2014Yc	i28797Gh-i50906Gb	16.01	4.91		0.80		10.36	HSBCF_1_MPH
	**qFL-C19-3**	2014Yc	i55376Gb-i37157Gh	26.51	2.52			−0.73	7.64	HSBCF_1_
		2015Yc	i47122Gh-i08962Gh	27.61	5.76			2.15	12.42	MARBCF_1_
	**qFL-C20-2**	2014Bg	i11727Gh-i39228Gh	8.01	2.76		−0.61		4.99	IF_2_MPH
		2015Bg	i11723Gh-i34769Gh	8.01	3.82	−0.81	−0.28		10.13	IF_2_
		2015Yc	i11723Gh-i34769Gh	9.91	5.82	−0.45	−0.39		5.69	IF_2_
	**qFL-C20-4**	2014Bg	i47006Gh-i17500Gh	41.51	4.07			0.33	7.94	MARBCF_1_
		2015Yc	i26441Gh-i17500Gh	41.51	3.29			0.12	8.81	MARBCF_1_
		2014Yc	i17505Gh-i47439Gh	42.11	2.51			−0.3058	6.26	HSBCF_1_
		2015Bg	i17505Gh-i47439Gh	44.11	3.10			−0.4511	9.26	HSBCF_1_
FU	**qFU-C01-1**	2015Yc	i23213Gh-i36727Gh	30.31	3.79			0.89	5.10	HSBCF_1_
		2014Yc	i42430Gh-i55243Gb	35.71	4.26	1.43	−1.45		13.61	IF_2_
	**qFU-C01-2**	2014Bg	i31455Gh-i02245Gh	41.71	2.52			0.50	5.43	HSBCF_1_
		2015Bg	i02245Gh-i02457Gh	45.71	7.09			−4.99	15.22	HSBCF_1_
		2015Bg	i02245Gh-i02457Gh	45.71	6.83		−7.40		15.20	HSBCF_1_MPH
	qFU-C02-1	2015Bg	i02758Gh-i02723Gh	44.81	5.77			−4.44	8.27	HSBCF_1_
		2015Bg	i02758Gh-i02723Gh	44.81	3.75		−6.04		8.84	HSBCF_1_MPH
	qFU-C03-1	2015Bg	i35903Gh-i39896Gh	50.51	2.99	−0.76	0.64		19.03	IF_2_
		2015Bg	i35903Gh-i39896Gh	52.51	3.17		0.48		14.96	IF_2_MPH
	qFU-C05-2	2015Bg	i19536Gh-i34270Gh	46.91	3.98	−0.33	0.65		9.57	IF_2_
		2015Bg	i19536Gh-i34270Gh	46.91	2.72		0.64		5.57	IF_2_MPH
	qFU-C06-2	2015Bg	i06036Gh-i06037Gh	35.01	11.44			0.29	18.73	HSBCF_1_
		2015Yc	i06036Gh-i23722Gh	36.01	2.84	−0.40	1.06		9.36	IF_2_
		2015Yc	i06037Gh-i06505Gh	36.01	3.91		−0.90		3.89	HSBCF_1_MPH
		2015Yc	i06037Gh-i23722Gh	36.01	3.13		1.12		3.70	IF_2_MPH
	**qFU-C06-3**	2015Bg	i23722Gh-i06396Gh	47.21	8.43			0.19	19.69	HSBCF_1_
		2014Yc	i37862Gh-i06396Gh	49.21	3.15			0.70	8.63	MARBCF_1_
	**qFU-C09-4**	2014Yc	i41596Gh-i35858Gh	49.61	4.85			1.35	14.97	HSBCF_1_
		2015Bg	i07773Gh-i15768Gh	53.01	10.82			9.05	3.00	HSBCF_1_
		2014Bg	i18484Gh-i03595Gh	54.71	2.70	0.30	0.08		5.16	IF_2_
	qFU-C11-1	2015Bg	i33855Gh-i43823Gh	5.31	3.48	−1.99	3.20		9.25	IF_2_
		2015Bg	i33855Gh-i43823Gh	5.31	3.69		3.44		10.43	IF_2_MPH
	qFU-C13-1	2014Yc	i23966Gh-i29670Gh	29.11	2.60			2.32	3.68	MARBCF_1_
		2014Bg	i24929Gh-i27668Gh	30.21	3.25	−0.38	0.07		8.51	IF_2_
		2014Bg	i32083Gh-i62433Gt	32.41	2.69			0.31	7.21	HSBCF_1_
	qFU-C14-1	2015Bg	i15536Gh-i05487Gh	1.11	3.46	−3.22	3.31		11.65	IF_2_
		2015Bg	i15536Gh-i05487Gh	1.11	3.74		4.14		11.10	IF_2_MPH
	qFU-C16-1	2015Bg	i54704Gb-i01693Gh	72.91	10.20			−0.04	21.83	HSBCF_1_
		2015Bg	i54704Gb-i01693Gh	72.91	9.97		4.93		19.23	HSBCF_1_MPH
	**qFU-C20-2**	2015Bg	i11714Gh-i37554Gh	38.01	11.32			−0.09	11.59	HSBCF_1_
		2015Bg	i11714Gh-i37554Gh	38.01	11.31		4.69		11.01	HSBCF_1_MPH
		2014Yc	i11912Gh-i47439Gh	41.51	5.63	−0.34	−0.38		7.75	IF_2_
	qFU-C20-4	2015Bg	i18012Gh-i11915Gh	57.81	3.77			−3.54	9.36	HSBCF_1_
		2015Bg	i18012Gh-i11478Gh	57.81	3.68		−5.39		10.76	HSBCF_1_MPH
	qFU-C21-1	2015Bg	i16082Gh-i00284Gh	44.61	2.86		−1.43		13.42	MARBCF_1_MPH
		2015Yc	i16082Gh-i00284Gh	44.61	2.69		0.01		6.98	IF_2_MPH
	qFU-C21-2	2015Yc	i41432Gh-i22642Gh	58.91	3.07		−0.10		7.32	IF_2_MPH
		2015Yc	i07219Gh-i41613Gh	59.91	3.10		0.63		3.58	MARBCF_1_MPH
	qFU-C23-1	2015Bg	i06287Gh-i06171Gh	0.01	5.57			−1.10	9.23	MARBCF_1_
		2015Bg	i06287Gh-i06171Gh	0.01	3.26		−1.32		5.23	MARBCF_1_MPH
	qFU-C26-1	2015Bg	i00879Gh-i32452Gh	1.01	3.75		−0.59		10.03	MARBCF_1_MPH
		2015Yc	i00879Gh-i33827Gh	2.51	3.32		0.39		10.82	HSBCF_1_MPH
	qFU-C26-2	2014Bg	i08565Gh-i36067Gh	35.01	3.37			0.42	8.28	MARBCF_1_
		2014Yc	i08565Gh-i08578Gh	38.31	3.64			0.32	10.01	HSBCF_1_
MIC	qMIC-C01-1	2015Bg	i60883Gt-i48104Gh	6.21	2.69			−0.22	3.09	MARBCF_1_
		2015Bg	i31143Gh-i21823Gh	7.51	2.68	0.05	−0.21		3.19	IF_2_
	qMIC-C01-4	2015Yc	i02245Gh-i02767Gh	45.71	3.28	−0.30	−0.26		3.86	IF_2_
		2015Yc	i02245Gh-i44115Gh	45.71	3.74		−0.32		3.88	IF_2_MPH
	qMIC-C02-1	2015Yc	i16954Gh-i20804Gh	32.21	3.14			0.97	4.20	HSBCF_1_
		2015Yc	i18644Gh-i27649Gh	35.81	3.98			0.73	4.24	MARBCF_1_
		2015Yc	i18644Gh-i27649Gh	35.81	4.17		0.33		4.35	MARBCF_1_MPH
	**qMIC-C05-1**	2015Bg	i09071Gh-i01144Gh	44.11	2.69			0.4179	4.46	MARBCF_1_
		2014Yc	i53822Gb-i01144Gh	44.41	3.53			0.0719	4.55	MARBCF_1_
		2014Bg	i00341Gh-i31875Gh	47.21	3.39			0.0844	4.66	MARBCF_1_
		2014Bg	i31875Gh-i16666Gh	48.31	2.66			0.53	4.78	HSBCF_1_
	**qMIC-C08-1**	2015Yc	i30195Gh-i04557Gh	35.61	3.02		−0.70		5.78	IF_2_MPH
		2015Bg	i30195Gh-i04565Gh	35.91	3.11		0.68		5.88	HSBCF_1_MPH
		2014Yc	i40070Gh-i01126Gh	38.01	2.91		−0.58		5.89	IF_2_MPH
	qMIC-C11-1	2015Yc	i07468Gh-i36064Gh	17.11	2.97			0.41	6.25	HSBCF_1_
		2015Yc	i07468Gh-i36064Gh	18.11	3.96		0.01		6.29	HSBCF_1_MPH
	**qMIC-C13-3**	2015Bg	i42046Gh-i38620Gh	40.91	3.39	−0.12	0.12		6.81	IF_2_
		2014Yc	i49771Gh-i35111Gh	44.11	2.94			0.08	7.03	HSBCF_1_
	**qMIC-C14-2**	2014Yc	i05007Gh-i40518Gh	25.71	2.68			0.13	7.56	MARBCF_1_
		2014Yc	i15375Gh-i40518Gh	27.21	2.91			0.21	7.83	HSBCF_1_
		2015Bg	i44975Gh-i34413Gh	30.51	4.21			0.27	7.99	MARBCF_1_
		2015Bg	i43013Gh-i44046Gh	30.91	2.85			0.01	8.13	HSBCF_1_
		2014Yc	i66845Ga-i05256Gh	31.21	2.66	0.07	−0.22		8.17	IF_2_
		2015Yc	i23352Gh-i39672Gh	32.71	2.78			0.30	8.34	HSBCF_1_
	qMIC-C14-3	2015Bg	i22394Gh-i41891Gh	40.21	2.74			0.19	8.62	MARBCF_1_
		2015Yc	i28729Gh-i00245Gh	41.01	2.92			0.10	8.77	MARBCF_1_
		2015Yc	i23762Gh-i38809Gh	43.81	3.65			−0.01	9.73	HSBCF_1_
	qMIC-C15-1	2015Bg	i29719Gh-i49465Gh	20.51	5.66			0.27	9.83	HSBCF_1_
		2015Bg	i29719Gh-i49465Gh	20.51	5.05		0.07		9.85	HSBCF_1_MPH
		2015Yc	i29719Gh-i49465Gh	20.51	3.30		0.07		10.16	HSBCF_1_MPH
	**qMIC-C16-2**	2015Bg	i34919Gh-i45501Gh	48.91	2.56			0.46	11.93	MARBCF_1_
		2014Bg	i46435Gh-i00787Gh	51.61	3.31			0.13	11.93	MARBCF_1_
	**qMIC-C19-1**	2014Yc	i27871Gh-i09066Gh	26.21	2.73			−0.06	16.14	HSBCF_1_
		2015Bg	i55376Gb-i37157Gh	26.51	3.20			1.91	17.13	MARBCF_1_
FE	**qFE-C01-2**	2015Bg	i02201Gh-i32863Gh	15.91	6.42		0.19		4.30	HSBCF_1_MPH
		2014Yc	i23944Gh-i39024Gh	17.41	3.65		−0.70		24.18	IF_2_MPH
	**qFE-C02-1**	2015Yc	i17680Gh-i02712Gh	23.11	2.71		0.07		5.51	IF_2_MPH
		2014Yc	i02712Gh-i20804Gh	27.31	2.86		1.23		4.70	IF_2_MPH
	qFE-C05-1	2015Bg	i20652Gh-i52543Gb	58.91	3.37			0.05	9.03	MARBCF_1_
		2015Yc	i20652Gh-i35017Gh	58.91	2.75			0.07	3.73	MARBCF_1_
	qFE-C10-1	2015Bg	i12268Gh-i32655Gh	38.01	4.06			−0.08	17.04	MARBCF_1_
		2015Bg	i12268Gh-i32655Gh	38.01	4.66		0.09		16.24	MARBCF_1_MPH
	**qFE-C14-1**	2014Yc	i15284Gh-i48509Gh	5.31	2.51			1.75	10.58	HSBCF_1_
		2015Bg	i15284Gh-i48509Gh	5.31	5.79			−0.36	5.25	MARBCF_1_
		2015Bg	i15284Gh-i48509Gh	5.31	5.28		−0.48		5.13	MARBCF_1_MPH
	**qFE-C14-4**	2015Yc	i04916Gh-i05024Gh	20.51	3.19			0.01	9.36	HSBCF_1_
		2014Yc	i40777Gh-i43206Gh	22.11	2.64			−0.07	7.47	MARBCF_1_
	**qFE-C14-5**	2014Yc	i38481Gh-i27231Gh	44.41	4.01		−0.59		10.92	IF_2_MPH
		2015Bg	i38809Gh-i15488Gh	45.01	3.28		−0.11		3.81	MARBCF_1_MPH
	qFE-C15-1	2015Yc	i21698Gh-i24483Gh	25.31	2.62		0.06		5.17	MARBCF_1_MPH
		2015Yc	i25137Gh-i02486Gh	27.81	2.76			−0.05	7.70	HSBCF_1_
	qFE-C19-1	2015Yc	i08832Gh-i09430Gh	51.51	3.70			0.11	4.21	MARBCF_1_
		2015Yc	i08832Gh-i09430Gh	52.21	3.63			0.08	6.73	HSBCF_1_
	qFE-C20-1	2014Bg	i11735Gh-i42616Gh	29.71	2.54			0.01	18.89	HSBCF_1_
		2014Yc	i11735Gh-i42616Gh	29.71	2.66			0.18	3.78	HSBCF_1_
	qFE-C21-1	2015Bg	i06952Gh-i07714Gh	9.91	3.55			−0.04	12.81	MARBCF_1_
		2015Yc	i06952Gh-i07714Gh	10.91	2.80		0.10		17.29	MARBCF_1_MPH
	**qFE-C22-1**	2015Bg	i20168Gh-i39918Gh	10.71	3.07			0.03	10.57	MARBCF_1_
		2014Bg	i30763Gh-i17698Gh	11.61	3.94	1.47	0.12		14.82	IF_2_
	**qFE-C24-2**	2014Bg	i04688Gh-i04069Gh	38.11	4.35		−0.28		8.97	MARBCF_1_MPH
		2015Bg	i31637Gh-i15169Gh	40.11	4.80		0.15		23.41	HSBCF_1_MPH
	qFE-C26-1	2015Bg	i16464Gh-i47876Gh	50.21	3.03			−0.02	16.96	MARBCF_1_
		2015Bg	i28856Gh-i23175Gh	51.31	2.80		−0.35		19.60	MARBCF_1_MPH
FS	qFS-C03-1	2014Yc	i30069Gh-i42939Gh	78.81	3.04			−0.22	8.88	MARBCF_1_
		2014Yc	i43226Gh-i21218Gh	78.81	3.95		−1.19		10.32	MARBCF_1_MPH
	qFS-C07-1	2015Bg	i01696Gh-i01453Gh	21.91	2.64		0.06		9.10	MARBCF_1_MPH
		2015Yc	i01453Gh-i33174Gh	23.21	2.84		1.46		18.41	MARBCF_1_MPH
	qFS-C09-2	2015Yc	i08546Gh-i03687Gh	49.81	3.38	1.17	−1.03		17.70	IF_2_
		2015Yc	i03687Gh-i02498Gh	50.61	3.02		−2.05		17.51	HSBCF_1_MPH
	**qFS-C13-1**	2014Yc	i30934Gh-i18151Gh	19.41	3.54			−0.27	11.50	HSBCF_1_
		2015Bg	i30934Gh-i32650Gh	20.41	2.51			0.19	10.25	MARBCF_1_
	**qFS-C20-1**	2014Yc	i36341Gh-i37554Gh	39.01	2.77			−0.8237	13.06	HSBCF_1_
		2015Bg	i36341Gh-i26441Gh	39.01	2.79			−1.0971	16.94	HSBCF_1_
	qFS-C24-1	2015Yc	i04718Gh-i33113Gh	59.01	2.57		1.23		3.24	MARBCF_1_MPH
		2015Bg	i03705Gh-i03832Gh	60.61	3.04		−0.83		5.05	MARBCF_1_MPH
	**qFS-C25-2**	2014Bg	i29568Gh-i33416Gh	37.01	3.02	0.98	1.99		6.85	IF_2_
		2015Yc	i20999Gh-i22495Gh	39.51	2.62			−3.91	4.27	HSBCF_1_
		2014Yc	i20999Gh-i22495Gh	39.81	2.67	−1.50	−0.71		5.31	IF_2_
		2015Bg	i20999Gh-i22495Gh	39.81	3.70			−3.67	6.19	HSBCF_1_

a *FL, fiber length; FU, fiber uniformity; MIC, micronaire; FE, fiber elongation; FS, fiber strength*.

b *QTLs in bold are those identified in both years*.

c *2014Yc, Yacheng of Hainan Province in 2014; 2014Bg, Baogang of Hainan Province in 2014; 2015Yc, Yacheng of Hainan Province in 2015; 2015Bg, Baogang of Hainan Province in 2015*.

d *Position of QTL located on chromosome: as cM distance from the top of each chromosome*.

e A LOD threshold was used for declaration of QTL based on 1,000 permutations at as significance level of 0.01.

f *The genetic expectation of a QTL effect obtained is the additive effect (A) and dominant effect (D) when estimated from the IF_2_ dataset, the additive and dominance effects (A+D) from the BCF_1_dataset, and the dominance effect (D) from the MPH values*.

g *Phenotypic variance explained by QTL*.

#### Fiber length

A total of 42 QTLs were detected in 6 datasets, explaining 3.00–20.92% of the total PV. Among those, 16 QTLs were identified in at least two datasets or environments (Table 4). Sixteen, twelve, ten, seven, five, and eight QTLs were identified in the IF_2_, HSBCF_1_, MARBCF_1_, IF_2_MPH, HSBCF_1_MPH, and MARBCF_1_MPH datasets, respectively. In the IF_2_ population, five QTLs showed a partial dominant effect and twelve were observed to have an over-dominant effect (Table 5 and Supplementary Table S4). Three QTLs were simultaneously detected in both IF_2_ and IF_2_MPH datasets. The dominant effects of three QTLs (qFL-C09-2, qFL-C14-1, and qFL-C16-2) were uncertain because of their inconsistent mode of action in different environments. In the HSBCF_1_ datasets, 10 QTLs with additive effect and 5 with over-dominant effect were detected, but no QTL with partial or complete dominant effect was detected (Table 5 and Supplementary Table S5). qFL-C05-3 and qFL-C19-1 with apparent over-dominant effect were identified in both the HSBCF_1_ and HSBCF_1_MPH datasets. In the MARBCF_1_ population, 10 QTLs with additive effect and seven with over-dominant effect were observed (Table 5 and Supplementary Table S6). qFL-C14-3 was detected in 2015Yc of the MARBCF_1_MPH dataset and in 2014Yc and 2015Bg of the MARBCF_1_ dataset, with a different mode of action in the two environments of the MARBCF_1_ dataset.

**Table 5:** Gene action of QTL identified for fiber quality traits by CIM across four environments.

**Traits^a^**	**IF**_**2**_**s**				**HSBCF**_**1**_**s**				**MARBCF**_**1**_**s**			
	**A^b^**	**PD/D^b^**	**OD^b^**	**Uncertain^b^**	**A**	**PD/D**	**OD**	**Uncertain**	**A**	**PD/D**	**OD**	**Uncertain**
FL	0	5	12	3	10	0	5	0	9	0	7	1
FU	0	5	15	0	12	0	9	0	5	0	4	0
MIC	0	3	10	0	9	2	1	0	11	1	2	0
FE	0	2	3	0	7	0	4	0	12	0	9	0
FS	0	1	6	1	8	0	2	0	6	0	6	0

a *FL, fiber length; FU, fiber uniformity; MIC, micronaire; FE, fiber elongation; FS, fiber strength*.

b *A, additive effect; PD/D, partial dominant or dominant effect; OD, overdominant effect; Uncertain, QTL with different gene action in different environments*.

#### Fiber uniformity

Thirty-nine QTLs associated with FU were detected in six datasets, explaining 3.00–21.83% of the total PV, among which 19 stable QTLs were identified in more than two datasets or environments (Table 4). Furthermore, five of these stable QTLs were detected in both years. Seventeen, eighteen, six, eight, nine, and four QTLs were identified in the IF_2_, HSBCF_1_, MARBCF_1_, IF_2_MPH, HSBCF_1_MPH, and MARBCF_1_MPH datasets, respectively. In the IF_2_ population, 5 and 15 QTLs were found to have partial dominant effect and over-dominant effect, respectively (Table 5 and Supplementary Table S4). Among them, five QTLs were identified simultaneously in the IF_2_ and IF_2_MPH datasets. In the HSBCF_1_ population, there were 13 and 9 QTLs with additive effect and over-dominant effect, respectively (Table 5 and Supplementary Table S5). Six QTLs were identified simultaneously in the HSBCF_1_ and HSBCF_1_MPH datasets. qFU-C01-2 with apparent over-dominant effect was identified in both 2014Bg and 2015Bg in the HSBCF_1_ dataset, which showed a negative effect originating from MARCABUCAG8US-1-88 in 2014Bg, but a positive effect contributed by HS46 was identified in 2015Bg. In the MARBCF_1_ population, five QTLs with additive effect and four with over-dominant effect were observed (Table 5 and Supplementary Table S6). qFU-C23-1 with over-dominant effect was detected in both the MARBCF_1_ and MARBCF_1_MPH datasets.

#### Micronaire

A total of 30 QTLs were identified, explaining 3.09–22.92% of the total PV. Among those, 12 QTLs were identified in more than two datasets or environments (Table 4). Nine, eleven, twelve, five, three, and three QTLs were identified in the IF_2_, HSBCF_1_, MARBCF_1_, IF_2_MPH, HSBCF_1_MPH, and MARBCF_1_MPH datasets, respectively. In the IF_2_ population, three QTLs exhibited partial dominant effect, while 10 QTLs with |*d*/*a*| > 1 showed apparent over-dominant effect (Table 5 and Supplementary Table S4). qMIC-C01-4 was identified in both the IF_2_ and IF_2_MPH datasets. In the HSBCF_1_ population, nine, two, and one QTLs with additive effect, partial dominant effect, and over-dominant effect were detected, respectively (Table 5 and Supplementary Table S5). qMIC-C11-1 and qMIC-C15-1 were detected in both the HSBCF_1_ and HSBCF_1_MPH datasets. In the MARBCF_1_ population, 11 QTLs with additive effect, one with partial dominant effect, and two with over-dominant effect were observed (Table 5 and Supplementary Table S6). qMIC-C02-1 with partial dominant effect was identified simultaneously in the MARBCF_1_ and MARBCF_1_MPH datasets.

#### Fiber elongation

Twenty-nine QTLs were identified on 17 chromosomes in the six datasets, explaining 3.38–23.41% of the total PV. Fourteen QTLs were identified in more than two datasets or environments (Table 4). Five QTLs were detected in the IF_2_ dataset and its MPH dataset, among which two QTLs exhibited partial dominant effect and three QTLs showed apparent over-dominant effect (Table 5 and Supplementary Table S4). In the HSBCF_1_ dataset and its MPH dataset, seven QTLs with additive effect and four with over-dominant effect were detected (Table 5 and Supplementary Table S5). In the MARBCF_1_ population, twelve QTLs with additive effect and nine with over-dominant effect were observed (Table 5 and Supplementary Table S6). Four QTLs were identified simultaneously in the MARBCF_1_ and MARBCF_1_MPH datasets.

#### Fiber strength

Twenty-seven QTLs, explaining 3.23–24.73% of the total PV, were detected using the six datasets (Table 4). In the IF_2_ population, seven QTLs were detected. In a combined analysis of the IF_2_ dataset and its MPH dataset, one with partial dominant effect and six with over-dominant effect were observed (Table 5 and Supplementary Table S4). qFS-C25-2 was detected partial dominant effect in 2014Yc and over-dominant effect in 2014Bg. In the HSBCF_1_ population, eight and two QTLs exhibited additive effect and over-dominant effect, respectively (Table 5 and Supplementary Table S5). In the MARBCF_1_ population, six QTLs with additive effect and over-dominant effect were observed, respectively (Table 5 and Supplementary Table S6). qFS-C03-1 with over-dominant effect was identified in both the MARBCF_1_ and MARBCF_1_MPH datasets. qFS-C24-1 with over-dominant effect was identified in the MARBCF_1_MPH dataset in both 2015Yc and 2015Bg, which showed favorable alleles that were conferred by different parents in these two environments.

### Multi-environment analysis of main-effect QTL and QE interactions

**Figure 2:**
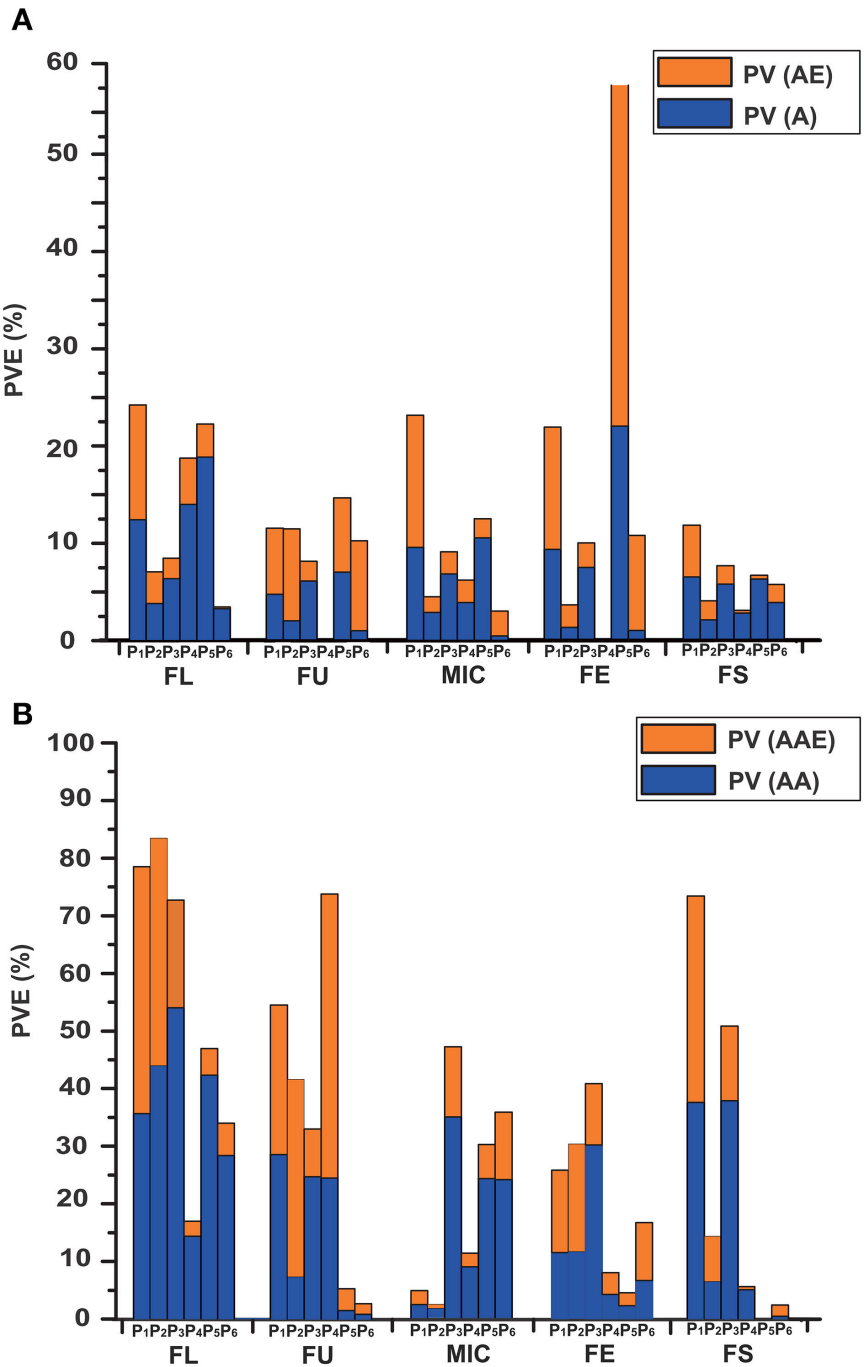
Phenotypic variance explained by the m-QTLs and e-QTLs in the IF_2_, HSBCF_1_, MARBCF_1_, IF_2_MPH, HSBCF_1_MPH, and MARBCF_1_MPH datasets for fiber quality traits. **(A)** Phenotypic variance explained by m-QTLs. PV, the phenotypic variance that the total effects explained; PV (A), the phenotypic variation that the main effects explained; PV (AE), the phenotypic variation that the environmental interaction effects explained. **(B)** Phenotypic variance explained by the e-QTLs. PV, the phenotypic variation that the total epistasis effects explained; PV (AA), the phenotypic variation that the main epistasis effects explained; PV (AAE), the phenotypic variation that the environmental interaction effects explained. P_1_, IF_2_; P_2_, HSBCF_1_; P_3_, MARBCF_1_; P_4_, IF_2_MPH; P_5_, HSBCF_1_MPH; P_6_, MARBCF_1_MPH.

In total, 104 m-QTLs and QEs for fiber quality were identified in the IF_2_, HSBCF_1_, MARBCF_1_ datasets, and their MPH datasets (Figure 2 and Supplementary Tables S7, S8).

For the IF_2_ population, 36 and 12 m-QTLs were identified in the IF_2_ and IF_2_MPH datasets, respectively. There were, on average, 7.2 m-QTLs [PV (A) = 8.41%, PV (AE) = 10.04%] for each trait identified in the IF_2_ dataset, whereas there were 2.4 m-QTLs [PV (A) = 2.31%, PV (AE) = 3.73%] in the IF_2_MPH dataset. A locus, ImaqFE-C13-1, showed significant effect with 6.81% of the total PV [PV (A) and PV (AE)] explained.

In the HSBCF_1_ population, a total of 16 and 6 m-QTLs were detected in the HSBCF_1_ and HSBCF_1_MPH datasets, respectively. In the HSBCF_1_ dataset, an average of 3.2 m-QTLs and 6.41% of the PV (A) and 2.17% PV (AE) were found. Furthermore, in the HSBCF_1_MPH dataset, the number of m-QTLs ranged from zero to four for fiber quality traits, with an average of 4.09% of the PV (A) and 1.47% PV (AE). No m-QTL was detected for FU and FE. Two m-QTLs, B_1_MmaqFL-C10-1 and B_1_MmaqMIC-C09-1, were found to have significant effects with more than 5% of the total PV explained.

In the MARBCF_1_ population, 27 and 7 m-QTLs were detected in the MARBCF_1_ and MARBCF_1_MPH datasets, respectively. On average, 5.4 m-QTLs [PV (A) = 12.86%, PV (AE) = 9.72%] were detected in the MARBCF_1_ dataset, whereas there were 1.4 m-QTLs [PV (A) = 1.81%, PV (AE) = 4.74%] in the MARBCF_1_MPH dataset. In the MARBCF_1_ dataset, two major m-QTLs, B_2_maqFE-C04-1 and B_2_maqFE-C14-1, were identified to be located within the marker intervals of i46763Gh-i10499Gh and i21369Gh-i04874Gh, with 19.16 and 16.53% of the observed PV explained, respectively. Five m-QTLs, B_2_maqFU-C14-1, B_2_maqFU-C24-1, B_2_maqFE-C06-1, B_2_maqFE-C07-1, and B_2_maqFE-C18-1, were identified to have significant effects, with more than 5% of the total PV explained. In the MARBCF_1_MPH dataset, B_2_MmaqFU-C09-1 was identified between markers i25759Gh and i03659Gh, with 10.14% of the total PV explained. Two m-QTLs, B_2_MmaqFE-C18-1 and B_2_MmaqFS-C24-1, exhibited significant effects, with more than 5% of the total PV explained.

### Epistatic QTLs detected in IF_2_ population, two BCF_1_ populations, and their MPH datasets

The e-QTLs and QEs identified in the IF_2_, HSBCF_1_, MARBCF_1_ datasets and their corresponding MPH datasets have been shown in Figure 2, Table 6, and Supplementary Tables S9, S10. In total, 70, 82, 31, 62, 38, and 31 e-QTLs pairs were identified in the IF_2_, HSBCF_1_, MARBCF_1_, IF_2_MPH, HSBCF_1_MPH, and MARBCF_1_MPH datasets, respectively. These e-QTLs explained more than 30% PV for FL, FU, and FS in the IF_2_ dataset; all fiber traits in the HSBCF_1_ dataset; FL in the MARBCF_1_ dataset; FL, FU, and FE in the IF_2_MPH dataset; FU in the HSBCF_1_MPH dataset; and FL and MIC in the MARBCF_1_MPH dataset. In addition, environmental interactions have a certain impact on the PV of these e-QTLs. On average, the QEs of e-QTLs for each trait explained 24.01, 12.41, 3.25, 20.01, 11.58, and 6.18% of the total PV in the IF_2_, HSBCF_1_, MARBCF_1_, IF_2_MPH, HSBCF_1_MPH, and MARBCF_1_MPH datasets, respectively.

**Table 6:** Type of epistatic interactions and the total phenotypic variation explained by e-QTLs detected in the IF_2_, HSBCF_1_, and MARBCF_1_ datasets and their MPH datasets.

**Population**	**Traits^a^**	**Type of epistasis^b^**			**Sum^c^**	**Total variation (%)^d^**		
		**I**	**II**	**III**		**PV^d^**	**PV(AA)^d^**	**PV(AAE)^d^**
IF_2_	FL	0	1	19	20	77.66	35.25	42.41
	FU	0	0	14	14	53.93	28.26	25.67
	MIC	0	0	1	1	4.60	2.21	2.39
	FE	0	1	17	18	25.51	11.36	14.15
	FS	0	0	17	17	72.60	37.15	35.45
HSBCF_1_	FL	0	0	24	24	71.91	53.43	18.48
	FU	0	1	11	12	32.65	24.46	8.19
	MIC	0	0	16	16	46.51	34.44	12.07
	FE	0	2	11	13	40.36	29.86	10.50
	FS	0	0	17	17	50.25	37.42	12.83
MARBCF_1_	FL	0	0	17	17	46.38	41.85	4.53
	FU	0	1	1	2	5.19	1.49	3.70
	MIC	0	1	10	11	29.72	23.87	5.85
	FE	0	0	1	1	4.42	2.23	2.19
	FS	0	0	0	0	0.00	0.00	0.00
IF_2_MPH	FL	0	0	19	19	82.59	43.47	39.12
	FU	0	0	12	12	41.22	7.25	33.97
	MIC	0	0	2	2	2.26	1.56	0.70
	FE	0	0	25	25	30.00	11.53	18.47
	FS	0	0	4	4	14.17	6.39	7.78
HSBCF_1_MPH	FL	0	0	7	7	16.74	14.15	2.59
	FU	0	0	18	18	73.01	24.24	48.77
	MIC	0	0	7	7	11.03	8.74	2.29
	FE	0	0	4	4	7.88	4.13	3.75
	FS	0	0	2	2	5.49	5.00	0.49
MARBCF_1_MPH	FL	0	0	12	12	33.61	28.04	5.57
	FU	0	0	1	1	2.61	0.81	1.80
	MIC	0	0	12	12	35.31	23.68	11.63
	FE	0	0	5	5	16.50	6.54	9.96
	FS	0	0	1	1	2.34	0.42	1.92

a FL, fiber length; FU, fiber uniformity; MIC, micronaire; FE, fiber elongation; FS, fiber strength.

b *Type of epistasis: (I) two loci with m-QTL, (II) one loci with m-QTL and the other loci without significant m-QTL and (III) two loci without significant m-QTL*.

c *Sum total number of epistatic interactions*.

d *PV, the phenotypic variation that the total epistasis effect explained; PV(AA), the phenotypic variation that the main epistasis effect explained; PV (AAE), the phenotypic variation that the environmental interaction effects explained*.

The e-QTLs were divided into three types: (I) both the loci were m-QTLs, (II) one locus was an m-QTL and the other was not, and (III) both the loci were not m-QTLs (Li et al., 2001). Of the e-QTLs detected in the IF_2_ dataset, two pairs of epistatic interactions were type II and the remaining interactions were type III. All the e-QTL interactions detected in the IF_2_MPH dataset were type III (Table 6). Of the e-QTLs in the HSBCF_1_ population, three pairs of e-QTL interactions were type II and all the remaining interactions were type III. Of the e-QTLs detected in the MARBCF_1_ population, two pairs of e-QTL interactions were type II, and all the remaining interactions occurred between two complementary loci (type III).

### Congruence analysis of the single-locus QTLs and main-effect QTLs

Comparing the additive QTLs that were identified, a total of 25 QTLs identified by the CIM method had the overlapping confidence intervals with 28 m-QTLs identified by the ICIM method, of which some single-locus QTLs harbored two m-QTLs identified in different datasets (Figure 1 and Supplementary Tables S3, S7, S8).

For FL, three stable single-locus QTLs qFL-C09-2, qFL-C14-3, and qFL-C19-3 had the same or overlapping confidence intervals with three m-QTLs B_1_maqFL-C09-1, B_2_maqFL-C14-1, and B_2_maqFL-C19-1, respectively. The confidence interval of the stable single-locus QTL qFL-C20-4 harbored two m-QTLs ImaqFL-C20-3 and B_2_maqFL-C20-1. The m-QTLs ImaqFL-C05-1, B_1_maqFL-C08-1, ImaqFL-C13-1, B_2_maqFL-C17-1, and ImaqFL-C20-1, also had overlapping confidence intervals with the QTLs qFL-C05-1, qFL-C08-1, qFL-C13-2, qFL-C17-2, and qFL-C20-3, respectively, which could only be detected in one environment.

For FU, the confidence interval of the single-locus QTLs qFU-C03-2 and qFU-C25-1 harbored two m-QTLs ImaqFU-C03-1 and IMmaqFU-C03-1 and ImaqFU-C25-1 and IMmaqFU-C25-1, respectively. The m-QTLs B_2_MmaqFU-C09-1, B_2_maqFU-C14-1, and ImaqFU-C20-1, also had overlapping confidence intervals with the QTLs qFU-C09-2, qFU-C14-2, and qFU-C20-1, respectively. All conformant single-locus QTLs could only be detected in one environment.

For MIC, two stable single-locus QTLs qMIC-C05-1 and qMIC-C14-2 had overlapping confidence intervals with two m-QTLs ImaqMIC-C05-1 and B_2_maqMIC-C14-2, respectively. The confidence interval of the stable QTL qMIC-C01-1 harbored two m-QTLs ImaqMIC-C01-2 and IMmaqMIC-C01-1. The m-QTLs ImaqMIC-C01-3, ImaqMIC-C13-1, ImaqMIC-C16-1, and ImaqMIC-C18-1 also had overlapping confidence intervals with the QTLs qMIC-C01-3, qMIC-C13-2, qMIC-C16-1, and qMIC-C18-1, respectively, which could only be detected in one environment.

For FE, the three single-locus QTLs qFE-C06-1, qFE-C13-1, and qFE-C14-3 that were detected in only one environment had overlapping confidence intervals with the m-QTLs B_2_maqFE-C06-1, ImaqFE-C13-1, and B_2_maqFE-C14-1, respectively.

For FS, only one single-locus QTL qFS-C19-1 detected in 2014Bg of the MARBCF_1_ dataset had overlapping confidence intervals with the m-QTL B_2_maqFS-C19-1.

## Discussion

### Usefulness of IF_2_ and BCF_1_ populations

Permanent populations possessing heterozygotes are a good choice for studying the genetic basis of heterosis (Hua et al., 2003; Tang et al., 2010; Guo et al., 2013, 2014; Shang et al., 2016). The two BCF_1_ populations and the IF_2_ population used in this experiment were specifically designed to achieve comprehensive dissection of heterosis. Such a design possesses several advantages. First, the genotypes of the IF_2_ and two BCF_1_ populations can be clearly deduced from the parental RILs and original parents. Second, these three populations can be repeated in exactly the same manner. Third, it provides an opportunity for analyzing heterosis by mapping HL, rather than single analyses of direct trait performance. Fourth, both IF_2_ and BCF_1_ populations have a high degree of heterozygosity. Overall, the combination of these three populations can cover more heterozygous loci and detect more QTLs than a single population.

Another characteristic is that, in the present study, CIM and ICIM were simultaneously used to detect additive QTLs. CIM not only preserves the feature of interval mapping but also controls the residual genetic variation in the rest of the genome for interval testing. However, multiple environments were regarded as multiple traits when CIM was used to identify QTLs; therefore, CIM cannot detect QTLs across multiple environments. ICIM is a modified algorithm that has all the advantages of CIM. It avoids the complicated background marker selection process and the possible increase of sampling variance in CIM. Especially for phenotypic data measured across multiple locations and/or years, the ICIM method can achieve multi-environment QTL detection with multi-environment trials (MET) function. Generally, the two QTL mapping models emphasize different considerations. The QTLs identified by CIM are those in a single environment. However, a stable QTL is considered to be detected across multiple environments, and ICIM integrates the phenotype data of various environments to detect QTLs. Therefore, some QTLs were solely detected by one of the two methods in the previous mapping results. Further studies should investigate for those stable QTLs, such as qFL-C09-2, qFL-C14-3, qFL-C19-3, qFL-C20-4, qMIC-C05-1, qMIC-C14-2, and qMIC-C01-1, detected by the CIM method, which were once again identified by the ICIM method. However, there were still some limitations. Because of the different algorithms of these two methods, fewer common QTLs were detected. The stable QTLs of FU, FE, and FS detected by CIM were not identified in ICIM. The density of the genetic map used in the present study was one of the reasons, as QTL positioning is greatly dependent on the map density. Genome-wide association study (GWAS) should be considered for positioning in future research.

### Congruence and reliability analysis of additive QTLs

In our previous studies, 47 QTLs related to fiber quality traits were identified in the RIL population developed with the same parents by CIM analysis (Li et al., 2016). In this study, 167 QTLs were detected by CIM analysis in the IF_2_, HSBCF_1_, MARBCF_1_ datasets and their MPH datasets. Among these, 19 QTLs identified previously were once again identified in the present study (Supplementary Table S3), most of which overlapped with QTLs detected in the IF_2_, HSBCF_1_, and MARBCF_1_ datasets but not in their MPH datasets. The reason for this may be that the RILs are homozygous, and only QTLs with additive effect can be identified.

Fiber quality has been identified in a number of QTL studies (Shen et al., 2005; Said et al., 2013; Tan et al., 2014; Wang et al., 2015, 2016; Li et al., 2016), most of which has been uploaded into the CottonQTLdb database (http://www.cottonqtldb.org.) developed by Said et al. (2015). Comparing the QTLs detected in the present study with those QTLs included in the database based on genetic position and physical position, of the 68 stable QTLs identified by the CIM method and the 104 additive QTLs identified by the ICIM method, 25 QTLs identified by CIM and 30 QTLs identified by ICIM were new loci for fiber quality QTLs. Among which, there were three for FL, eight for FU, six for MIC, six for FE, and two for FS in the CIM experiment; seven for FL, four for FU, nine for MIC, eight for FE, and two for FS in the ICIM experiment. All the remaining QTLs had been reported in previous studies. In addition, qFL-C17-2, qFU-C03-2, qFU-C20-1, qMIC-C13-2, and qMIC-C18-1 could be important QTLs detected in this study, as they were not only identified and confirmed by CIM and ICIM simultaneously but also by previous studies (Zhang et al., 2012; Said et al., 2015; Jamshed et al., 2016; Wang et al., 2016).

### Heterotic loci in the IF_2_ and two BCF_1_ populations

A heterotic locus is defined as a locus showing significantly different effect between the hybrid and the mean values of its parents (Hua et al., 2003); HL can be implemented using MPH dataset of the IF_2_ and two BCF_1_ populations. In this research, 24, 23, and 30 HLs for fiber quality traits were detected using the MPH datasets of IF_2_, HSBCF_1_, and MARBCF_1_ datasets with the CIM method, respectively (Supplementary Table S11), and 25 were detected with the ICIM method in these three MPH datasets (Supplementary Table S8). Some researchers have indicated that HLs are independent of the QTLs that control directly measured trait performance (Hua et al., 2003; Guo et al., 2013). However, in the present study, we found that HLs were not independent and that they overlapped with a subset of QTLs that control the *per se* performance of the hybrid. In the CIM experiment, in the IF_2_MPH dataset, nine HLs overlapped with the QTLs detected in the IF_2_ dataset, including three of FL, five of FU, and one of MIC. In the HSBCF_1_MPH dataset, 10 HLs were found to overlap with the QTLs detected in the HSBCF_1_ dataset, and eight HLs of the MARBCF_1_MPH dataset overlapped with the QTLs detected in the MARBCF_1_ dataset. In the ICIM experiment, five m-QTLs (HLs) of these three MPH datasets overlapped with those detected in the *per se* performance datasets. These results provide evidence for the above conclusion, that is, an identical genetic mode of action exists in directly measured trait performance and MPH in the upland cotton hybrid. In fact, it is difficult to demonstrate the genetic mechanism underlying fiber quality traits without referring to hybrid vigor and vice versa.

The HLs were not randomly distributed across chromosomes and chromosomal regions. Some HLs were identified as “clusters” and “hotspots,” where clusters and hotspots were defined as containing multiple HLs within ~20 cM regions for different and same traits, respectively (Guo et al., 2007; Said et al., 2013; Li et al., 2016). In the present study, seven HL clusters and two HL hotspots were found in the CIM experiment (Supplementary Table S12). Only one HL cluster was found in the ICIM experiment, owing to the fact that fewer HLs were detected in MPH datasets by ICIM method. Among the clusters, Chr21-cluster-1 must be important; it contains three HLs covering three fiber traits, and two of the three HLs were stable HLs. Chr09-cluster-1 detected by the ICIM method also include three HLs for different traits, more importantly, the stable HL qFU-C09-1, detected by CIM, is located in this cluster. Therefore, Chr09 may be a chromosome with abundant heterosis genes for fiber traits. This needs to be studied further.

Heterotic loci are sensitive to the environment (Shang et al., 2016). In the CIM experiment, only two, one, and two HLs that were detected in both years were found in the IF_2_MPH, HSBCF_1_MPH, and MARBCF_1_MPH datasets, respectively (Supplementary Table S11). Among them, qMIC-C08-1 was identified in 2 years in the IF_2_MPH dataset and 1 year in the HSBCF_1_MPH dataset, making a stable contribution to the change of MIC in these three environments. qMIC-C15-1 was detected in 2015Bg of the HSBCF_1_ dataset and 2015Yc and 2015Bg of the HSBCF_1_MPH dataset with ~10% PV of MIC. qFS-C07-1 could also be an important QTL/HL identified in the current study, which was previously reported by several researchers as a major QTL that controls FS (Zhang et al., 2009, 2012; Sun et al., 2012; Said et al., 2015; Jamshed et al., 2016), explaining 9.10 and 18.41% of the observed PVs for MPH in 2015Yc and 2015Bg of the MARBCF_1_MPH dataset, respectively. The remaining two stable HLs qFE-C02-1 and qFS-C24-1 showed inconsistent parental source of favorable alleles in different environments. This illustrated that the stability of HLs was affected by genotype and environmental interaction, which should be taken into account for hybrid breeding of upland cotton.

### Cumulative effect of the genetic basis of heterosis of fiber quality in upland cotton

In the present study, high heterosis for some traits was found. For instance, there was an average of 16.54% MPH for MIC and 9.37% for FS in the IF_2_ population, 9.58% for FS in the HSBCF_1_ population, and 12.77% for FL, 10.23% for MIC, and 9.83% for FS in the MARBCF_1_ population (Table 2). However, some traits have a low average MPH, because there are many individuals with an obviously higher or lower phenotypic value than their parents, which can be seen from the broad range of MPH (Table 2 and Supplementary Table S1). Therefore, it can be concluded that, even for heterotic hybrids, heterozygosity was not always necessarily beneficial for the phenotype of the trait; this was also proven by previous research (Li et al., 2008). Intriguingly, the traits with a low average MPH showed high heterosis in their extreme lines. This can be illustrated by an example of FE, which showed a low average MPH, but its top 10 high-heterosis hybrids had a high level of heterosis (Supplementary Table S2). Therefore, as concluded by previous research (Luo et al., 2009; Liang et al., 2015; Shang et al., 2016), a high level of heterosis arose from heterozygosity of certain loci instead of whole genome heterozygosity.

In the current analysis of MPH datasets, we could only detect the dominant effect based on the single-locus QTL mapping of the CIM method. If a QTL was detected only in the MPH dataset, but not detected in the RILs, IF_2_, and BCF_1_ datasets, we considered that the additive effect of the QTL was very small, that is, |*d*/*a*| > 1; the QTL was considered to be an over-dominant QTL, consistent with the results of previous studies (Luo et al., 2009; Shang et al., 2016). In our present research, in the IF_2_ population, 24.24% partial dominant QTLs and 69.70% over-dominant QTLs were identified. In the HSBCF_1_ population, 66.67% additive QTLs, 2.90% partial dominant QTLs, and 30.43% over-dominant QTLs were detected. In the MARBCF_1_ population, 58.90% additive QTLs, 1.37% partial dominant QTLs, and 38.36% over-dominant QTLs were detected (Table 5). These results revealed that the genetic basis of heterosis slightly varied in different populations. At the single-locus level, overdominance and partial dominance were the main contributors in the IF_2_ population, whereas additive effect and overdominance were the primary causes in the two BCF_1_ populations. This contrasts with the conclusions where dominance mainly contributed to maize heterosis in IF_2_ population (Tang et al., 2010), and partial dominance and overdominance mainly contributed to cotton heterosis in the BCF_1_ populations (Shang et al., 2016). It is not surprising that the performance of hybrids in the BCF_1_ populations was largely affected by the additive effect. In most classical quantitative studies based on backcross populations, trait performance was improved to a certain extent when subjected to selection (additive effect). Thus, in those hybrids of BCF_1_, the selection might eliminate most combined genes or hybrid breakdown genes observed in our base RILs. This could be explained by the phenomenon that was observed in our previous study that the phenotypes of some RILs exceeded those of the two parents (Li et al., 2016). Furthermore, Luo et al. (2009) investigated QTLs for yield components in two BCF_1_ populations and revealed that additive effect and overdominance were identified as the major components of heterosis in rice, which was consistent with the conclusion that additive gene action was an important cause for heterosis in the BCF_1_ population. A recently study showed that both dominance and overdominance made the key contribution to heterosis of an elite maize hybrid using an IF_2_ population (Guo et al., 2014). By mapping HLs in chromosome segment introgression lines of cotton, Guo et al. (2013) suggested that the overdominance mainly contributed to the heterosis of yield and agronomic traits. All the above results adequately proved the importance of additive, partial dominant, and overdominant effects in heterosis but with differences among the species and populations. However, the cause of overdominance is a controversial issue. The QTLs that show overdominant effects may be pseudo-overdominant (Zhou et al., 2012). The authenticity of the overdominance effect cannot be distinguished using the current genetic population design and genetic mapping method, and this is still a limitation for understanding heterosis.

By comparing the genetic effects identified in the IF_2_, HSBCF_1_, and MARBCF_1_ datasets and their MPH datasets, we were able to explore the effect of environmental interaction and the relative importance of m-QTLs and e-QTLs (Figure 2 and Table 6). The average PV of m-QTLs and e-QTLs in the IF_2_ and IF_2_MPH datasets, e-QTLs in the HSBCF_1_MPH dataset, and m-QTLs in the MARBCF_1_MPH dataset were smaller than their corresponding PV explained by QEs, which revealed that the environment was a critical factor in the expression of these m-QTLs and e-QTLs. Previous heterosis and gene action studies pointed out that there was little non-additive gene action for fiber quality traits involved in upland cotton crosses (Meredith and Bridge, 1972; Meredith, 1990). In contrast, in the present study, except for the MARBCF_1_ dataset, the total PV of e-QTLs was much larger than that of the m-QTLs for fiber quality. Notably, the total PV that the e-QTLs explain was several times the PV explained by m-QTLs in all of the MPH datasets (35.73 vs. 6.57% in the IF_2_MPH dataset, 24.97 vs. 7.97% in the HSBCF_1_MPH dataset, and 19.96 vs. 9.01% in the MARBCF_1_MPH dataset). These results indicate that epistasis plays a vital role in controlling the phenotype and heterosis of fiber quality in upland cotton. Recently, some research focusing on quantitative traits using the QTL mapping method, have strongly proved the common feature of epistasis in genetic populations (Li et al., 2001; Luo et al., 2001; Melchinger et al., 2007; Shang et al., 2016; Wang et al., 2016). By analyzing the interaction of rice yield-related traits, Li et al. (2001) and Luo et al. (2009) detected a great quantity of e-QTLs with a larger PV (42.5–59.0%) and few m-QTLs with PV only 9.6–30.4% in the RIL and BCF_1_ populations, respectively. In addition, Wang et al. (2016) identified 238 e-QTLs for fiber quality and yield traits and concluded that epistasis is very important in heterosis of the BCF_1_ populations. Furthermore, there may be three types of epistasis that influence quantitative traits (Li et al., 1998). However, in the present study, almost all of the detected interaction pairs happened between complementary loci (Table 6). This was consistent with Li et al. (2001) and Shang et al. (2016), whose studies showed that the interactions of e-QTL were more likely to occur between digenic complementary loci. The predominance of epistasis between complementary loci indicates that fiber quality trait-related e-QTLs occur more in multilocus genotypes than in specific alleles at individual loci.

Altogether, our results on heterosis indicate that, although the molecular mechanism of the genetic basis of heterosis remains unclear, it certainly refers to multiple QTLs that differ among populations with regard to estimates of the relative contributions of additive, partial dominance, overdominance, and epistasis effects. The integration of results from the single-locus and multi-environment QTL analysis indicated that overdominance and epistasis were the most important factors for fiber quality heterosis in upland cotton. The heterosis genes can be further exploited by detection of significant HLs. Further studies are required to analyze the complex molecular genetic basis that contributes to cotton fiber heterosis.

## Author contributions

CL and SZ designed the experiments and wrote the manuscript. CL and HY analyzed the data. ChL, TZ, and XD participated in field trials. TZ, YD, JH, YZ, and FZ assisted in editing the article. MD contributed to the manuscript preparation. SZ and JC conducted and supervised the experiments.

### Conflict of interest statement

The authors declare that the research was conducted in the absence of any commercial or financial relationships that could be construed as a potential conflict of interest.

## References

[B1] Birchler J. A. Yao H. Chudalayandi S. Vaiman D. Veitia R. A. (2010). Heterosis. Plant Cell 22, 2105–2112. 10.1105/tpc.110.076133 20622146 PMC2929104

[B2] Comstock R. E. Robinson H. F. (1952). Estimation of average dominance of genes, in Heterosis, ed Gowen J. W. (Ames, IA: Iowa State College Press), 495–516.

[B3] Crow J. F. (1948). Alternative hypotheses of hybrid vigor. Genetics 33, 477–487. 17247292 10.1093/genetics/33.5.477 PMC1209419

[B4] Frascaroli E. Cane M. A. Landi P. Pea G. Gianfranceschi L. Villa M.. (2007). Classical genetic and quantitative trait loci analyses of heterosis in a maize hybrid between two elite inbred lines. Genetics 176, 625–644. 10.1534/genetics.106.064493 17339211 PMC1893040

[B5] Garcia A. A. F. Wang S. Melchinger A. E. Zeng Z.-B. (2008). Quantitative trait loci mapping and the genetic basis of heterosis in maize and rice. Genetics 180, 1707–1724. 10.1534/genetics.107.082867 18791260 PMC2581970

[B6] Guo T. Yang N. Tong H. Pan Q. Yang X. Tang J. (2014). Genetic basis of grain yield heterosis in an “immortalized F(2)” maize population. Theor. Appl. Genet. 127, 2149–2158. 10.1007/s00122-014-2368-x 25104328

[B7] Guo W. Cai C. Wang C. Han Z. Song X. Wang K.. (2007). A microsatellite-based, gene-rich linkage map reveals genome structure, function and evolution in *Gossypium*. Genetics 176, 527–541. 10.1534/genetics.107.070375 17409069 PMC1893075

[B8] Guo X. Guo Y. Ma J. Wang F. Sun M. Gui L.. (2013). Mapping heterotic loci for yield and agronomic traits using chromosome segment introgression lines in cotton. J. Integr. Plant. Biol. 55, 759–774. 10.1111/jipb.12054 23570369

[B9] Hua J. Xing Y. Wu W. Xu C. Sun X. Yu S.. (2003). Single-locus heterotic effects and dominance by dominance interactions can adequately explain the genetic basis of heterosis in an elite rice hybrid. Proc. Natl. Acad. Sci. U.S.A. 100, 2574–2579. 10.1073/pnas.0437907100 12604771 PMC151382

[B10] Hua J. Xing Y. Xu C. Sun X. Yu S. Zhang Q. (2002). Genetic dissection of an elite rice hybrid revealed that heterozygotes are not always advantageous for performance. Genetics 162, 1885–1895. 12524357 10.1093/genetics/162.4.1885 PMC1462368

[B11] Hull F. H. (1945). Recurrent selection for specific combining ability in corn. J. Am. Soc. Agron. 37, 134–145. 10.2134/agronj1945.00021962003700020006x

[B12] Jamshed M. Jia F. Gong J. Palanga K. K. Shi Y. Li J.. (2016). Identification of stable quantitative trait loci (QTLs) for fiber quality traits across multiple environments in *Gossypium hirsutum* recombinant inbred line population. BMC Genomics 17:197. 10.1186/s12864-016-2560-2 26951621 PMC4782318

[B13] Jiang G. Zeng J. He Y. (2014). Analysis of quantitative trait loci affecting chlorophyll content of rice leaves in a double haploid population and two backcross populations. Gene 536, 287–295. 10.1016/j.gene.2013.12.010 24361205

[B14] Lacape J.-M. Nguyen T.-B. Courtois B. Belot J.-L. Giband M. Gourlot J.-P. (2005). QTL analysis of cotton fiber quality using multiple × backcross generations. Crop Sci. 45, 123–140. 10.2135/cropsci2005.0123a

[B15] Lariepe A. Mangin B. Jasson S. Combes V. Dumas F. Jamin P.. (2012). The genetic basis of heterosis: multiparental quantitative trait loci mapping reveals contrasted levels of apparent overdominance among traits of agronomical interest in maize (*Zea mays* L.). Genetics 190, 795–811. 10.1534/genetics.111.133447 22135356 PMC3276634

[B16] Li C. Dong Y. Zhao T. Li L. Li C. Yu E.. (2016). Genome-wide SNP linkage mapping and QTL analysis for fiber quality and yield traits in the upland cotton recombinant inbred lines population. Front. Plant Sci. 7:1356. 10.3389/fpls.2016.01356 27660632 PMC5014859

[B17] Li H. H. Ye G. Y. Wang J. K. (2007). A modified algorithm for the improvement of composite interval mapping. Genetics 175, 361–374. 10.1534/genetics.106.066811 17110476 PMC1775001

[B18] Li L. Lu K. Chen Z. Mu T. Hu Z. Li X. (2008). Dominance, overdominance and epistasis condition the heterosis in two heterotic rice hybrids. Genetics 180, 1725–1742. 10.1534/genetics.108.091942 18791236 PMC2581971

[B19] Li Z. Pinson S. R. Stansel J. W. Paterson A. H. (1998). Genetic dissection of the source-sink relationship affecting fecundity and yield in rice (shape *Oryza sativa* L.). Mol. Breed. 4, 419–426. 10.1023/A:1009608128785

[B20] Li Z. K. Luo L. J. Mei H. W. Wang D. L. Shu Q. Y. Tabien R.. (2001). Overdominant epistatic loci are the primary genetic basis of inbreeding depression and heterosis in rice. I. Biomass and grain yield. Genetics 158, 1737–1753. 10.3410/f.1002133.8203 11514459 PMC1461764

[B21] Liang Q. Shang L. Wang Y. Hua J. (2015). Partial dominance, overdominance and epistasis as the genetic basis of heterosis in upland cotton (*Gossypium hirsutum* L.). PLoS ONE 10:e0143548. 10.1371/journal.pone.0143548 26618635 PMC4664285

[B22] Liu R. Wang B. Guo W. Wang L. Zhang T. (2011). Differential gene expression and associated QTL mapping for cotton yield based on a cDNA-AFLP transcriptome map in an immortalized F_2_. Theor. Appl. Genet. 123, 439–454. 10.1007/s00122-011-1597-5 21512772

[B23] Lu H. Romero-Severson J. Bernardo R. (2003). Genetic basis of heterosis explored by simple sequence repeat markers in a random-mated maize population. Theor. Appl. Genet. 107, 494–502. 10.1007/s00122-003-1271-7 12759730

[B24] Luo L. Li Z.-K. Mei H. Shu Q. Tabien R. Zhong D.. (2001). Overdominant epistatic loci are the primary genetic basis of inbreeding depression and heterosis in rice. II. Grain yield components. Genetics 158, 1755–1771. 11514460 10.1093/genetics/158.4.1755 PMC1461757

[B25] Luo X. Fu Y. Zhang P. Wu S. Tian F. Liu J.. (2009). Additive and over-dominant effects resulting from epistatic loci are the primary genetic basis of heterosis in rice. J. Integr. Plant. Biol. 51, 393–408. 10.1111/j.1744-7909.2008.00807.x 21452591

[B26] Mei H. W. Li Z. K. Shu Q. Y. Guo L. B. Wang Y. P. Yu X. Q.. (2005). Gene actions of QTLs affecting several agronomic traits resolved in a recombinant inbred rice population and two backcross populations. Theor. Appl. Genet. 110, 649–659. 10.1007/s00122-004-1890-7 15647921

[B27] Melchinger A. E. Piepho H. P. Utz H. F. Muminovic J. Wegenast T. Torjek O.. (2007). Genetic basis of heterosis for growth-related traits in Arabidopsis investigated by testcross progenies of near-isogenic lines reveals a significant role of epistasis. Genetics 177, 1827–1837. 10.1534/genetics.107.080564 18039884 PMC2147963

[B28] Meredith W. R. (1990). Yield and fiber-quality potential for second-generation cotton hybrids. Crop Sci. 30, 1045–1048. 10.2135/cropsci1990.0011183X003000050018x

[B29] Meredith W. R. Bridge R. (1972). Heterosis and gene action in cotton, *Gossypium hirsutum* L. Crop Sci. 12, 304–310. 10.2135/cropsci1972.0011183X001200030015x

[B30] Minvielle F. (1987). Dominance is not necessary for heterosis: a two-locus model. Genet. Res. 49, 245–247. 10.1017/S0016672300027142

[B31] Qin H. Guo W. Zhang Y. M. Zhang T. (2008). QTL mapping of yield and fiber traits based on a four-way cross population in *Gossypium hirsutum* L. Theor. Appl. Genet. 117, 883–894. 10.1007/s00122-008-0828-x 18604518

[B32] Richey F. D. (1942). Mock-dominance and hybrid vigor. Science 96, 280–281. 10.1126/science.96.2490.280 17840481

[B33] Said J. I. Lin Z. Zhang X. Song M. Zhang J. (2013). A comprehensive meta QTL analysis for fiber quality, yield, yield related and morphological traits, drought tolerance, and disease resistance in tetraploid cotton. BMC Genomics 14:776. 10.1186/1471-2164-14-776 24215677 PMC3830114

[B34] Said J. I. Song M. Wang H. Lin Z. Zhang X. Fang D. D.. (2015). A comparative meta-analysis of QTL between intraspecific *Gossypium hirsutum* and interspecific *G. hirsutum x G. barbadense* populations. Mol. Genet. Genomics 290, 1003–1025. 10.1007/s00438-014-0963-9 25501533

[B35] Shang L. Liang Q. Wang Y. Zhao Y. Wang K. Hua J. (2016). Epistasis together with partial dominance, over-dominance and QTL by environment interactions contribute to yield heterosis in upland cotton. Theor. Appl. Genet. 129, 1429–1446. 10.1007/s00122-016-2714-2 27138784

[B36] Shen X. Guo W. Zhu X. Yuan Y. Yu J. Z. Kohel R. J. (2005). Molecular mapping of QTLs for fiber qualities in three diverse lines in Upland cotton using SSR markers. Mol. Breed. 15, 169–181. 10.1007/s11032-004-4731-0

[B37] Shull G. H. (1914). Duplicate genes for capsule-form in Bursa bursa-pastoris. Mol. Gen. Genet. 12:97–149. 10.1007/BF01837282

[B38] Stuber C. W. Lincoln S. E. Wolff D. Helentjaris T. Lander E. (1992). Identification of genetic factors contributing to heterosis in a hybrid from two elite maize inbred lines using molecular markers. Genetics 132, 823–839. 1468633 10.1093/genetics/132.3.823 PMC1205218

[B39] Sun F.-D. Zhang J.-H. Wang S.-F. Gong W.-K. Shi Y.-Z. Liu A.-Y. (2012). QTL mapping for fiber quality traits across multiple generations and environments in upland cotton. Mol. Breed. 30, 569–582. 10.1007/s11032-011-9645-z

[B40] Tan Z. Fang X. Tang S. Zhang J. Liu D. Teng Z. (2014). Genetic map and QTL controlling fiber quality traits in upland cotton (*Gossypium hirsutum* L.). Euphytica 203, 615–628. 10.1007/s10681-014-1288-9

[B41] Tang B. Jenkins J. N. McCarty J. Watson C. (1993). F_2_ hybrids of host plant germplasm and cotton cultivars: II. Heterosis and combining ability for fiber properties. Crop Sci. 33, 706–710. 10.2135/cropsci1993.0011183X003300040013x

[B42] Tang J. Yan J. Ma X. Teng W. Wu W. Dai J.. (2010). Dissection of the genetic basis of heterosis in an elite maize hybrid by QTL mapping in an immortalized F_2_ population. Theor. Appl. Genet. 120, 333–340. 10.1007/s00122-009-1213-0 19936698

[B43] Voorrips R. (2002). MapChart: software for the graphical presentation of linkage maps and QTLs. J. Hered. 93, 77–78. 10.1093/jhered/93.1.77 12011185

[B44] Wang H. Huang C. Guo H. Li X. Zhao W. Dai B.. (2015). QTL mapping for fiber and yield traits in upland cotton under multiple environments. PLoS ONE 10:e0130742. 10.1371/journal.pone.0130742 26110526 PMC4481505

[B45] Wang H. Huang C. Zhao W. Dai B. Shen C. Zhang B.. (2016). Identification of QTL for fiber quality and yield traits using two immortalized backcross populations in upland cotton. PLoS ONE 11:e0166970. 10.1371/journal.pone.0166970 27907098 PMC5131980

[B46] Wang S. Basten C. Zeng Z. (2012). Windows QTL Cartographer 2.5. Department of Statistics, North Carolina State University, Raleigh, NC.

[B47] Williams W. (1959). Heterosis and the genetics of complex characters. Nature 184, 527–530. 10.1038/184527a0 13844942

[B48] Wu J. Gutierrez O. A. Jenkins J. N. McCarty J. C. Zhu J. (2008). Quantitative analysis and QTL mapping for agronomic and fiber traits in an RI population of upland cotton. Euphytica 165, 231–245. 10.1007/s10681-008-9748-8

[B49] Xiao J. H. Li J. M. Yuan L. P. Tanksley S. D. (1995). Dominance is the major genetic-basis of heterosis in rice as revealed by Qtl analysis using molecular markers. Genetics 140, 745–754. 7498751 10.1093/genetics/140.2.745 PMC1206649

[B50] Yu J. Yu S. Gore M. Wu M. Zhai H. Li X. (2013). Identification of quantitative trait loci across interspecific F_2_, F_2:3_ and testcross populations for agronomic and fiber traits in tetraploid cotton. Euphytica 191, 375–389. 10.1007/s10681-013-0875-5

[B51] Yu S. Li J. Xu C. Tan Y. Gao Y. Li X.. (1997). Importance of epistasis as the genetic basis of heterosis in an elite rice hybrid. Proc. Natl. Acad. Sci. U.S.A. 94, 9226–9231. 10.1073/pnas.94.17.9226 11038567 PMC23127

[B52] Zhang K. Zhang J. Ma J. Tang S. Liu D. Teng Z. (2012). Genetic mapping and quantitative trait locus analysis of fiber quality traits using a three-parent composite population in upland cotton (*Gossypium hirsutum* L.). Mol. Breed. 29, 335–348. 10.1007/s11032-011-9549-y

[B53] Zhang S. Wang T. Liu Q. Gao X. Zhu X. Zhang T. (2015). Quantitative trait locus analysis of boll-related traits in an intraspecific population of *Gossypium hirsutum*. Euphytica 203, 121–144. 10.1007/s10681-014-1281-3

[B54] Zhang Z. Hu M. Zhang J. Liu D. Zheng J. Zhang K. (2009). Construction of a comprehensive PCR based marker linkage map and QTL mapping for fiber quality traits in upland cotton (*Gossypium hirsutum* L.). Mol. Breed. 24, 49–61. 10.1007/s11032-009-9271-1

[B55] Zhang Z. Shang H. Shi Y. Huang L. Li J. Ge Q.. (2016). Construction of a high-density genetic map by specific locus amplified fragment sequencing (SLAF-seq) and its application to Quantitative Trait Loci (QTL) analysis for boll weight in upland cotton (*Gossypium hirsutum*). BMC Plant Biol. 16:79. 10.1186/s12870-016-0741-4 27067834 PMC4827241

[B56] Zhou G. Chen Y. Yao W. Zhang C. Xie W. Hua J.. (2012). Genetic composition of yield heterosis in an elite rice hybrid. Proc. Natl. Acad. Sci. U.S.A. 109, 15847–15852. 10.1073/pnas.1214141109 23019369 PMC3465387

[B57] Zhu J. (1989). Estimation of Genetic Variance Components in the General Mixed Model. Ph.D. dissertation, North Carolina State University, Raleigh, NC.

