# Supplementary material for: QTL Mapping and Heterosis Analysis for Fiber Quality Traits Across Multiple Genetic Populations and Environments in Upland Cotton
Source: Front Plant Sci. 2018 Oct 15;9:1364. doi: 10.3389/fpls.2018.01364 (PMC6196769; doi:10.3389/fpls.2018.01364)
Supplement: Supplementary file 1 [file Data_Sheet_1.PDF]

Table S1 Distribution of MPH value for fiber quality traits in IF_2MPH, $HSBCF_1$MPH, and $MARBCF_1$MPH datasets of upland cotton

Traits[a]	Environment[b]	IF_2MPH dataset			$HSBCF_1$MPH dataset			$MARBCF_1$MPH dataset		
		Mean	Min	Max	Mean	Min	Max	Mean	Min	Max
FL	2014Yc	2.10	-2.90	4.06	3.04	-1.54	3.51	3.23	-1.23	4.60
	2014Bg	1.25	-2.53	3.39	2.17	-2.44	3.84	1.76	-1.90	2.79
	2015Yc	1.30	-2.28	2.95	2.76	-1.98	3.84	2.99	-1.48	4.00
	2015Bg	1.09	-3.45	2.75	2.59	-2.58	3.84	2.18	-1.83	3.65
FU	2014Yc	1.59	-2.43	4.65	2.07	-1.88	3.75	2.66	-2.55	4.85
	2014Bg	1.32	-3.50	4.65	1.48	-2.7	3.98	1.47	-2.80	3.38
	2015Yc	1.28	-3.08	3.63	1.19	-5.38	3.98	1.09	-4.53	3.80
	2015Bg	1.20	-4.45	3.83	0.68	-9.95	3.98	1.28	-7.08	3.93
MIC	2014Yc	0.53	-1.53	1.25	0.45	-0.75	1.12	0.68	-0.38	1.38
	2014Bg	0.49	-1.53	1.75	0.41	-0.97	1.20	0.39	-0.74	1.19
	2015Yc	0.12	-1.30	1.00	0.26	-1.43	0.86	0.29	-1.55	1.16
	2015Bg	0.33	-1.70	1.60	0.29	-1.30	0.86	0.32	-1.28	1.43
FE	2014Yc	-0.08	-2.38	2.63	-0.53	-3.28	3.15	-0.50	-2.95	2.15
	2014Bg	0.38	-2.70	4.00	-0.26	-1.85	3.40	-0.29	-2.38	2.53
	2015Yc	0.05	-0.28	3.28	-0.05	-1.05	2.98	-0.10	-2.45	3.40
	2015Bg	-0.01	-0.50	3.30	-0.04	-1.05	2.98	-0.08	-2.45	3.40
FS	2014Yc	3.73	-5.98	7.48	4.11	-3.20	7.68	3.84	-2.23	8.08
	2014Bg	2.86	-5.98	7.21	3.13	-3.63	7.69	3.03	-3.27	7.76
	2015Yc	2.23	-4.00	6.68	1.09	-6.15	6.50	2.51	-3.75	7.18
	2015Bg	2.47	-7.25	6.83	2.51	-7.55	5.75	2.88	-6.25	7.05

[a] FL: fiber length; FU: fiber uniformity; MIC: micronaire; FE: fiber elongation; FS: fiber strength

[b] 2014Yc: Yacheng of Hainan Province in 2014; 2014Bg: Baogang of Hainan Province in 2014; 2015Yc: Yacheng of Hainan Province in 2015; 2015Bg: Baogang of Hainan Province in 2015
