# Supplementary material for: QTL Mapping and Heterosis Analysis for Fiber Quality Traits Across Multiple Genetic Populations and Environments in Upland Cotton
Source: Front Plant Sci. 2018 Oct 15;9:1364. doi: 10.3389/fpls.2018.01364 (PMC6196769; doi:10.3389/fpls.2018.01364)
Supplement: Supplementary file 2 [file Data_Sheet_2.PDF]

Table S2 Average MPH (%) of top 10 high-heterosis hybrids of fiber quality traits

Traits [a]	Env. [b]	top 10 high-heterosis hybrids		
		IF2 (%)	HSBCF_1 (%)	MARBCF_1 (%)
FL	2014Yc	11.20	10.54	12.67
	2014Bg	16.96	17.65	17.88
	2015Yc	17.91	19.79	19.89
	2015Bg	17.25	18.51	20.00
FU	2014Yc	14.78	13.53	14.89
	2014Bg	13.28	12.76	13.30
	2015Yc	13.00	11.47	13.18
	2015Bg	13.54	11.56	13.66
MIC	2014Yc	31.38	17.28	29.42
	2014Bg	41.30	29.51	29.26
	2015Yc	22.86	12.04	16.05
	2015Bg	25.89	14.55	26.23
FE	2014Yc	16.06	4.48	4.34
	2014Bg	46.04	19.90	21.25
	2015Yc	36.10	22.41	33.21
	2015Bg	30.91	37.72	37.71
FS	2014Yc	22.34	22.60	25.18
	2014Bg	18.28	19.80	19.15
	2015Yc	12.07	13.98	16.05
	2015Bg	16.87	15.25	18.12

[a] FL: fiber length; FU: fiber uniformity; MIC: micronaire; FE: fiber elongation; FS: fiber strength

[b] 2014Yc: Yacheng of Hainan Province in 2014; 2014Bg: Baogang of Hainan Province in 2014;2015Yc: Yacheng of Hainan Province in 2015; 2015Bg: Baogang of Hainan Province in 2015
