# Supplementary material for: QTL Mapping and Heterosis Analysis for Fiber Quality Traits Across Multiple Genetic Populations and Environments in Upland Cotton
Source: Front Plant Sci. 2018 Oct 15;9:1364. doi: 10.3389/fpls.2018.01364 (PMC6196769; doi:10.3389/fpls.2018.01364)
Supplement: Supplementary file 3 [file Data_Sheet_3.PDF]

Table S3 QTLs identified for fiber quality traits in IF_2, $HSBCF_1$, $MARBCF_1$ and their MPH datasets by CIM method

Trait[a]	QTL[b]	Env.[c]	Flanking markers	Position[d]	LOD[e]	A[f]	D[f]	A+D[f]	R^2 (%)[g]	Population
FL	qFL-C02-1	2014Bg	i43421Gh-i24299Gh	41.21	5.00		-2.15		19.31	$MARBCF_1$MPH
		2015Bg	i14776Gh-i16398Gh	43.91	2.59		-2.19		12.20	IF_2MPH
	qFL-C02-2	2015Bg	i02276Gh-i01044Gh	75.71	2.80		0.56		6.28	IF_2MPH
		2015Yc	i49488Gh-i14841Gh	78.11	3.03		0.18		9.51	$HSBCF_1$MPH
	qFL-C03-1	2014Bg	i42939Gh-i05394Gh	94.91	2.54	0.02	-0.77		3.76	IF_2
	qFL-C04-1	2014Bg	i50068Gb-i43091Gh	3.01	4.35	-0.27	-1.03		7.63	IF_2
	**qFL-C05-1**	2014Yc	i26290Gh-i09437Gh	13.01	5.00	2.68	-3.14		19.72	IF_2
	**qFL-C05-2**	2015Bg	i16671Gh-i29825Gh	43.21	3.39			0.04	7.96	$HSBCF_1$
		2014Bg	i19536Gh-i34270Gh	46.91	2.59			0.03	5.90	$MARBCF_1$
	**qFL-C05-3**	2014Yc	i16666Gh-i51323Gb	50.81	3.37		0.76		13.32	$HSBCF_1$MPH
		2015Bg	i16666Gh-i22016Gh	50.81	4.60			0.24	11.52	$HSBCF_1$
		2014Yc	i09095Gh-i46446Gh	51.61	3.48			1.25	9.71	$HSBCF_1$
	qFL-C06-1	2015Bg	i21566Gh-i14061Gh	25.61	2.65		0.49		14.64	IF_2MPH
		2014Bg	i06526Gh-i34827Gh	27.81	2.93		-2.47		10.60	$MARBCF_1$MPH
		2014Yc	i06526Gh-i34827Gh	27.81	2.54	2.78	-2.86		20.89	IF_2
	qFL-C06-2	2015Yc	i37862Gh-i06396Gh	49.21	2.84			1.08	4.16	$MARBCF_1$
	qFL-C07-1	2014Yc	i01651Gh-i01602Gh	49.81	3.21	2.77	-3.00		17.97	IF_2
	qFL-C08-1	2014Yc	i04572Gh-i25482Gh	16.71	2.70	2.91	-2.70		16.55	IF_2
	qFL-C09-1	2014Bg	i40336Gh-i07864Gh	12.91	2.57			-0.17	14.84	$HSBCF_1$
	qFL-C09-2	2015Bg	i23159Gh-i47527Gh	32.71	4.78	0.48	-0.14		11.72	IF_2
		2015Yc	i23159Gh-i47527Gh	32.71	3.90	0.36	-0.39		9.54	IF_2
	qFL-C09-3	2015Bg	i36372Gh-i32479Gh	46.51	5.46			0.86	12.08	$HSBCF_1$
	qFL-C10-1	2014Yc	i00311Gh-i40792Gh	36.81	4.05	2.77	-3.00		10.48	IF_2
	qFL-C11-1	2015Yc	i50657Gb-i07729Gh	4.81	2.55			-1.73	4.59	$HSBCF_1$

qFL-C13-1	2014Yc	i13332Gh-i22760Gh	4.91	3.64	2.87	-2.64		18.32	IF_2
qFL-C13-2	2015Yc	i27668Gh-i36415Gh	30.61	3.99			0.56	10.08	$HSBCF_1$
qFL-C13-3	2015Yc	i50955Gb-i49771Gh	42.51	2.84			-0.22	7.40	$MARBCF_1$
qFL-C14-1	2014Bg	i05482Gh-i18840Gh	4.01	4.21		-2.61		19.34	$MARBCF_1$MPH
	2014Yc	i05482Gh-i04837Gh	4.01	4.19	2.85	-2.75		16.87	IF_2
	2015Bg	i22641Gh-i48509Gh	6.31	3.25	0.26	1.77		3.97	IF_2
**qFL-C14-2**	2015Bg	i23013Gh-i15343Gh	13.81	3.75			-0.82	6.33	$MARBCF_1$
**qFL-C14-3**	2015Yc	i15343Gh-i15345Gh	18.41	2.69		-0.30		7.93	$MARBCF_1$MPH
	2014Yc	i18849Gh-i00465Gh	20.91	3.02			-0.19	8.31	$MARBCF_1$
	2015Bg	i15345Gh-i00465Gh	20.91	5.39			-1.09	4.99	$MARBCF_1$
**qFL-C14-4**	2015Bg	i15340Gh-i34657Gh	23.21	3.84			-0.83	3.14	$HSBCF_1$
	2015Bg	i34657Gh-i40518Gh	24.71	5.25			-1.18	3.01	$MARBCF_1$
	2014Yc	i05040Gh-i31578Gh	28.21	4.93			-0.66	10.42	$MARBCF_1$
qFL-C16-1	2014Yc	i18208Gh-i13939Gh	6.21	3.07			0.84	16.38	$HSBCF_1$
qFL-C16-2	2015Yc	i21384Gh-i42534Gh	57.01	3.28	-0.32	0.15		7.12	IF_2
	2015Bg	i21384Gh-i31338Gh	57.21	2.68	-0.10	0.71		3.22	IF_2
	2015Bg	i21384Gh-i42534Gh	57.21	4.20		0.79		6.93	IF_2MPH
qFL-C17-1	2014Bg	i03509Gh-i14513Gh	31.31	2.97			-0.08	8.61	$HSBCF_1$
	2014Yc	i03218Gh-i03508Gh	35.11	2.79	2.74	-2.99		17.58	IF_2
qFL-C17-2	2014Bg	i18575Gh-i03527Gh	42.31	2.79			0.15	6.23	$MARBCF_1$
qFL-C18-1	2014Bg	i31525Gh-i26380Gh	58.61	4.72		-2.30		20.92	$MARBCF_1$MPH
	2014Bg	i48138Gh-i13492Gh	59.01	3.25		-2.68		10.35	$HSBCF_1$MPH
qFL-C19-1	2014Yc	i28797Gh-i50906Gb	16.01	5.48			0.51	11.35	$HSBCF_1$
	2014Yc	i28797Gh-i50906Gb	16.01	4.91		0.80		10.36	$HSBCF_1$MPH
**qFL-C19-2**	2015Yc	i09219Gh-i09217Gh	20.81	3.71			0.38	9.53	$MARBCF_1$
qFL-C19-3	2014Yc	i55376Gb-i37157Gh	26.51	2.52			-0.73	7.64	$HSBCF_1$

		2015Yc	i47122Gh-i08962Gh	27.61	5.76			2.15	12.42	MARBCF$_1$
	qFL-C20-1	2015Yc	i17417Gh-i11531Gh	4.81	3.41	-0.34	-0.38		3.00	IF$_2$
	qFL-C20-2	2014Bg	i11727Gh-i39228Gh	8.01	2.76		-0.61		4.99	IF$_2$MPH
		2015Bg	i11723Gh-i34769Gh	8.01	3.82	-0.81	-0.28		10.13	IF$_2$
		2015Yc	i11723Gh-i34769Gh	9.91	5.82	-0.45	-0.39		5.69	IF$_2$
	qFL-C20-3	2015Bg	i11611Gh-i11698Gh	13.11	3.97	-0.51	-0.13		8.29	IF$_2$
	qFL-C20-4	2014Bg	i47006Gh-i17500Gh	41.51	4.07			0.33	7.94	MARBCF$_1$
		2015Yc	i26441Gh-i17500Gh	41.51	3.29			0.12	8.81	MARBCF$_1$
		2014Yc	i17505Gh-i47439Gh	42.11	2.51			-0.31	6.26	HSBCF$_1$
		2015Bg	i17505Gh-i47439Gh	44.11	3.10			-0.45	9.26	HSBCF$_1$
	qFL-C21-1	2014Bg	i16082Gh-i00284Gh	44.61	3.26		-0.76		3.82	HSBCF$_1$MPH
	qFL-C22-1	2014Yc	i00125Gh-i30763Gh	7.51	2.52		0.86		14.63	MARBCF$_1$MPH
	qFL-C22-2	2014Bg	i22757Gh-i12927Gh	16.71	5.00		-2.24		13.08	MARBCF$_1$MPH
	qFL-C22-3	2014Bg	i12927Gh-i33209Gh	20.11	2.78		0.37		7.12	IF$_2$MPH
	qFL-C22-4	2014Bg	i12906Gh-i12725Gh	30.21	2.94		0.39		6.56	IF$_2$MPH
	qFL-C24-1	2015Bg	i04105Gh-i18808Gh	5.41	4.86		-1.27		14.48	MARBCF$_1$MPH
	qFL-C24-2	2015Yc	i37272Gh-i04012Gh	29.41	2.59	-0.29	-0.22		3.76	IF$_2$
FU	qFU-C01-1	2015Yc	i23213Gh-i36727Gh	30.31	3.79			0.89	5.10	HSBCF$_1$
		2014Yc	i42430Gh-i55243Gb	35.71	4.26	1.43	-1.45		13.61	IF$_2$
	qFU-C01-2	2014Bg	i31455Gh-i02245Gh	41.71	2.52			0.50	5.43	HSBCF$_1$
		2015Bg	i02245Gh-i02457Gh	45.71	7.09			-4.99	15.22	HSBCF$_1$
		2015Bg	i02245Gh-i02457Gh	45.71	6.83		-7.40		15.20	HSBCF$_1$MPH
	qFU-C01-3	2015Bg	i23077Gh-i32637Gh	50.41	2.67			-1.09	9.69	HSBCF$_1$
	qFU-C02-1	2015Bg	i02758Gh-i02723Gh	44.81	5.77			-4.44	8.27	HSBCF$_1$
		2015Bg	i02758Gh-i02723Gh	44.81	3.75		-6.04		8.84	HSBCF$_1$MPH
	qFU-C02-2	2014Yc	i49496Gh-i02563Gh	48.31	3.40			-0.27	16.38	MARBCF$_1$

qFU-C03-1	2015Bg	i35903Gh-i39896Gh	50.51	2.99	-0.76	0.64		19.03	IF$_2$
	2015Bg	i35903Gh-i39896Gh	52.51	3.17		0.48		14.96	IF$_2$MPH
qFU-C03-2	2014Bg	i39896Gh-i33635Gh	69.11	3.11		-0.66		6.05	IF$_2$MPH
qFU-C05-1	2015Bg	i12470Gh-i45534Gh	40.01	3.62	1.06	1.77		6.18	IF$_2$
**qFU-C05-2**	2015Bg	i19536Gh-i34270Gh	46.91	3.98	-0.33	0.65		9.57	IF$_2$
	2015Bg	i19536Gh-i34270Gh	46.91	2.72		0.64		5.57	IF$_2$MPH
qFU-C06-1	2015Bg	i26917Gh-i14061Gh	26.61	2.92	1.16	1.76		3.15	IF$_2$
qFU-C06-2	2015Bg	i06036Gh-i06037Gh	35.01	11.44			0.29	18.73	HSBCF$_1$
	2015Yc	i06036Gh-i23722Gh	36.01	2.84	-0.40	1.06		9.36	IF$_2$
	2015Yc	i06037Gh-i06505Gh	36.01	3.91		-0.90		3.89	HSBCF$_1$MPH
	2015Yc	i06037Gh-i23722Gh	36.01	3.13		1.12		3.70	IF$_2$MPH
qFU-C06-3	2015Bg	i23722Gh-i06396Gh	47.21	8.43			0.19	19.69	HSBCF$_1$
	2014Yc	i37862Gh-i06396Gh	49.21	3.15			0.70	8.63	MARBCF$_1$
**qFU-C09-1**	2014Bg	i07864Gh-i50078Gb	17.81	93.26		27.70		17.47	HSBCF$_1$MPH
qFU-C09-2	2015Bg	i05664Gh-i40760Gh	25.01	9.03			9.04	11.75	HSBCF$_1$
qFU-C09-3	2015Bg	i35007Gh-i00142Gh	30.31	2.60	-3.23	3.18		15.17	IF$_2$
**qFU-C09-4**	2014Yc	i41596Gh-i35858Gh	49.61	4.85			1.35	14.97	HSBCF$_1$
	2015Bg	i07773Gh-i15768Gh	53.01	10.82			9.05	3.00	HSBCF$_1$
	2014Bg	i18484Gh-i03595Gh	54.71	2.70	0.30	0.08		5.16	IF$_2$
qFU-C11-1	2015Bg	i33855Gh-i43823Gh	5.31	3.48	-1.99	3.20		9.25	IF$_2$
	2015Bg	i33855Gh-i43823Gh	5.31	3.69		3.44		10.43	IF$_2$MPH
qFU-C13-1	2014Yc	i23966Gh-i29670Gh	29.11	2.60			2.32	3.68	MARBCF$_1$
	2014Bg	i24929Gh-i27668Gh	30.21	3.25	-0.38	0.07		8.51	IF$_2$
	2014Bg	i32083Gh-i62433Gt	32.41	2.69			0.31	7.21	HSBCF$_1$
qFU-C14-1	2015Bg	i15536Gh-i05487Gh	1.11	3.46	-3.22	3.31		11.65	IF$_2$
	2015Bg	i15536Gh-i05487Gh	1.11	3.74		4.14		11.10	IF$_2$MPH

qFU-C14-2	2015Yc	i35279Gh-i41064Gh	37.41	2.67			0.16	16.67	HSBCF$_1$
qFU-C16-1	2015Bg	i54704Gb-i01693Gh	72.91	10.20			-0.04	21.83	HSBCF$_1$
	2015Bg	i54704Gb-i01693Gh	72.91	9.97		4.93		19.23	HSBCF$_1$MPH
qFU-C17-1	2014Bg	i14812Gh-i00652Gh	27.91	2.89			-0.19	8.87	HSBCF$_1$
qFU-C18-1	2015Bg	i13283Gh-i36753Gh	57.11	7.85			-9.44	13.87	HSBCF$_1$
qFU-C19-1	2014Bg	i22528Gh-i09637Gh	37.41	3.49			1.04	10.53	HSBCF$_1$
qFU-C20-1	2014Yc	i11735Gh-i42616Gh	31.11	5.43	0.41	-1.11		5.83	IF$_2$
qFU-C20-2	2015Bg	i11714Gh-i37554Gh	38.01	11.32			-0.09	11.59	HSBCF$_1$
	2015Bg	i11714Gh-i37554Gh	38.01	11.31		4.69		11.01	HSBCF$_1$MPH
	2014Yc	i11912Gh-i47439Gh	41.51	5.63	-0.34	-0.38		7.75	IF$_2$
qFU-C20-3	2015Yc	i17505Gh-i47439Gh	49.11	3.14		-0.70		11.41	HSBCF$_1$MPH
qFU-C20-4	2015Bg	i18012Gh-i11915Gh	57.81	3.77			-3.54	9.36	HSBCF$_1$
	2015Bg	i18012Gh-i11478Gh	57.81	3.68		-5.39		10.76	HSBCF$_1$MPH
qFU-C21-1	2015Bg	i16082Gh-i00284Gh	44.61	2.86		-1.43		13.42	MARBCF$_1$MPH
	2015Yc	i16082Gh-i00284Gh	44.61	2.69		0.01		6.98	IF$_2$MPH
qFU-C21-2	2015Yc	i41432Gh-i22642Gh	58.91	3.07		-0.10		7.32	IF$_2$MPH
	2015Yc	i07219Gh-i41613Gh	59.91	3.10		0.63		3.58	MARBCF$_1$MPH
qFU-C22-1	2015Yc	i12929Gh-i12539Gh	20.81	2.68			-0.72	3.44	HSBCF$_1$
qFU-C22-2	2015Yc	i12581Gh-i17853Gh	29.31	3.93	-0.19	0.50		8.54	IF$_2$
qFU-C22-3	2014Yc	i12810Gh-i17711Gh	40.31	3.36			-0.57	14.15	MARBCF$_1$
qFU-C23-1	2015Bg	i06287Gh-i06171Gh	0.01	5.57			-1.10	9.23	MARBCF$_1$
	2015Bg	i06287Gh-i06171Gh	0.01	3.26		-1.32		5.23	MARBCF$_1$MPH
qFU-C24-1	2014Bg	i41753Gh-i45941Gh	19.91	3.88	0.32	-0.33		9.85	IF$_2$
qFU-C25-1	2015Yc	i22495Gh-i11267Gh	41.91	2.72	-0.45	1.04		5.88	IF$_2$
qFU-C25-2	2014Bg	i40453Gh-i17145Gh	51.81	3.66	-0.34	0.16		8.74	IF$_2$
qFU-C26-1	2015Bg	i00879Gh-i32452Gh	1.01	3.75		-0.59		10.03	MARBCF$_1$MPH

		2015Yc	i00879Gh-i33827Gh	2.51	3.32		0.39		10.82	$HSBCF_1MPH$
	qFU-C26-2	2014Bg	i08565Gh-i36067Gh	35.01	3.37			0.42	8.28	$MARBCF_1$
		2014Yc	i08565Gh-i08578Gh	38.31	3.64			0.32	10.01	$HSBCF_1$
MIC	qMIC-C01-1	2015Bg	i60883Gt-i48104Gh	6.21	2.69			-0.22	3.09	$MARBCF_1$
		2015Bg	i31143Gh-i21823Gh	7.51	2.68	0.05	-0.21		3.19	IF_2
	**qMIC-C01-2**	2014Yc	i02201Gh-i32863Gh	15.51	2.57	0.13	-0.24		3.52	IF_2
	qMIC-C01-3	2015Yc	i30614Gh-i02994Gh	24.91	4.54		0.57		3.60	IF_2MPH
	qMIC-C01-4	2015Yc	i02245Gh-i02767Gh	45.71	3.28	-0.30	-0.26		3.86	IF_2
		2015Yc	i02245Gh-i44115Gh	45.71	3.74		-0.32		3.88	IF_2MPH
	qMIC-C02-1	2015Yc	i16954Gh-i20804Gh	32.21	3.14			0.97	4.20	$HSBCF_1$
		2015Yc	i18644Gh-i27649Gh	35.81	3.98			0.73	4.24	$MARBCF_1$
		2015Yc	i18644Gh-i27649Gh	35.81	4.17		0.33		4.35	$MARBCF_1MPH$
	qMIC-C02-2	2015Yc	i16398Gh-i02723Gh	44.51	2.80			-0.74	4.36	$MARBCF_1$
	qMIC-C05-1	2015Bg	i09071Gh-i01144Gh	44.11	2.69			0.42	4.46	$MARBCF_1$
		2014Yc	i53822Gb-i01144Gh	44.41	3.53			0.07	4.55	$MARBCF_1$
		2014Bg	i00341Gh-i31875Gh	47.21	3.39			0.08	4.66	$MARBCF_1$
		2014Bg	i31875Gh-i16666Gh	48.31	2.66			0.53	4.78	$HSBCF_1$
	qMIC-C05-2	2014Bg	i52021Gb-i09236Gh	60.51	2.73		0.03		4.97	$MARBCF_1MPH$
	qMIC-C08-1	2015Yc	i30195Gh-i04557Gh	35.61	3.02		-0.70		5.78	IF_2MPH
		2015Bg	i30195Gh-i04565Gh	35.91	3.11		0.68		5.88	$HSBCF_1MPH$
		2014Yc	i40070Gh-i01126Gh	38.01	2.91		-0.58		5.89	IF_2MPH
	qMIC-C09-1	2015Yc	i44757Gh-i08331Gh	18.11	3.15			0.24	6.09	$HSBCF_1$
	qMIC-C10-1	2014Yc	i11502Gh-i33011Gh	43.71	2.86			0.33	6.16	$MARBCF_1$
	qMIC-C11-1	2015Yc	i07468Gh-i36064Gh	17.11	2.97			0.41	6.25	$HSBCF_1$
		2015Yc	i07468Gh-i36064Gh	18.11	3.96		0.01		6.29	$HSBCF_1MPH$
	qMIC-C13-1	2015Yc	i37074Gh-i45163Gh	7.41	3.04			0.29	6.37	$MARBCF_1$

qMIC-C13-2	2015Bg	i62433Gt-i41278Gh	33.81	3.23	-0.12	0.10		6.80	IF$_2$
qMIC-C13-3	2015Bg	i42046Gh-i38620Gh	40.91	3.39	-0.12	0.12		6.81	IF$_2$
	2014Yc	i49771Gh-i35111Gh	44.11	2.94			0.08	7.03	HSBCF$_1$
qMIC-C14-1	2015Yc	i05482Gh-i18840Gh	4.01	3.30			0.74	7.33	HSBCF$_1$
**qMIC-C14-2**	2014Yc	i05007Gh-i40518Gh	25.71	2.68			0.13	7.56	MARBCF$_1$
	2014Yc	i15375Gh-i40518Gh	27.21	2.91			0.21	7.83	HSBCF$_1$
	2015Bg	i44975Gh-i34413Gh	30.51	4.21			0.27	7.99	MARBCF$_1$
	2015Bg	i43013Gh-i44046Gh	30.91	2.85			0.01	8.13	HSBCF$_1$
	2014Yc	i66845Ga-i05256Gh	31.21	2.66	0.07	-0.22		8.17	IF$_2$
	2015Yc	i23352Gh-i39672Gh	32.71	2.78			0.30	8.34	HSBCF$_1$
qMIC-C14-3	2015Bg	i22394Gh-i41891Gh	40.21	2.74			0.19	8.62	MARBCF$_1$
	2015Yc	i28729Gh-i00245Gh	41.01	2.92			0.10	8.77	MARBCF$_1$
	2015Yc	i23762Gh-i38809Gh	43.81	3.65			-0.01	9.73	HSBCF$_1$
qMIC-C15-1	2015Bg	i29719Gh-i49465Gh	20.51	5.66			0.27	9.83	HSBCF$_1$
	2015Bg	i29719Gh-i49465Gh	20.51	5.05		0.07		9.85	HSBCF$_1$MPH
	2015Yc	i29719Gh-i49465Gh	20.51	3.30		0.07		10.16	HSBCF$_1$MPH
qMIC-C15-2	2015Yc	i02486Gh-i22421Gh	32.81	3.42		0.53		10.58	IF$_2$MPH
qMIC-C16-1	2015Bg	i18208Gh-i14406Gh	8.71	2.61	0.14	-0.16		11.12	IF$_2$
**qMIC-C16-2**	2015Bg	i34919Gh-i45501Gh	48.91	2.56			0.46	11.93	MARBCF$_1$
	2014Bg	i46435Gh-i00787Gh	51.61	3.31			0.13	11.93	MARBCF$_1$
**qMIC-C16-3**	2014Bg	i27803Gh-i54704Gb	61.31	3.33			0.19	12.15	MARBCF$_1$
qMIC-C18-1	2014Yc	i13754Gh-i29829Gh	5.31	2.79	-0.04	0.20		13.28	IF$_2$
qMIC-C19-1	2014Yc	i27871Gh-i09066Gh	26.21	2.73			-0.06	16.14	HSBCF$_1$
	2015Bg	i55376Gb-i37157Gh	26.51	3.20			1.91	17.13	MARBCF$_1$
qMIC-C19-2	2014Yc	i09400Gh-i09637Gh	35.81	4.83		-0.15		17.42	MARBCF$_1$MPH
**qMIC-C24-1**	2014Bg	i25139Gh-i25656Gh	13.71	3.29			-0.21	18.02	MARBCF$_1$

	qMIC-C24-2	2014Bg	i15200Gh-i28524Gh	31.01	2.55		0.11		18.80	IF$_2$MPH
	qMIC-C24-3	2015Yc	i03702Gh-i35290Gh	53.01	2.85	0.11	-0.15		18.83	IF$_2$
	**qMIC-C24-4**	2015Yc	i04503Gh-i04704Gh	73.31	2.70			0.51	22.92	HSBCF$_1$
FE	qFE-C01-1	2015Yc	i41337Gh-i48009Gh	11.71	5.55			0.16	5.46	MARBCF$_1$
	qFE-C01-2	2015Bg	i02201Gh-i32863Gh	15.91	6.42		0.19		4.30	HSBCF$_1$MPH
		2014Yc	i23944Gh-i39024Gh	17.41	3.65		-0.70		24.18	IF$_2$MPH
	qFE-C01-3	2014Bg	i14664Gh-i25056Gh	25.71	3.58		-0.38		9.94	HSBCF$_1$MPH
	qFE-C02-1	2015Yc	i17680Gh-i02712Gh	23.11	2.71		0.07		5.51	IF$_2$MPH
		2014Yc	i02712Gh-i20804Gh	27.31	2.86		1.23		4.70	IF$_2$MPH
	qFE-C05-1	2015Bg	i20652Gh-i52543Gb	58.91	3.37			0.05	9.03	MARBCF$_1$
		2015Yc	i20652Gh-i35017Gh	58.91	2.75			0.07	3.73	MARBCF$_1$
	qFE-C06-1	2014Yc	i06111Gh-i05824Gh	8.81	3.90			-0.46	6.64	MARBCF$_1$
	qFE-C09-1	2015Yc	i05712Gh-i48103Gh	20.51	2.53		-0.03		5.36	MARBCF$_1$MPH
	qFE-C09-2	2015Bg	i05664Gh-i40760Gh	25.01	4.07			0.39	3.74	MARBCF$_1$
	qFE-C09-3	2015Bg	i03687Gh-i02498Gh	50.61	5.88		0.09		3.39	HSBCF$_1$MPH
	qFE-C09-4	2014Yc	i15489Gh-i22655Gh	57.61	2.72		0.47		3.46	MARBCF$_1$MPH
	qFE-C10-1	2015Bg	i12268Gh-i32655Gh	38.01	4.06			-0.08	17.04	MARBCF$_1$
		2015Bg	i12268Gh-i32655Gh	38.01	4.66		0.09		16.24	MARBCF$_1$MPH
	qFE-C13-1	2014Bg	i32083Gh-i18150Gh	33.41	4.05			-0.13	10.12	MARBCF$_1$
	qFE-C13-2	2015Bg	i33841Gh-i32206Gh	38.91	2.65	-0.03	-0.02		4.72	IF$_2$
	qFE-C14-1	2014Yc	i15284Gh-i48509Gh	5.31	2.51			1.75	10.58	HSBCF$_1$
		2015Bg	i15284Gh-i48509Gh	5.31	5.79			-0.36	5.25	MARBCF$_1$
		2015Bg	i15284Gh-i48509Gh	5.31	5.28		-0.48		5.13	MARBCF$_1$MPH
	qFE-C14-2	2014Yc	i05716Gh-i43468Gh	9.51	3.32			0.22	8.53	MARBCF$_1$
	qFE-C14-3	2014Yc	i15343Gh-i21369Gh	15.81	5.77			0.32	13.17	MARBCF$_1$
	qFE-C14-4	2015Yc	i04916Gh-i05024Gh	20.51	3.19			0.01	9.36	HSBCF$_1$

		2014Yc	i40777Gh-i43206Gh	22.11	2.64			-0.07	7.47	MARBCF$_1$
	qFE-C14-5	2014Yc	i38481Gh-i27231Gh	44.41	4.01		-0.59		10.92	IF$_2$MPH
		2015Bg	i38809Gh-i15488Gh	45.01	3.28		-0.11		3.81	MARBCF$_1$MPH
	qFE-C15-1	2015Yc	i21698Gh-i24483Gh	25.31	2.62		0.06		5.17	MARBCF$_1$MPH
		2015Yc	i25137Gh-i02486Gh	27.81	2.76			-0.05	7.70	HSBCF$_1$
	qFE-C16-1	2015Bg	i01711Gh-i36784Gh	4.31	3.05			0.04	22.65	MARBCF$_1$
	qFE-C17-1	2014Yc	i43615Gh-i14513Gh	33.41	2.99			-0.17	6.34	HSBCF$_1$
	qFE-C19-1	2015Yc	i08832Gh-i09430Gh	51.51	3.70			0.11	4.21	MARBCF$_1$
		2015Yc	i08832Gh-i09430Gh	52.21	3.63			0.08	6.73	HSBCF$_1$
	qFE-C20-1	2014Bg	i11735Gh-i42616Gh	29.71	2.54			0.01	18.89	HSBCF$_1$
		2014Yc	i11735Gh-i42616Gh	29.71	2.66			0.18	3.78	HSBCF$_1$
	qFE-C21-1	2015Bg	i06952Gh-i07714Gh	9.91	3.55			-0.04	12.81	MARBCF$_1$
		2015Yc	i06952Gh-i07714Gh	10.91	2.80		0.10		17.29	MARBCF$_1$MPH
	qFE-C21-2	2014Yc	i07714Gh-i38909Gh	20.11	4.72			-1.83	3.92	HSBCF$_1$
	qFE-C22-1	2015Bg	i20168Gh-i39918Gh	10.71	3.07			0.03	10.57	MARBCF$_1$
		2014Bg	i30763Gh-i17698Gh	11.61	3.94	1.47	0.12		14.82	IF$_2$
	qFE-C24-1	2014Bg	i04470Gh-i37272Gh	27.01	2.71			0.22	5.26	MARBCF$_1$
	qFE-C24-2	2014Bg	i04688Gh-i04069Gh	38.11	4.35		-0.28		8.97	MARBCF$_1$MPH
		2015Bg	i31637Gh-i15169Gh	40.11	4.80		0.15		23.41	HSBCF$_1$MPH
	qFE-C26-1	2015Bg	i16464Gh-i47876Gh	50.21	3.03			-0.02	16.96	MARBCF$_1$
		2015Bg	i28856Gh-i23175Gh	51.31	2.80		-0.35		19.60	MARBCF$_1$MPH
FS	qFS-C01-1	2014Yc	i48454Gh-i38692Gh	21.41	3.69	-0.38	1.15		7.32	IF$_2$
	qFS-C03-1	2014Yc	i30069Gh-i42939Gh	78.81	3.04			-0.22	8.88	MARBCF$_1$
		2014Yc	i43226Gh-i21218Gh	78.81	3.95		-1.19		10.32	MARBCF$_1$MPH
	qFS-C06-1	2015Bg	i06036Gh-i06505Gh	35.01	2.66	-1.38	2.20		24.73	IF$_2$
	qFS-C07-1	2015Bg	i01696Gh-i01453Gh	21.91	2.64		0.06		9.10	MARBCF$_1$MPH

	2015Yc	i01453Gh-i33174Gh	23.21	2.84		1.46		18.41	MARBCF$_1$MPH
qFS-C07-2	2014Yc	i36434Gh-i22167Gh	43.11	2.68			2.59	6.51	MARBCF$_1$
qFS-C08-1	2014Bg	i04524Gh-i04719Gh	45.01	3.19			-0.05	12.66	HSBCF$_1$
qFS-C09-1	2015Bg	i06090Gh-i02309Gh	38.71	4.13			-0.78	10.60	HSBCF$_1$
qFS-C09-2	2015Yc	i08546Gh-i03687Gh	49.81	3.38	1.17	-1.03		17.70	IF$_2$
	2015Yc	i03687Gh-i02498Gh	50.61	3.02		-2.05		17.51	HSBCF$_1$MPH
qFS-C09-3	2015Yc	i22037Gh-i05194Gh	59.01	2.54		0.98		3.70	MARBCF$_1$MPH
qFS-C13-1	2014Yc	i30934Gh-i18151Gh	19.41	3.54			-0.27	11.50	HSBCF$_1$
	2015Bg	i30934Gh-i32650Gh	20.41	2.51			0.19	10.25	MARBCF$_1$
qFS-C13-2	2015Yc	i32083Gh-i18150Gh	32.41	3.05		0.61		8.17	MARBCF$_1$MPH
qFS-C13-3	2015Yc	i20297Gh-i37629Gh	39.21	4.88		-2.12		3.32	HSBCF$_1$MPH
qFS-C14-1	2014Yc	i34963Gh-i36740Gh	50.51	4.07			-3.12	5.62	HSBCF$_1$
qFS-C15-1	2014Yc	i02469Gh-i02538Gh	15.61	3.03			-0.01	5.90	HSBCF$_1$
qFS-C16-1	2014Bg	i01627Gh-i22690Gh	45.61	2.65			-1.48	7.20	MARBCF$_1$
qFS-C17-1	2014Yc	i03525Gh-i14513Gh	31.01	2.98			0.75	5.58	MARBCF$_1$
qFS-C18-1	2014Yc	i31442Gh-i20346Gh	20.11	3.05		1.87		7.02	MARBCF$_1$MPH
qFS-C19-1	2014Bg	i16662Gh-i09629Gh	45.31	3.30	-0.43	0.88		7.29	IF$_2$
qFS-C19-2	2014Bg	i08832Gh-i09430Gh	52.21	2.66			0.87	6.20	MARBCF$_1$
qFS-C19-3	2015Yc	i10445Gh-i08852Gh	57.11	5.47			-2.76	7.67	HSBCF$_1$
**qFS-C20-1**	2014Yc	i36341Gh-i37554Gh	39.01	2.77			-0.82	13.06	HSBCF$_1$
	2015Bg	i36341Gh-i26441Gh	39.01	2.79			-1.10	16.94	HSBCF$_1$
qFS-C20-2	2014Bg	i11915Gh-i11478Gh	68.61	2.72			-2.14	10.13	MARBCF$_1$
qFS-C22-1	2014Bg	i17853Gh-i12725Gh	31.21	3.84	-1.45	-2.40		5.35	IF$_2$
qFS-C22-2	2014Bg	i12818Gh-i17697Gh	39.01	2.59	0.30	2.07		13.28	IF$_2$
qFS-C24-1	2015Yc	i04718Gh-i33113Gh	59.01	2.57		1.23		3.24	MARBCF$_1$MPH
	2015Bg	i03705Gh-i03832Gh	60.61	3.04		-0.83		5.05	MARBCF$_1$MPH

qFS-C25-1	2014Bg	i30564Gh-i36160Gh	2.81	2.57		0.28		6.55	IF_2MPH
qFS-C25-2	2014Bg	i29568Gh-i33416Gh	37.01	3.02	0.98	1.99		6.85	IF_2
	2015Yc	i20999Gh-i22495Gh	39.51	2.62			-3.91	4.27	$HSBCF_1$
	2014Yc	i20999Gh-i22495Gh	39.81	2.67	-1.50	-0.71		5.31	IF_2
	2015Bg	i20999Gh-i22495Gh	39.81	3.70			-3.67	6.19	$HSBCF_1$

[a] FL: fiber length; FU: fiber uniformity; MIC: micronaire; FE: fiber elongation; FS: fiber strength

[b] QTLs in bold are those also identified by CIM in RILs in our previous study (Li et al. 2016)

[c] 2014Yc: Yacheng of Hainan Province in 2014; 2014Bg: Baogang of Hainan Province in 2014; 2015Yc: Yacheng of Hainan Province in 2015; 2015Bg: Baogang of Hainan Province in 2015

[d] Position of QTL located on chromosome: as cM distance from the top of each chromosome

[e] A LOD threshold was used for declaration of QTL based on 1000 permutations at as significance level of 0.01

[f] The genetic expectation of a QTL effect obtained is the additive effect (A) and dominant effect (D) when estimated from the IF_2s, the additive and dominance effects (A+D) from the BCF_1s, and the dominance effect (D) from the MPH values

[g] Phenotypic variance explained by QTL
