# Supplementary material for: QTL Mapping and Heterosis Analysis for Fiber Quality Traits Across Multiple Genetic Populations and Environments in Upland Cotton
Source: Front Plant Sci. 2018 Oct 15;9:1364. doi: 10.3389/fpls.2018.01364 (PMC6196769; doi:10.3389/fpls.2018.01364)
Supplement: Supplementary file 4 [file Data_Sheet_4.PDF]

Table S4 Gene action of QTLs identified by CIM in IF_2 population across four environments

Trait[a]	QTL[b]	Env.[c]	Position[d]	A[e]	D[e]	|D/A|[f]	GA[g]	Population
FL	qFL-C02-1	2015Bg	43.91		-2.19		OD	IF_2MPH
	qFL-C02-2	2015Bg	75.71		0.56		OD	IF_2MPH
	qFL-C03-1	2014Bg	94.91	0.02	-0.77	31.62	OD	IF_2
	qFL-C04-1	2014Bg	3.01	-0.27	-1.03	3.81	OD	IF_2
	**qFL-Chr05-3**	2014Bg	11.21	-1.16				RIL
	qFL-C05-1	2014Yc	13.01	2.68	-3.14	1.17	OD	IF_2
	**qFL-Chr05-4**	2014Bg	40.01	-0.32				RIL
	**qFL-Chr05-1**	2014Yc	45.31	-0.37				RIL
	**qFL-Chr05-2**	2014Yc	52.11	-0.33				RIL
	qFL-C06-1	2015Bg	25.61		0.49		OD	IF_2MPH
		2014Yc	27.81	2.78	-2.86	1.03		IF_2
	qFL-C07-1	2014Yc	49.81	2.77	-3.00	1.08	OD	IF_2
	qFL-C08-1	2014Yc	16.71	2.91	-2.70	0.93	PD	IF_2
	**qFL-Chr09-1**	2014Bg	3.81	-0.30				RIL
	qFL-C09-2	2015Yc	32.71	0.36	-0.39	1.08	OD/PD	IF_2
		2015Bg	32.71	0.48	-0.14	0.29		IF_2
	qFL-C10-1	2014Yc	36.81	2.77	-3.00	1.08	OD	IF_2
	**qFL-Chr10-1**	2014Yc	44.51	0.29				RIL
		2014Bg	44.51	0.26				RIL
	**qFL-Chr12-1**	2014Yc	16.21	-0.33				RIL
	qFL-C13-1	2014Yc	4.91	2.87	-2.64	0.92	PD	IF_2
	qFL-C14-1	2014Yc	4.01	2.85	-2.75	0.97	PD/OD	IF_2
		2015Bg	6.31	0.26	1.77	6.81		IF_2
	**qFL-Chr14-1**	2014Yc	14.21	-0.50				RIL
	**qFL-Chr14-2**	2014Yc	20.91	-0.41				RIL
	**qFL-Chr14-3**	2014Yc	23.31	-0.46				RIL
		2014Bg	25.71	-0.27				RIL
	**qFL-Chr15-1**	2014Yc	13.11	-0.36				RIL
		2014Bg	12.31	-0.38				RIL
	qFL-C16-2	2015Yc	57.01	-0.32	0.15	0.47	PD/OD	IF_2
		2015Bg	57.21	-0.10	0.71	7.05		IF_2
		2015Bg	57.21		0.79			IF_2MPH
	qFL-C17-1	2014Yc	35.11	2.74	-2.99	1.09	OD	IF_2
	**qFL-Chr19-1**	2014Yc	22.51	0.46				RIL
	qFL-C20-1	2015Yc	4.81	-0.34	-0.38	1.10	OD	IF_2
	qFL-C20-2	2014Bg	8.01		-0.61		PD	IF_2MPH
		2015Bg	8.01	-0.81	-0.28	0.35		IF_2
		2015Yc	9.91	-0.45	-0.39	0.87		IF_2
	qFL-C20-3	2015Bg	13.11	-0.51	-0.13	0.25	PD	IF_2
	qFL-C22-3	2014Bg	20.11		0.37		OD	IF_2MPH
	qFL-C22-4	2014Bg	30.21		0.39		OD	IF_2MPH
	qFL-C24-2	2015Yc	29.41	-0.29	-0.22	0.74	PD	IF_2

FU	**qFU-Chr01-1**	2014Bg	14.51	-0.61				RIL
	qFU-C01-1	2014Yc	35.71	1.43	-1.45	1.02	OD	IF_2
	qFU-C03-1	2015Bg	50.51	-0.76	0.64	0.84	PD	IF_2
		2015Bg	52.51		0.48			IF_2MPH
	qFU-C03-2	2014Bg	69.11		-0.66		OD	IF_2MPH
	qFU-C05-1	2015Bg	40.01	1.06	1.77	1.67	OD	IF_2
	**qFU-Chr05-1**	2014Bg	45.31	-0.22				RIL
	qFU-C05-2	2015Bg	46.91	-0.33	0.65	1.95	OD	IF_2
		2015Bg	46.91		0.64			IF_2MPH
	qFU-C06-1	2015Bg	26.61	1.16	1.76	1.52	OD	IF_2
	qFU-C06-2	2015Yc	36.01	-0.40	1.06	2.65	OD	IF_2
		2015Yc	36.01		1.12			IF_2MPH
	**qFU-Chr09-1**	2014Yc	3.81	-0.24				RIL
		2014Bg	3.81	-0.29				RIL
	**qFU-Chr09-5**	2014Bg	18.11	-0.24				RIL
	qFU-C09-3	2015Bg	30.31	-3.23	3.18	0.98	PD	IF_2
	**qFU-Chr09-2**	2014Yc	47.11	-0.24				RIL
		2014Bg	47.11	-0.24				RIL
	**qFU-Chr09-3**	2014Yc	52.61	0.30				RIL
		2014Bg	52.61	0.29				RIL
	qFU-C09-4	2014Bg	54.71	0.30	0.08	0.26	PD	IF_2
	**qFU-Chr09-4**	2014Yc	61.41	-0.20				RIL
	qFU-C11-1	2015Bg	5.31	-1.99	3.20	1.61	OD	IF_2
		2015Bg	5.31		3.44			IF_2MPH
	qFU-C13-1	2014Bg	30.21	-0.38	0.07	0.18	PD	IF_2
	qFU-C14-1	2015Bg	1.11	-3.22	3.31	1.03	OD	IF_2
		2015Bg	1.11		4.14			IF_2MPH
	**qFU-Chr19-1**	2014Yc	20.81	-0.22				RIL
	qFU-C20-1	2014Yc	31.11	0.41	-1.11	2.69	OD	IF_2
	qFU-C20-2	2014Yc	41.51	-0.34	-0.38	1.11	OD	IF_2
	qFU-C21-1	2015Yc	44.61		0.01		OD	IF_2MPH
	qFU-C21-2	2015Yc	58.91		-0.10		OD	IF_2MPH
	qFU-C22-2	2015Yc	29.31	-0.19	0.50	2.66	OD	IF_2
	qFU-C24-1	2014Bg	19.91	0.32	-0.33	1.02	OD	IF_2
	qFU-C25-1	2015Yc	41.91	-0.45	1.04	2.31	OD	IF_2
	qFU-C25-2	2014Bg	51.81	-0.34	0.16	0.48	PD	IF_2
MIC	qMIC-C01-1	2015Bg	7.51	0.05	-0.21	3.99	OD	IF_2
	**qMIC-Chr01-1**	2014Yc	14.51	0.24				RIL
	qMIC-C01-2	2014Yc	15.51	0.13	-0.24	1.80	OD	IF_2
	qMIC-C01-3	2015Yc	24.91		0.57		OD	IF_2MPH
	qMIC-C01-4	2015Yc	45.71	-0.30	-0.26	0.87	PD	IF_2
		2015Yc	45.71		-0.32			IF_2MPH
	**qMIC-Chr05-1**	2014Yc	12.61	-0.25				RIL
	**qMIC-Chr07-1**	2014Bg	59.31	0.10				RIL

	qMIC-C08-1	2015Yc	35.61		-0.70		OD	IF$_2$MPH
		2014Yc	38.01		-0.58			IF$_2$MPH
	**qMIC-Chr10-1**	2014Yc	62.61	0.16				RIL
	qMIC-C13-2	2015Bg	33.81	-0.12	0.10	0.90	PD	IF$_2$
	qMIC-C13-3	2015Bg	40.91	-0.12	0.12	1.00	PD	IF$_2$
		2014Bg	20.91	0.11				RIL
	**qMIC-Chr14-1**	2014Yc	23.31	0.08				RIL
	qMIC-C14-2	2014Yc	31.21	0.07	-0.22	3.07	OD	IF$_2$
	qMIC-C15-2	2015Yc	32.81		0.53		OD	IF$_2$MPH
	qMIC-C16-1	2015Bg	8.71	0.14	-0.16	1.13	OD	IF$_2$
		2014Bg	49.31	0.09				RIL
	**qMIC-Chr16-1**	2014Yc	51.01	0.08				RIL
	**qMIC-Chr16-2**	2014Yc	57.01	0.09				RIL
		2014Bg	57.41	0.15				RIL
	**qMIC-Chr17-1**	2014Bg	44.81	0.12				RIL
	qMIC-C18-1	2014Yc	5.31	-0.04	0.20	4.43	OD	IF$_2$
	**qMIC-Chr24-1**	2014Bg	16.81	-0.11				RIL
	qMIC-C24-2	2014Bg	31.01		0.11		OD	IF$_2$MPH
	qMIC-C24-3	2015Yc	53.01	0.11	-0.15	1.34	OD	IF$_2$
	**qMIC-Chr24-2**	2014Bg	73.31	0.44				RIL
FE	qFE-C01-2	2014Yc	17.41		-0.70		OD	IF$_2$MPH
	qFE-C02-1	2015Yc	23.11		0.07		OD	IF$_2$MPH
		2014Yc	27.31		1.23			IF$_2$MPH
	**qFE-Chr11-1**	2014Bg	5.31	1.16				RIL
	qFE-C13-2	2015Bg	38.91	-0.03	-0.02	0.60	PD	IF$_2$
	**qFE-Chr14-2**	2014Bg	1.11	1.09				RIL
	**qFE-Chr14-3**	2014Bg	6.31	0.25				RIL
		2014Bg	15.71	0.26				RIL
	**qFE-Chr14-1**	2014Yc	16.81	0.30				RIL
	qFE-C14-5	2014Yc	44.41		-0.59		OD	IF$_2$MPH
	**qFE-Chr16-1**	2014Bg	1.11	1.17				RIL
	**qFE-Chr17-1**	2014Bg	42.31	0.18				RIL
	**qFE- Chr18-1**	2014Bg	57.51	0.76				RIL
	**qFE-Chr20-1**	2014Yc	41.51	0.20				RIL
		2014Bg	47.11	0.44				RIL
	qFE-C22-1	2014Bg	11.61	1.47	0.12	0.08	PD	IF$_2$
	**qFE-Chr24-1**	2014Yc	73.31	0.75				RIL
		2014Bg	73.31	0.62				RIL
FS	qFS-C01-1	2014Yc	21.41	-0.38	1.15	3.05	OD	IF$_2$
	**qFS-Chr05-1**	2014Bg	54.81	0.52				RIL
	qFS-C06-1	2015Bg	35.01	-1.38	2.20	1.59	OD	IF$_2$
	qFS-C09-2	2015Yc	49.81	1.17	-1.03	0.88	PD	IF$_2$
	**qFS-Chr14-1**	2014Yc	6.71	-0.21				RIL
	**qFS-Chr14-2**	2014Yc	14.21	-0.28				RIL

**qFS-Chr14-3**	2014Yc	21.61	-0.26				RIL
**qFS-Chr14-4**	2014Yc	40.11	-0.20				RIL
**qFS-Chr19-1**	2014Bg	22.51	0.51				RIL
**qFS-Chr19-2**	2014Bg	27.81	0.61				RIL
qFS-C19-1	2014Bg	45.31	-0.43	0.88	2.03	OD	IF$_2$
**qFS-Chr20-1**	2014Yc	42.11	-0.50				RIL
**qFS-Chr20-2**	2014Yc	59.61	-0.48				RIL
qFS-C22-1	2014Bg	31.21	-1.45	-2.40	1.66	OD	IF$_2$
qFS-C22-2	2014Bg	39.01	0.30	2.07	7.00	OD	IF$_2$
qFS-C25-1	2014Bg	2.81		0.28		OD	IF$_2$MPH
qFS-C25-2	2014Bg	37.01	0.98	1.99	2.03	OD/PD	IF$_2$
	2014Yc	39.81	-1.50	-0.71	0.47		IF$_2$

[a] FL: fiber length; FU: fiber uniformity; MIC: micronaire; FE: fiber elongation; FS: fiber strength

[b] QTLs in bold are those identified by CIM in RILs in our previous study (Li et al. 2016), which was just used to estimate the gene action of IF$_2$ population

[c] 2014Yc: Yacheng of Hainan Province in 2014; 2014Bg: Baogang of Hainan Province in 2014; 2015Yc: Yacheng of Hainan Province in 2015; 2015Bg: Baogang of Hainan Province in 2015

[d] Position of QTL located on chromosome: as cM distance from the top of each chromosome

[e] The genetic expectation of a QTL effect obtained is the additive effect (A) from the RILs, the the additive effect (A) and dominant effect (D) when estimated from the IF$_2$s, and the dominance effect (D) from the MPH values

[f] |D/A|: |dominance/additive|

[g] GA: gene action; PD/D partial dominance (|d/a| <= 1); OD overdominance(|d/a| > 1)
