# Supplementary material for: QTL Mapping and Heterosis Analysis for Fiber Quality Traits Across Multiple Genetic Populations and Environments in Upland Cotton
Source: Front Plant Sci. 2018 Oct 15;9:1364. doi: 10.3389/fpls.2018.01364 (PMC6196769; doi:10.3389/fpls.2018.01364)
Supplement: Supplementary file 5 [file Data_Sheet_5.pdf]

Table S5 Gene action of QTLs identified by CIM in HSBCF$_1$ population across four environments

<table>
<tr><th>Trait[a]</th><th>QTL[b]</th><th>Env.[c]</th><th>Position[d]</th><th>A[e]</th><th>D[e]</th><th>A+D[e]</th><th>|D/A|or 2|D|/|(A+D)|[f]</th><th>GA[g]</th><th>Population</th></tr>
<tr><td>FL</td><td>qFL-C02-2</td><td>2015Yc</td><td>78.11</td><td></td><td>0.18</td><td></td><td></td><td>OD</td><td>HSBCF$_1$MPH</td></tr>
<tr><td></td><td>**qFL-Chr05-3**</td><td>2014Bg</td><td>11.21</td><td>-1.16</td><td></td><td></td><td></td><td></td><td>RIL</td></tr>
<tr><td></td><td>**qFL-Chr05-4**</td><td>2014Bg</td><td>40.01</td><td>-0.32</td><td></td><td></td><td></td><td></td><td>RIL</td></tr>
<tr><td></td><td>qFL-C05-2</td><td>2015Bg</td><td>43.21</td><td></td><td></td><td>0.04</td><td></td><td>A</td><td>HSBCF$_1$</td></tr>
<tr><td></td><td>**qFL-Chr05-1**</td><td>2014Yc</td><td>45.31</td><td>-0.37</td><td></td><td></td><td></td><td></td><td>RIL</td></tr>
<tr><td></td><td>qFL-C05-3</td><td>2015Bg</td><td>50.81</td><td></td><td></td><td>0.24</td><td>6.21</td><td>OD</td><td>HSBCF$_1$</td></tr>
<tr><td></td><td></td><td>2014Yc</td><td>51.61</td><td></td><td></td><td>1.25</td><td>1.21</td><td></td><td>HSBCF$_1$</td></tr>
<tr><td></td><td></td><td>2014Yc</td><td>50.81</td><td></td><td>0.76</td><td></td><td></td><td></td><td>HSBCF$_1$MPH</td></tr>
<tr><td></td><td>**qFL-Chr05-2**</td><td>2014Yc</td><td>52.11</td><td>-0.33</td><td></td><td></td><td></td><td></td><td>RIL</td></tr>
<tr><td></td><td>**qFL-Chr09-1**</td><td>2014Bg</td><td>3.81</td><td>-0.30</td><td></td><td></td><td></td><td></td><td>RIL</td></tr>
<tr><td></td><td>qFL-C09-1</td><td>2014Bg</td><td>12.91</td><td></td><td></td><td>-0.17</td><td></td><td>A</td><td>HSBCF$_1$</td></tr>
<tr><td></td><td>qFL-C09-3</td><td>2015Bg</td><td>46.51</td><td></td><td></td><td>0.86</td><td></td><td>A</td><td>HSBCF$_1$</td></tr>
<tr><td></td><td>**qFL-Chr10-1**</td><td>2014Yc</td><td>44.51</td><td>0.29</td><td></td><td></td><td></td><td></td><td>RIL</td></tr>
<tr><td></td><td>qFL-C11-1</td><td>2015Yc</td><td>4.81</td><td></td><td></td><td>-1.73</td><td></td><td>A</td><td>HSBCF$_1$</td></tr>
<tr><td></td><td></td><td>2014Bg</td><td>44.51</td><td>0.26</td><td></td><td></td><td></td><td></td><td>RIL</td></tr>
<tr><td></td><td>**qFL-Chr12-1**</td><td>2014Yc</td><td>16.21</td><td>-0.33</td><td></td><td></td><td></td><td></td><td>RIL</td></tr>
<tr><td></td><td>qFL-C13-2</td><td>2015Yc</td><td>30.61</td><td></td><td></td><td>0.56</td><td></td><td>A</td><td>HSBCF$_1$</td></tr>
<tr><td></td><td>**qFL-Chr14-1**</td><td>2014Yc</td><td>14.21</td><td>-0.50</td><td></td><td></td><td></td><td></td><td>RIL</td></tr>
<tr><td></td><td>**qFL-Chr14-2**</td><td>2014Yc</td><td>20.91</td><td>-0.41</td><td></td><td></td><td></td><td></td><td>RIL</td></tr>
<tr><td></td><td>qFL-C14-4</td><td>2015Bg</td><td>23.21</td><td></td><td></td><td>-0.83</td><td></td><td>A</td><td>HSBCF$_1$</td></tr>
<tr><td></td><td>**qFL-Chr14-3**</td><td>2014Yc</td><td>23.31</td><td>-0.46</td><td></td><td></td><td></td><td></td><td>RIL</td></tr>
<tr><td></td><td></td><td>2014Bg</td><td>25.71</td><td>-0.27</td><td></td><td></td><td></td><td></td><td>RIL</td></tr>
<tr><td></td><td>**qFL-Chr15-1**</td><td>2014Yc</td><td>13.11</td><td>-0.36</td><td></td><td></td><td></td><td></td><td>RIL</td></tr>
<tr><td></td><td>qFL-C16-1</td><td>2014Yc</td><td>6.21</td><td></td><td></td><td>0.84</td><td></td><td>A</td><td>HSBCF$_1$</td></tr>
<tr><td></td><td></td><td>2014Bg</td><td>12.31</td><td>-0.38</td><td></td><td></td><td></td><td></td><td>RIL</td></tr>
<tr><td></td><td>qFL-C17-1</td><td>2014Bg</td><td>31.31</td><td></td><td></td><td>-0.08</td><td></td><td>A</td><td>HSBCF$_1$</td></tr>
<tr><td></td><td>qFL-C18-1</td><td>2014Bg</td><td>59.01</td><td></td><td>-2.68</td><td></td><td></td><td>OD</td><td>HSBCF$_1$MPH</td></tr>
<tr><td></td><td>qFL-C19-1</td><td>2014Yc</td><td>16.01</td><td></td><td></td><td>0.51</td><td>3.13</td><td>OD</td><td>HSBCF$_1$</td></tr>
<tr><td></td><td></td><td>2014Yc</td><td>16.01</td><td></td><td>0.80</td><td></td><td></td><td></td><td>HSBCF$_1$MPH</td></tr>
<tr><td></td><td>**qFL-Chr19-1**</td><td>2014Yc</td><td>22.51</td><td>0.46</td><td></td><td></td><td></td><td></td><td>RIL</td></tr>
<tr><td></td><td>qFL-C19-3</td><td>2014Yc</td><td>26.51</td><td></td><td></td><td>-0.73</td><td></td><td>A</td><td>HSBCF$_1$</td></tr>
<tr><td></td><td>qFL-C20-4</td><td>2014Yc</td><td>42.11</td><td></td><td></td><td>-0.31</td><td></td><td>A</td><td>HSBCF$_1$</td></tr>
<tr><td></td><td></td><td>2015Bg</td><td>44.11</td><td></td><td></td><td>-0.45</td><td></td><td></td><td>HSBCF$_1$</td></tr>
<tr><td></td><td>qFL-C21-1</td><td>2014Bg</td><td>44.61</td><td></td><td>-0.76</td><td></td><td></td><td>OD</td><td>HSBCF$_1$MPH</td></tr>
<tr><td>FU</td><td>**qFU-Chr01-1**</td><td>2014Bg</td><td>14.51</td><td>-0.61</td><td></td><td></td><td></td><td></td><td>RIL</td></tr>
<tr><td></td><td>qFU-C01-1</td><td>2015Yc</td><td>30.31</td><td></td><td></td><td>0.89</td><td></td><td>A</td><td>HSBCF$_1$</td></tr>
<tr><td></td><td>qFU-C01-2</td><td>2014Bg</td><td>41.71</td><td></td><td></td><td>0.50</td><td>29.41</td><td>OD</td><td>HSBCF$_1$</td></tr>
<tr><td></td><td></td><td>2015Bg</td><td>45.71</td><td></td><td></td><td>-4.99</td><td>2.97</td><td></td><td>HSBCF$_1$</td></tr>
<tr><td></td><td></td><td>2015Bg</td><td>45.71</td><td></td><td>-7.40</td><td></td><td></td><td></td><td>HSBCF$_1$MPH</td></tr>
<tr><td></td><td>qFU-C01-3</td><td>2015Bg</td><td>50.41</td><td></td><td></td><td>-1.09</td><td></td><td>A</td><td>HSBCF$_1$</td></tr>
<tr><td></td><td>qFU-C02-1</td><td>2015Bg</td><td>44.81</td><td></td><td></td><td>-4.44</td><td>2.72</td><td>OD</td><td>HSBCF$_1$</td></tr>
</table>

		2015Bg	44.81		-6.04				HSBCF$_1$MPH
	**qFU-Chr05-1**	2014Bg	45.31	-0.22					RIL
	qFU-C06-2	2015Bg	35.01			0.29	6.15	OD	HSBCF$_1$
		2015Yc	36.01		-0.90				HSBCF$_1$MPH
	qFU-C06-3	2015Bg	47.21			0.19		A	HSBCF$_1$
	**qFU-Chr09-1**	2014Yc	3.81	-0.24					RIL
		2014Bg	3.81	-0.29					RIL
	qFU-C09-1	2014Bg	17.81		27.70	-113.49		OD	HSBCF$_1$MPH
	**qFU-Chr09-5**	2014Bg	18.11	-0.24					RIL
	qFU-C09-2	2015Bg	25.01			9.04		A	HSBCF$_1$
	**qFU-Chr09-2**	2014Yc	47.11	-0.24					RIL
		2014Bg	47.11	-0.24					RIL
	qFU-C09-4	2014Yc	49.61			1.35		A	HSBCF$_1$
		2015Bg	53.01			9.05			HSBCF$_1$
	**qFU-Chr09-3**	2014Yc	52.61	0.30					RIL
		2014Bg	52.61	0.29					RIL
	**qFU-Chr09-4**	2014Yc	61.41	-0.20					RIL
	qFU-C13-1	2014Bg	32.41			0.31		A	HSBCF$_1$
	qFU-C14-2	2015Yc	37.41			0.16		A	HSBCF$_1$
	qFU-C16-1	2015Bg	72.91			-0.04	233.55	OD	HSBCF$_1$
		2015Bg	72.91		4.93				HSBCF$_1$MPH
	qFU-C17-1	2014Bg	27.91			-0.19		A	HSBCF$_1$
	qFU-C18-1	2015Bg	57.11			-9.44		A	HSBCF$_1$
	**qFU-Chr19-1**	2014Yc	20.81	-0.22					RIL
	qFU-C19-1	2014Bg	37.41			1.04		A	HSBCF$_1$
	qFU-C20-2	2015Bg	38.01			-0.09	107.50	OD	HSBCF$_1$
		2015Bg	38.01		4.69				HSBCF$_1$MPH
	qFU-C20-3	2015Yc	49.11		-0.70			OD	HSBCF$_1$MPH
	qFU-C20-4	2015Bg	57.81			-3.54	3.04	OD	HSBCF$_1$
		2015Bg	57.81		-5.39				HSBCF$_1$MPH
	qFU-C22-1	2015Yc	20.81			-0.72		A	HSBCF$_1$
	qFU-C26-1	2015Yc	2.51		0.39			OD	HSBCF$_1$MPH
	qFU-C26-2	2014Yc	38.31			0.32		A	HSBCF$_1$
MIC	**qMIC-Chr01-1**	2014Yc	14.51	0.24					RIL
	qMIC-C02-1	2015Yc	32.21			0.97		A	HSBCF$_1$
	**qMIC-Chr05-1**	2014Yc	12.61	-0.25					RIL
	qMIC-C05-1	2014Bg	48.31			0.53		A	HSBCF$_1$
	**qMIC-Chr07-1**	2014Bg	59.31	0.10					RIL
	qMIC-C08-1	2015Bg	35.91		0.68			OD	HSBCF$_1$MPH
	qMIC-C09-1	2015Yc	18.11			0.24		A	HSBCF$_1$
	qMIC-C11-1	2015Yc	17.11			0.41	0.03	PD	HSBCF$_1$
		2015Yc	18.11		0.01				HSBCF$_1$MPH
	**qMIC-Chr10-1**	2014Yc	62.61	0.16					RIL

	qMIC-C13-3	2014Yc	44.11			0.08		A	HSBCF$_1$
	qMIC-C14-1	2015Yc	4.01			0.74		A	HSBCF$_1$
	**qMIC-Chr14-1**	2014Yc	23.31	0.08					RIL
		2014Bg	20.91	0.11					RIL
	qMIC-C14-2	2014Yc	27.21			0.21		A	HSBCF$_1$
		2015Bg	30.91			0.01			HSBCF$_1$
		2015Yc	32.71			0.30			HSBCF$_1$
	qMIC-C14-3	2015Yc	43.81			-0.01		A	HSBCF$_1$
	qMIC-C15-1	2015Bg	20.51			0.27		PD	HSBCF$_1$
		2015Yc	20.51		0.07		0.50		HSBCF$_1$MPH
		2015Bg	20.51		0.07		0.50		HSBCF$_1$MPH
	**qMIC-Chr16-1**	2014Yc	51.01	0.08					RIL
		2014Bg	49.31	0.09					RIL
	**qMIC-Chr16-2**	2014Yc	57.01	0.09					RIL
		2014Bg	57.41	0.15					RIL
	**qMIC-Chr17-1**	2014Bg	44.81	0.12					RIL
	qMIC-C19-1	2014Yc	26.21			-0.06		A	HSBCF$_1$
	**qMIC-Chr24-1**	2014Bg	16.81	-0.11					RIL
	qMIC-C24-4	2015Yc	73.31			0.51		A	HSBCF$_1$
	**qMIC-Chr24-2**	2014Bg	73.31	0.44					RIL
FE	qFE-C01-2	2015Bg	15.91		0.19			OD	HSBCF$_1$MPH
	qFE-C01-3	2014Bg	25.71		-0.38			OD	HSBCF$_1$MPH
	qFE-C09-3	2015Bg	50.61		0.09			OD	HSBCF$_1$MPH
	**qFE-Chr11-1**	2014Bg	5.31	1.16					RIL
	**qFE-Chr14-2**	2014Bg	1.11	1.09					RIL
	qFE-C14-1	2014Yc	5.31			1.75		A	HSBCF$_1$
	**qFE-Chr14-3**	2014Bg	6.31	0.25					RIL
		2014Bg	15.71	0.26					RIL
	**qFE-Chr14-1**	2014Yc	16.81	0.30					RIL
	qFE-C14-4	2015Yc	20.51			0.01		A	HSBCF$_1$
	qFE-C15-1	2015Yc	27.81			-0.05		A	HSBCF$_1$
	**qFE-Chr16-1**	2014Bg	1.11	1.17					RIL
	qFE-C17-1	2014Yc	33.41			-0.17		A	HSBCF$_1$
	**qFE-Chr17-1**	2014Bg	42.31	0.18					RIL
	qFE-C19-1	2015Yc	52.21			0.08		A	HSBCF$_1$
	**qFE-Chr18-1**	2014Bg	57.51	0.76					RIL
	qFE-C20-1	2014Yc	29.71			0.18		A	HSBCF$_1$
		2014Bg	29.71			0.01			HSBCF$_1$
	**qFE-Chr20-1**	2014Yc	41.51	0.20					RIL
		2014Bg	47.11	0.44					RIL
	qFE-C21-2	2014Yc	20.11			-1.83		A	HSBCF$_1$
	qFE-C24-2	2015Bg	40.11		0.15			OD	HSBCF$_1$MPH
	**qFE-Chr24-1**	2014Yc	73.31	0.75					RIL
		2014Bg	73.31	0.62					RIL

FS	**qFS-Chr05-1**	2014Bg	54.81	0.52						RIL
	qFS-C08-1	2014Bg	45.01			-0.05			A	HSBCF$_1$
	qFS-C09-1	2015Bg	38.71			-0.78			A	HSBCF$_1$
	qFS-C09-2	2015Yc	50.61		-2.05				OD	HSBCF$_1$MPH
	qFS-C13-1	2014Yc	19.41			-0.27			A	HSBCF$_1$
	qFS-C13-3	2015Yc	39.21		-2.12				OD	HSBCF$_1$MPH
	**qFS-Chr14-1**	2014Yc	6.71	-0.21						RIL
	**qFS-Chr14-2**	2014Yc	14.21	-0.28						RIL
	**qFS-Chr14-3**	2014Yc	21.61	-0.26						RIL
	**qFS-Chr14-4**	2014Yc	40.11	-0.20						RIL
	qFS-C14-1	2014Yc	50.51			-3.12			A	HSBCF$_1$
	qFS-C15-1	2014Yc	15.61			-0.01			A	HSBCF$_1$
	**qFS-Chr19-1**	2014Bg	22.51	0.51						RIL
	**qFS-Chr19-2**	2014Bg	27.81	0.61						RIL
	qFS-C19-3	2015Yc	57.11			-2.76			A	HSBCF$_1$
	qFS-C20-1	2014Yc	39.01			-0.82			A	HSBCF$_1$
		2015Bg	39.01			-1.10				HSBCF$_1$
	**qFS-Chr20-1**	2014Yc	42.11	-0.50						RIL
	**qFS-Chr20-2**	2014Yc	59.61	-0.48						RIL
	qFS-C25-2	2015Yc	39.51			-3.91			A	HSBCF$_1$
		2015Bg	39.81			-3.67				HSBCF$_1$

[a] FL: fiber length; FU: fiber uniformity; MIC: micronaire; FE: fiber elongation; FS: fiber strength

[b] QTLs in bold are those identified by CIM in RILs in our previous study (Li et al. 2016), which was just used to estimate the gene action of HSBCF$_1$ population

[c] 2014Yc: Yacheng of Hainan Province in 2014; 2014Bg: Baogang of Hainan Province in 2014; 2015Yc: Yacheng of Hainan Province in 2015; 2015Bg: Baogang of Hainan Province in 2015

[d] Position of QTL located on chromosome: as cM distance from the top of each chromosome

[e] The genetic expectation of a QTL effect obtained is the additive effect (A) from the RILs, the additive and dominance effects (A+D) from the BCF$_1$s, and the dominance effect (D) from the MPH values

[f] |D/A|: |dominance/additive|; 2|D|/|(A+D)|: 2| dominance |/|(additive + dominance)|

[g] GA: gene action; PD/D partial dominance (|d/a| <= 1 or 2|d|/|(a+d)| <= 1); OD overdominance(|d/a| >1 or 2|d|/|(a+d)| > 1), here, 2|d|/|(a+d)| > 1 same to $2|d| > |a + d|$; A: when QTL detected only in BCF$_1$ or both BCF$_1$ and RIL was referred to as additive (A).
