# Supplementary material for: QTL Mapping and Heterosis Analysis for Fiber Quality Traits Across Multiple Genetic Populations and Environments in Upland Cotton
Source: Front Plant Sci. 2018 Oct 15;9:1364. doi: 10.3389/fpls.2018.01364 (PMC6196769; doi:10.3389/fpls.2018.01364)
Supplement: Supplementary file 6 [file Data_Sheet_6.pdf]

Table S6 Gene action of QTLs identified by CIM in MARBCF$_1$ population across four environments

Trait[a]	QTL[b]	Env.[c]	Position[d]	A[e]	D[e]	A+D[e]	|D/A|or 2|D|/|(A+D)|[f]	GA[g]	Population
FL	qFL-C02-1	2014Bg	41.21		-2.15			OD	MARBCF$_1$MPH
	**qFL-Chr05-3**	2014Bg	11.21	-1.16					RIL
	**qFL-Chr05-4**	2014Bg	40.01	-0.32					RIL
	**qFL-Chr05-1**	2014Yc	45.31	-0.37					RIL
	qFL-C05-2	2014Bg	46.91			0.03		A	MARBCF$_1$
	**qFL-Chr05-2**	2014Yc	52.11	-0.33					RIL
	qFL-C06-1	2014Bg	27.81		-2.47			OD	MARBCF$_1$MPH
	qFL-C06-2	2015Yc	49.21			1.08		A	MARBCF$_1$
	**qFL-Chr09-1**	2014Bg	3.81	-0.30					RIL
	**qFL-Chr10-1**	2014Yc	44.51	0.29					RIL
		2014Bg	44.51	0.26					RIL
	**qFL-Chr12-1**	2014Yc	16.21	-0.33					RIL
	qFL-C13-3	2015Yc	42.51			-0.22		A	MARBCF$_1$
	qFL-C14-1	2014Bg	4.01		-2.61			OD	MARBCF$_1$MPH
	qFL-C14-2	2015Bg	13.81			-0.82		A	MARBCF$_1$
	**qFL-Chr14-1**	2014Yc	14.21	-0.50					RIL
	qFL-C14-3	2014Yc	20.91			-0.19	3.15	OD/PD	MARBCF$_1$
		2015Bg	20.91			-1.09	0.84		MARBCF$_1$
		2015Yc	18.41		-0.30				MARBCF$_1$MPH
	**qFL-Chr14-2**	2014Yc	20.91	-0.41					RIL
	**qFL-Chr14-3**	2014Yc	23.31	-0.46					RIL
		2014Bg	25.71	-0.27					RIL
	qFL-C14-4	2015Bg	24.71			-1.18		A	MARBCF$_1$
		2014Yc	28.21			-0.66			MARBCF$_1$
	**qFL-Chr15-1**	2014Yc	13.11	-0.36					RIL
		2014Bg	12.31	-0.38					RIL
	qFL-C17-2	2014Bg	42.31			0.15		A	MARBCF$_1$
	qFL-C18-1	2014Bg	58.61		-2.30			OD	MARBCF$_1$MPH
	qFL-C19-2	2015Yc	20.81			0.38		A	MARBCF$_1$
	**qFL-Chr19-1**	2014Yc	22.51	0.46					RIL
	qFL-C19-3	2015Yc	27.61			2.15		A	MARBCF$_1$
	qFL-C20-4	2014Bg	41.51			0.33		A	MARBCF$_1$
		2015Yc	41.51			0.12			MARBCF$_1$
	qFL-C22-1	2014Yc	7.51		0.86			OD	MARBCF$_1$MPH
	qFL-C22-2	2014Bg	16.71		-2.24			OD	MARBCF$_1$MPH
	qFL-C24-1	2015Bg	5.41		-1.27			OD	MARBCF$_1$MPH
FU	**qFU-Chr01-1**	2014Bg	14.51	-0.61					RIL
	qFU-C02-2	2014Yc	48.31			-0.27		A	MARBCF$_1$
	**qFU-Chr05-1**	2014Bg	45.31	-0.22					RIL
	qFU-C06-3	2014Yc	49.21			0.70		A	MARBCF$_1$

	**qFU-Chr09-1**	2014Yc	3.81	-0.24					RIL
		2014Bg	3.81	-0.29					RIL
	**qFU-Chr09-2**	2014Yc	47.11	-0.24					RIL
		2014Bg	47.11	-0.24					RIL
	**qFU-Chr09-3**	2014Yc	52.61	0.30					RIL
		2014Bg	52.61	0.29					RIL
	**qFU-Chr09-4**	2014Yc	61.41	-0.20					RIL
	**qFU-Chr09-5**	2014Bg	18.11	-0.24					RIL
	qFU-C13-1	2014Yc	29.11			2.32		A	MARBCF$_1$
	**qFU-Chr19-1**	2014Yc	20.81	-0.22					RIL
	qFU-C21-1	2015Bg	44.61		-1.43			OD	MARBCF$_1$MPH
	qFU-C21-2	2015Yc	59.91		0.63			OD	MARBCF$_1$MPH
	qFU-C22-3	2014Yc	40.31			-0.57		A	MARBCF$_1$
	qFU-C23-1	2015Bg	0.01			-1.10	2.39	OD	MARBCF$_1$
		2015Bg	0.01		-1.32				MARBCF$_1$MPH
	qFU-C26-1	2015Bg	1.01		-0.59			OD	MARBCF$_1$MPH
	qFU-C26-2	2014Bg	35.01			0.42		A	MARBCF$_1$
MIC	qMIC-C01-1	2015Bg	6.21			-0.22		A	MARBCF$_1$
	**qMIC-Chr01-1**	2014Yc	14.51	0.24					RIL
	qMIC-C02-1	2015Yc	35.81			0.73	0.92	PD	MARBCF$_1$
		2015Yc	35.81		0.33				MARBCF$_1$MPH
	qMIC-C02-2	2015Yc	44.51			-0.74		A	MARBCF$_1$
	**qMIC-Chr05-1**	2014Yc	12.61	-0.25					RIL
	qMIC-C05-1	2015Bg	44.11			0.42		A	MARBCF$_1$
		2014Yc	44.41			0.07			MARBCF$_1$
		2014Bg	47.21			0.08			MARBCF$_1$
	qMIC-C05-2	2014Bg	60.51		0.03			OD	MARBCF$_1$MPH
	**qMIC-Chr07-1**	2014Bg	59.31	0.10					RIL
	qMIC-C10-1	2014Yc	43.71			0.33		A	MARBCF$_1$
	**qMIC-Chr10-1**	2014Yc	62.61	0.16					RIL
	qMIC-C13-1	2015Yc	7.41			0.29		A	MARBCF$_1$
	**qMIC-Chr14-1**	2014Yc	23.31	0.08					RIL
		2014Bg	20.91	0.11					RIL
	qMIC-C14-2	2014Yc	25.71			0.13		A	MARBCF$_1$
		2015Bg	30.51			0.27			MARBCF$_1$
	qMIC-C14-3	2015Bg	40.21			0.19		A	MARBCF$_1$
		2015Yc	41.01			0.10			MARBCF$_1$
	qMIC-C16-2	2015Bg	48.91			0.46		A	MARBCF$_1$
		2014Bg	51.61			0.13			MARBCF$_1$
	**qMIC-Chr16-1**	2014Bg	49.31	0.09					RIL
		2014Yc	51.01	0.08					RIL
	**qMIC-Chr16-2**	2014Yc	57.01	0.09					RIL
		2014Bg	57.41	0.15					RIL
	qMIC-C16-3	2014Bg	61.31			0.19		A	MARBCF$_1$

	**qMIC-Chr17-1**	2014Bg	44.81	0.12					RIL
	qMIC-C19-1	2015Bg	26.51			1.91		A	MARBCF_1
	qMIC-C19-2	2014Yc	35.81		-0.15			OD	MARBCF_1MPH
	qMIC-C24-1	2014Bg	13.71			-0.21		A	MARBCF_1
	**qMIC-Chr24-1**	2014Bg	16.81	-0.11					RIL
	**qMIC-Chr24-2**	2014Bg	73.31	0.44					RIL
FE	qFE-C01-1	2015Yc	11.71			0.16		A	MARBCF_1
	qFE-C05-1	2015Yc	58.91			0.07		A	MARBCF_1
		2015Bg	58.91			0.05			MARBCF_1
	qFE-C06-1	2014Yc	8.81			-0.46		A	MARBCF_1
	qFE-C09-1	2015Yc	20.51		-0.03			OD	MARBCF_1MPH
	qFE-C09-2	2015Bg	25.01			0.39		A	MARBCF_1
	qFE-C09-4	2014Yc	57.61		0.47			OD	MARBCF_1MPH
	qFE-C10-1	2015Bg	38.01			-0.08	-2.33	OD	MARBCF_1
		2015Bg	38.01		0.09				MARBCF_1MPH
	**qFE-Chr11-1**	2014Bg	5.31	1.16					RIL
	qFE-C13-1	2014Bg	33.41			-0.13		A	MARBCF_1
	**qFE-Chr14-2**	2014Bg	1.11	1.09					RIL
	qFE-C14-1	2015Bg	5.31			-0.36	2.68	OD	MARBCF_1
		2015Bg	5.31		-0.48				MARBCF_1MPH
	**qFE-Chr14-3**	2014Bg	6.31	0.25					RIL
	qFE-C14-2	2014Yc	9.51			0.22		A	MARBCF_1
	qFE-C14-3	2014Yc	15.81			0.32		A	MARBCF_1
	**qFE-Chr14-1**	2014Yc	16.81	0.30					RIL
		2014Bg	15.71	0.26					RIL
	qFE-C14-4	2014Yc	22.11			-0.07		A	MARBCF_1
	qFE-C14-5	2015Bg	45.01		-0.11			OD	MARBCF_1MPH
	qFE-C15-1	2015Yc	25.31		0.06			OD	MARBCF_1MPH
	**qFE-Chr16-1**	2014Bg	1.11	1.17					RIL
	qFE-C16-1	2015Bg	4.31			0.04		A	MARBCF_1
	**qFE-Chr17-1**	2014Bg	42.31	0.18					RIL
	qFE-C19-1	2015Yc	51.51			0.11		A	MARBCF_1
	**qFE- Chr18-1**	2014Bg	57.51	0.76					RIL
	**qFE-Chr20-1**	2014Yc	41.51	0.20					RIL
		2014Bg	47.11	0.44					RIL
	qFE-C21-1	2015Bg	9.91			-0.04	-4.93	OD	MARBCF_1
		2015Yc	10.91		0.10				MARBCF_1MPH
	qFE-C22-1	2015Bg	10.71			0.03		A	MARBCF_1
	qFE-C24-1	2014Bg	27.01			0.22		A	MARBCF_1
	qFE-C24-2	2014Bg	38.11		-0.28			OD	MARBCF_1MPH
	**qFE-Chr24-1**	2014Yc	73.31	0.75					RIL
		2014Bg	73.31	0.62					RIL
	qFE-C26-1	2015Bg	50.21			-0.02	29.56	OD	MARBCF_1
		2015Bg	51.31		-0.35				MARBCF_1MPH

FS	qFS-C03-1	2014Yc	78.81			-0.22	11.05	OD	MARBCF$_1$
		2014Yc	78.81		-1.19				MARBCF$_1$MPH
	**qFS-Chr05-1**	2014Bg	54.81	0.52					RIL
	qFS-C07-1	2015Bg	21.91		0.06			OD	MARBCF$_1$MPH
		2015Yc	23.21		1.46				MARBCF$_1$MPH
	qFS-C07-2	2014Yc	43.11			2.59		A	MARBCF$_1$
	qFS-C09-3	2015Yc	59.01		0.98			OD	MARBCF$_1$MPH
	qFS-C13-1	2015Bg	20.41			0.19		A	MARBCF$_1$
	qFS-C13-2	2015Yc	32.41		0.61			OD	MARBCF$_1$MPH
	**qFS-Chr14-1**	2014Yc	6.71	-0.21					RIL
	**qFS-Chr14-2**	2014Yc	14.21	-0.28					RIL
	**qFS-Chr14-3**	2014Yc	21.61	-0.26					RIL
	**qFS-Chr14-4**	2014Yc	40.11	-0.20					RIL
	qFS-C16-1	2014Bg	45.61			-1.48		A	MARBCF$_1$
	qFS-C17-1	2014Yc	31.01			0.75		A	MARBCF$_1$
	qFS-C18-1	2014Yc	20.11		1.87			OD	MARBCF$_1$MPH
	**qFS-Chr19-1**	2014Bg	22.51	0.51					RIL
	**qFS-Chr19-2**	2014Bg	27.81	0.61					RIL
	qFS-C19-2	2014Bg	52.21			0.87		A	MARBCF$_1$
	**qFS-Chr20-1**	2014Yc	42.11	-0.50					RIL
	**qFS-Chr20-2**	2014Yc	59.61	-0.48					RIL
	qFS-C20-2	2014Bg	68.61			-2.14		A	MARBCF$_1$
	qFS-C24-1	2015Yc	59.01		1.23			OD	MARBCF$_1$MPH
		2015Bg	60.61		-0.83				MARBCF$_1$MPH

[a] FL: fiber length; FU: fiber uniformity; MIC: micronaire; FE: fiber elongation; FS: fiber strength

[b] QTLs in bold are those identified by CIM in RILs in our previous study (Li et al. 2016), which was just used to estimate the gene action of MARBCF$_1$ population

[c] 2014Yc: Yacheng of Hainan Province in 2014; 2014Bg: Baogang of Hainan Province in 2014; 2015Yc: Yacheng of Hainan Province in 2015; 2015Bg: Baogang of Hainan Province in 2015

[d] Position of QTL located on chromosome: as cM distance from the top of each chromosome

[e] The genetic expectation of a QTL effect obtained is the additive effect (A) from the RILs, the additive and dominance effects (A+D) from the BCF$_1$s, and the dominance effect (D) from the MPH values

[f] |D/A|: |dominance/additive|; 2|D|/|(A+D)|: 2| dominance |/|(additive + dominance)|

[g] GA: gene action; PD/D partial dominance (|d/a| <= 1 or 2|d|/|(a+d)| <= 1); OD overdominance(|d/a| >1 or 2|d|/|(a+d)| > 1), here, 2|d|/|(a+d)| > 1 same to 2|d| > |a + d|; A: when QTL detected only in BCF$_1$ or both BCF$_1$ and RIL was referred to as additive (A).
