# Supplementary material for: QTL Mapping and Heterosis Analysis for Fiber Quality Traits Across Multiple Genetic Populations and Environments in Upland Cotton
Source: Front Plant Sci. 2018 Oct 15;9:1364. doi: 10.3389/fpls.2018.01364 (PMC6196769; doi:10.3389/fpls.2018.01364)
Supplement: Supplementary file 7 [file Data_Sheet_7.PDF]

Table S7 Main effects and environmental interactions detected for fiber quality traits in IF_2 and two BCF_1 datasets by ICIM method

Traits[a]	m-QTL	Chr.	Position[b]	Flanking markers[c]	LOD[d]	PV[e]	PV(A)[e]	PV(AE)[e]
				**IF_2s**				
FL	ImaqFL-C01-1	1	37	i02297Gh-i48068Gh	4.49	2.08	0.82	1.26
	ImaqFL-C05-1	5	13	i46433Gh-i26271Gh	4.63	2.06	0.19	1.87
	ImaqFL-C09-1	9	90	i13502Gh-i25039Gh	5.32	2.36	2.00	0.36
	ImaqFL-C13-1	13	31	i32083Gh-i36415Gh	4.86	2.11	1.49	0.62
	ImaqFL-C16-1	16	57	i44137Gh-i48344Gh	4.55	2.21	1.24	0.97
	ImaqFL-C18-1	18	45	i39369Gh-i13456Gh	4.60	2.07	1.81	0.26
	ImaqFL-C18-2	18	53	i13472Gh-i35011Gh	4.81	2.14	1.92	0.22
	ImaqFL-C20-1	20	11	**i51628Gb-i36952Gh**	5.19	2.67	1.28	1.39
	ImaqFL-C20-2	20	31	i11735Gh-i44450Gh	4.87	1.90	0.48	1.42
	ImaqFL-C20-3	20	41	i26441Gh-i11912Gh	4.57	1.85	0.36	1.50
	ImaqFL-C20-4	20	59	i40369Gh-i11915Gh	6.74	2.78	0.79	1.99
FU	ImaqFU-C03-1	3	63	i49177Gh-i39896Gh	4.82	3.88	0.45	3.43
	ImaqFU-C20-1	20	31	i11735Gh-i44450Gh	7.08	4.29	3.55	0.74
	ImaqFU-C25-1	25	42	i22495Gh-i55440Gb	4.51	3.28	0.62	2.66
MIC	ImaqMIC-C01-1	1	0	i33646Gh-i40884Gh	4.62	2.22	1.15	1.07
	ImaqMIC-C01-2	1	7	i31143Gh-i48104Gh	5.40	2.52	1.44	1.08
	ImaqMIC-C01-3	1	24	i30614Gh-i53496Gb	5.26	2.29	1.10	1.18
	ImaqMIC-C05-1	5	48	i09003Gh-i45318Gh	4.43	1.91	1.05	0.86
	ImaqMIC-C13-1	13	34	i37089Gh-i41278Gh	5.35	2.25	0.06	2.19
	ImaqMIC-C16-1	16	8	i13939Gh-i01279Gh	4.97	1.99	0.85	1.14
	ImaqMIC-C17-1	17	57	i03688Gh-i13994Gh	5.51	1.81	1.75	0.05
	ImaqMIC-C18-1	18	6	i13145Gh-i29829Gh	4.45	1.54	0.79	0.74
	ImaqMIC-C19-1	19	11	i28797Gh-i09073Gh	5.56	2.39	0.99	1.40
	ImaqMIC-C24-1	24	60	i03705Gh-i33113Gh	6.81	2.43	0.19	2.24
	ImaqMIC-C25-1	25	52	i11287Gh-i17145Gh	4.55	1.79	0.15	1.64
FE	ImaqFE-C02-1	2	0	i17680Gh-i02755Gh	4.52	2.73	1.18	1.56
	ImaqFE-C02-2	2	58	i39651Gh-i02432Gh	4.68	3.77	1.66	2.11
	ImaqFE-C07-1	7	64	i14398Gh-i01824Gh	4.49	1.12	0.23	0.90
	ImaqFE-C13-1	13	31	i32083Gh-i36415Gh	6.30	6.81	3.10	3.71
	ImaqFE-C16-1	16	41	**i45950Gh-i36953Gh**	4.89	4.55	2.09	2.46
	ImaqFE-C18-1	18	35	i32883Gh-i13851Gh	6.07	2.81	0.94	1.87
FS	ImaqFS-C08-1	8	31	i42167Gh-i62711Gt	4.57	1.95	0.37	1.58
	ImaqFS-C11-1	11	41	i43181Gh-i16165Gh	4.48	2.32	1.62	0.70
	ImaqFS-C14-1	14	70	i38712Gh-i23682Gh	4.73	2.61	0.99	1.62
	ImaqFS-C16-1	16	57	i44137Gh-i48344Gh	4.42	2.23	1.11	1.11
	ImaqFS-C16-2	16	61	i58144Gb-i54645Gb	4.91	2.57	2.25	0.32
				**$HSBCF_1$s**				
FL	B_1maqFL-C01-1	1	39	i55243Gb-i02295Gh	5.24	3.26	2.44	0.82
	B_1maqFL-C08-1	8	15	i04570Gh-i04506Gh	4.70	2.60	1.94	0.65
	B_1maqFL-C09-1	9	34	i36007Gh-i62860Gt	4.62	2.57	1.92	0.64

FU	B_1maqFU-C01-1	1	39	**i55243Gb-i02295Gh**	5.25	3.14	2.35	0.79
	B_1maqFU-C08-1	8	15	i04570Gh-i04506Gh	4.02	2.42	1.80	0.62
	B_1maqFU-C091	9	34	i36007Gh-i62860Gt	4.62	2.47	1.85	0.62
MIC	B_1maqMIC-C01-1	1	39	i55243Gb-i02295Gh	5.26	3.52	2.64	0.88
	B_1maqMIC-C08-1	8	15	i04570Gh-i04506Gh	4.02	2.75	2.04	0.71
	B_1maqMIC-C09-1	9	34	i36007Gh-i62860Gt	4.63	2.77	2.08	0.69
FE	B_1maqFE-C01-1	1	39	**i55243Gb-i02295Gh**	5.27	3.41	2.56	0.85
	B_1maqFE-C04-1	4	31	i36496Gh-i46763Gh	4.10	1.11	0.80	0.31
	B_1maqFE-C08-1	8	15	i04570Gh-i04506Gh	4.07	2.66	1.98	0.68
	B_1maqFE-C09-1	9	34	i36007Gh-i62860Gt	4.62	2.68	2.01	0.67
FS	B_1maqFS-C01-1	1	39	i55243Gb-i02295Gh	5.25	2.89	2.16	0.73
	B_1maqFS-C08-1	8	15	i04570Gh-i04506Gh	4.25	2.34	1.75	0.59
	B_1maqFS-C09-1	9	34	i36007Gh-i62860Gt	4.62	2.29	1.72	0.58
				**MARBCF$_1$s**				
FL	B_2maqFL-C09-1	9	6	i25689Gh-i17373Gh	4.12	1.79	1.50	0.29
	B_2maqFL-C09-2	9	56	i08720Gh-i15489Gh	4.93	2.78	2.52	0.27
	B_2maqFL-C14-1	14	21	i05024Gh-i00465Gh	5.47	3.46	3.01	0.46
	B_2maqFL-C17-1	17	46	i42547Gh-i14844Gh	5.47	3.02	2.80	0.21
	B_2maqFL-C18-1	18	5	i13754Gh-i13145Gh	4.28	2.06	1.85	0.21
	B_2maqFL-C18-2	18	119	i13081Gh-i13709Gh	4.17	2.76	2.15	0.61
	B_2maqFL-C19-1	19	26	i27871Gh-i09035Gh	5.35	3.25	2.83	0.42
	B_2maqFL-C20-1	20	41	i26441Gh-i11912Gh	4.66	3.12	2.20	0.92
FU	B_2maqFU-C14-1	14	38	i41064Gh-i28364Gh	4.11	6.05	3.36	2.69
	B_2maqFU-C24-1	24	44	**i26213Gh-i00339Gh**	4.46	8.53	3.54	4.99
MIC	B_2maqMIC-C07-1	7	53	i50051Gb-i01629Gh	4.55	1.15	0.89	0.26
	B_2maqMIC-C09-1	9	38	i06090Gh-i52130Gb	4.27	0.97	0.64	0.33
	B_2maqMIC-C13-1	13	4	i35121Gh-i24988Gh	5.33	1.55	1.50	0.05
	B_2maqMIC-C14-1	14	11	**i46775Gh-i43468Gh**	5.01	1.27	0.97	0.31
	B_2maqMIC-C14-2	14	25	i15375Gh-i05040Gh	4.09	1.43	1.28	0.15
	B_2maqMIC-C17-1	17	45	i00956Gh-i42547Gh	8.61	2.78	2.69	0.08
	B_2maqMIC-C23-1	23	30	i26750Gh-i31332Gh	4.49	1.46	1.39	0.07
	B_2maqMIC-C24-1	24	17	i15167Gh-i41753Gh	4.43	1.85	1.12	0.73
FE	B_2maqFE-C04-1	4	34	i46763Gh-i10499Gh	7.09	19.16	8.62	10.54
	B_2maqFE-C06-1	6	9	i06061Gh-i05824Gh	4.41	5.72	1.46	4.26
	B_2maqFE-C07-1	7	22	i57601Gb-i01453Gh	5.37	6.22	1.80	4.42
	B_2maqFE-C09-1	9	36	i39433Gh-i06090Gh	5.38	1.10	0.03	1.07
	B_2maqFE-C14-1	14	18	i21369Gh-i04874Gh	6.29	16.53	8.01	8.51
	B_2maqFE-C18-1	18	119	i13081Gh-i13709Gh	4.40	7.49	1.88	5.61
	B_2maqFE-C24-1	24	25	i18607Gh-i33719Gh	5.06	0.83	0.09	0.74
FS	B_2maqFS-C07-1	7	51	i01611Gh-i27926Gh	4.18	3.08	2.88	0.20
	B_2maqFS-C19-1	19	52	i08832Gh-i09452Gh	4.67	3.47	3.25	0.22

[a] FL: fiber length; FU: fiber uniformity; MIC: micronaire; FE: fiber elongation; FS: fiber strength

[b] Position of QTL located on chromosome: as cM distance from the top of each chromosome

[c] Flanking markers in bold are those flanking m-QTLs identified again in e-QTLS by ICIM in additional Table S9

[d] A LOD threshold was used for declaration of QTL based on 1000 permutations at as significance level of 0.01

[e] PV: the phenotypic variance that the total additive effect explained; PV(A): the phenotypic variation that the main additive effect explained; PV(AE): the phenotypic variation that the environmental additive effect explained
