# Supplementary material for: QTL Mapping and Heterosis Analysis for Fiber Quality Traits Across Multiple Genetic Populations and Environments in Upland Cotton
Source: Front Plant Sci. 2018 Oct 15;9:1364. doi: 10.3389/fpls.2018.01364 (PMC6196769; doi:10.3389/fpls.2018.01364)
Supplement: Supplementary file 8 [file Data_Sheet_8.PDF]

Table S8 Main effects and environmental interactions detected for fiber quality traits in IF_2MPH, $HSBCF_1$MPH, and $MARBCF_1$MPH datasets by ICIM method

Traits[a]	m-QTL	Chr.	Position[b]	Flanking markers[c]	LOD[d]	PV[e]	PV(A)[e]	PV(AE)[e]
				**IF_2MPHs**				
FL	IMmaqFL-C04-1	4	32	i36496Gh-i46763Gh	4.48	1.90	1.29	0.61
	IMmaqFL-C16-1	16	57	i44137Gh-i48344Gh	5.32	2.86	1.48	1.38
	IMmaqFL-C25-1	25	1	i00577Gh-i17365Gh	4.63	2.27	0.97	1.30
FU	IMmaqFU-C03-1	3	69	i39896Gh-i38338Gh	4.40	4.42	0.70	3.72
	IMmaqFU-C14-1	14	45	i27231Gh-i36385Gh	5.05	3.14	0.57	2.57
	IMmaqFU-C25-1	25	42	i22495Gh-i55440Gb	4.54	3.83	0.64	3.19
MIC	IMmaqMIC-C01-1	1	7	i31143Gh-i48104Gh	4.45	2.61	2.23	0.39
	IMmaqMIC-C05-1	5	7	i23461Gh-i43323Gh	4.75	1.81	0.59	1.21
FE	IMmaqFE-C03-1	3	85	i31859Gh-i42939Gh	4.41	1.28	0.30	0.97
	IMmaqFE-C11-1	11	33	i20872Gh-i40251Gh	4.77	2.21	0.85	1.36
FS	IMmaqFS-C08-1	8	32	i62711Gt-i04772Gh	5.23	1.57	0.09	1.48
	IMmaqFS-C24-1	24	12	i32211Gh-i25139Gh	4.52	2.32	1.84	0.48
				**$HSBCF_1$MPHs**				
FL	B_1MmaqFL-C01-1	1	17	i46188Gh-i23810Gh	4.07	3.70	2.65	1.04
	B_1MmaqFL-C09-1	9	38	i06090Gh-i52130Gb	4.29	3.81	2.88	0.93
	B_1MmaqFL-C10-1	10	0	i43940Gh-i25267Gh	7.90	6.91	5.18	1.73
	B_1MmaqFL-C21-1	21	58	i07219Gh-i49145Gh	5.15	4.33	3.26	1.07
MIC	B_1MmaqMIC-C09-1	9	63	i11844Gh-i14723Gh	4.11	6.15	3.84	2.31
FS	B_1MmaqFS-C14-1	14	63	i21263Gh-i15610Gh	4.67	2.90	2.63	0.27
				**$MARBCF_1$MPHs**				
FL	B_2MmaqFL-C21-1	21	24	i22367Gh-i47711Gh	4.72	3.41	3.22	0.20
FU	B_2MmaqFU-C09-1	9	22	i25759Gh-i03659Gh	4.01	10.14	0.88	9.27
MIC	B_2MmaqMIC-C19-1	19	30	i16566Gh-i08941Gh	4.11	2.96	0.38	2.58
FE	B_2MmaqFE-C09-1	9	36	i39433Gh-i06090Gh	6.05	3.04	0.03	3.01
	B_2MmaqFE-C16-1	16	22	i46747Gh-i01444Gh	7.69	1.95	0.00	1.95
	B_2MmaqFE-C18-1	18	27	i20346Gh-i13454Gh	4.47	5.66	0.83	4.83
FS	B_2MmaqFS-C24-1	24	16	i04568Gh-i25656Gh	4.50	5.57	3.73	1.84

[a] FL: fiber length; FU: fiber uniformity; MIC: micronaire; FE: fiber elongation; FS: fiber strength

[b] Position of QTL located on chromosome: as cM distance from the top of each chromosome

[c] Flanking markers in bold are those flanking m-QTLs identified again in e-QTLS by ICIM in additional Table S10

[d] A LOD threshold was used for declaration of QTL based on 1000 permutations at as significance level of 0.01

[e] PV: the phenotypic variance that the total additive and dominance effects explained; PV (A): phenotypic variance explained by main additive and dominance effects; PV (AE): phenotypic variance explained by environmental additive and dominance effects
