# Supplementary material for: QTL Mapping and Heterosis Analysis for Fiber Quality Traits Across Multiple Genetic Populations and Environments in Upland Cotton
Source: Front Plant Sci. 2018 Oct 15;9:1364. doi: 10.3389/fpls.2018.01364 (PMC6196769; doi:10.3389/fpls.2018.01364)
Supplement: Supplementary file 9 [file Data_Sheet_9.PDF]

Table S9 Epistatic effects and environmental interactions detected for fiber quality traits in IF_2 and two BCF_1 datasets by ICIM method

Traits[a]	e-QTL	Type of epistasis[b]	Chr.i[c]	Position1[d]	Flanking markers 1[e]	Chr.j[c]	Position2[d]	Flanking markers 2[e]	LOD[f]	PV[g]	PV(AA)[g]	PV(AAE)[g]
						IF_2s						
FL	Imeq-FL-1	III	1	5	i21210Gh-i35065Gh	4	0	i50068Gb-i26515Gh	8.71	4.64	1.57	3.07
	Imeq-FL-2	III	3	85	i31859Gh-i42939Gh	5	40	i08988Gh-i45534Gh	13.70	0.44	0.19	0.25
	Imeq-FL-3	III	5	40	i08988Gh-i45534Gh	7	35	i42900Gh-i21721Gh	8.31	2.38	0.06	2.33
	Imeq-FL-4	III	6	10	i06061Gh-i05824Gh	11	0	i52789Gb-i07420Gh	8.45	4.33	3.11	1.22
	Imeq-FL-5	III	7	10	i24917Gh-i26814Gh	13	35	i37089Gh-i41278Gh	8.29	3.97	0.55	3.42
	Imeq-FL-6	III	11	0	i52789Gb-i07420Gh	15	0	i02955Gh-i02314Gh	8.46	4.96	1.18	3.79
	Imeq-FL-7	III	1	5	i21210Gh-i35065Gh	17	15	i03341Gh-i28038Gh	8.05	3.57	1.46	2.11
	Imeq-FL-8	III	7	10	i24917Gh-i26814Gh	17	30	i14920Gh-i51624Gb	8.90	3.97	2.77	1.20
	Imeq-FL-9	III	6	40	i06505Gh-i23722Gh	18	30	i32883Gh-i13851Gh	8.57	3.41	1.66	1.76
	Imeq-FL-10	II	3	65	i49177Gh-i39896Gh	20	10	**i39228Gh-i34769Gh**	8.56	3.59	1.48	2.10
	Imeq-FL-11	III	19	40	i08786Gh-i00558Gh	20	80	i11915Gh-i11478Gh	8.77	3.90	3.55	0.35
	Imeq-FL-12	III	13	35	i37089Gh-i41278Gh	21	5	i07547Gh-i20988Gh	8.59	4.46	1.65	2.81
	Imeq-FL-13	III	12	15	i40974Gh-i48211Gh	22	45	i44682Gh-i25111Gh	8.63	3.96	2.58	1.38
	Imeq-FL-14	III	1	5	i21210Gh-i35065Gh	24	55	i14999Gh-i14993Gh	8.20	4.00	2.02	1.98
	Imeq-FL-15	III	5	55	i09147Gh-i42098Gh	24	55	i14999Gh-i14993Gh	8.08	4.13	2.55	1.58
	Imeq-FL-16	III	11	10	i47563Gh-i01036Gh	24	55	i14999Gh-i14993Gh	10.40	3.91	2.17	1.74
	Imeq-FL-17	III	13	40	i46668Gh-i00187Gh	24	55	i14999Gh-i14993Gh	9.67	3.58	0.82	2.76
	Imeq-FL-18	III	18	70	i49258Gh-i13532Gh	25	50	i40453Gh-i46187Gh	10.38	5.49	0.87	4.62
	Imeq-FL-19	III	21	50	i35971Gh-i47631Gh	26	5	i08062Gh-i33827Gh	8.36	4.40	2.35	2.05
	Imeq-FL-20	III	22	45	i44682Gh-i25111Gh	26	5	i08062Gh-i33827Gh	8.70	4.55	2.66	1.89
FU	Imeq-FU-1	III	3	10	i32361Gh-i00583Gh	14	25	i15375Gh-i05040Gh	8.07	3.54	1.45	2.10
	Imeq-FU-2	III	10	0	i43940Gh-i25267Gh	14	25	i15375Gh-i05040Gh	9.96	4.87	2.55	2.32
	Imeq-FU-3	III	11	40	i43181Gh-i16165Gh	14	45	i27231Gh-i36385Gh	8.08	3.85	2.66	1.20

	Imeq-FU-4	III	8	30	i54149Gb-i00217Gh	15	25	i18410Gh-i38002Gh	8.18	3.89	1.57	2.32
	Imeq-FU-5	III	17	45	i00956Gh-i42547Gh	18	70	i49258Gh-i13532Gh	8.33	3.94	2.74	1.20
	Imeq-FU-6	III	20	5	i00478Gh-i11539Gh	21	25	i22367Gh-i47711Gh	8.54	3.36	2.09	1.27
	Imeq-FU-7	III	19	20	i08987Gh-i09220Gh	21	60	i22642Gh-i41613Gh	8.43	4.09	1.65	2.43
	Imeq-FU-8	III	20	40	i37554Gh-i47006Gh	24	10	i04567Gh-i15176Gh	9.03	4.21	0.91	3.29
	Imeq-FU-9	III	19	20	i08987Gh-i09220Gh	24	20	i36485Gh-i41754Gh	8.19	3.62	1.67	1.95
	Imeq-FU-10	III	14	25	i15375Gh-i05040Gh	24	40	i48423Gh-i43942Gh	9.20	3.81	2.16	1.65
	Imeq-FU-11	III	18	50	i13451Gh-i38577Gh	24	45	i26213Gh-i00339Gh	8.96	3.25	1.64	1.61
	Imeq-FU-12	III	15	25	i18410Gh-i38002Gh	26	5	i08062Gh-i33827Gh	8.26	3.99	2.34	1.65
	Imeq-FU-13	III	20	25	i25398Gh-i11734Gh	26	10	i33827Gh-i25834Gh	8.84	3.71	2.33	1.38
	Imeq-FU-14	III	25	50	i40453Gh-i46187Gh	26	20	i37251Gh-i23249Gh	8.26	3.80	2.49	1.31
MIC	Imeq-MIC-1	III	15	5	i02306Gh-i02317Gh	18	70	i49258Gh-i13532Gh	9.14	4.60	2.21	2.39
FE	Imeq-FE-1	III	7	40	i34772Gh-i43291Gh	8	15	i04570Gh-i04506Gh	8.77	4.72	2.33	2.39
	Imeq-FE-2	III	8	15	i04570Gh-i04506Gh	10	35	i38146Gh-i12097Gh	9.17	0.61	0.23	0.38
	Imeq-FE-3	III	9	10	i05758Gh-i19700Gh	11	15	i01036Gh-i07468Gh	9.90	2.42	1.06	1.36
	Imeq-FE-4	III	2	90	i07717Gh-i09654Gh	11	25	i07163Gh-i56975Gb	12.74	0.18	0.10	0.08
	Imeq-FE-5	III	7	55	i01629Gh-i14398Gh	11	25	i07163Gh-i56975Gb	12.92	0.93	0.45	0.48
	Imeq-FE-6	III	1	50	i31438Gh-i26341Gh	12	40	i48211Gh-i08075Gh	8.22	0.07	0.03	0.04
	Imeq-FE-7	III	13	5	i37798Gh-i22760Gh	13	65	i29310Gh-i00241Gh	14.85	0.55	0.50	0.04
	Imeq-FE-8	III	3	115	i05514Gh-i29314Gh	14	60	i26838Gh-i01129Gh	12.40	0.53	0.21	0.33
	Imeq-FE-9	III	3	35	i27670Gh-i40392Gh	15	5	i02306Gh-i02317Gh	12.52	0.19	0.02	0.17
	Imeq-FE-10	III	14	40	i22707Gh-i38937Gh	16	45	i54957Gb-i59324Gb	12.00	0.84	0.31	0.52
	Imeq-FE-11	II	16	40	**i45950Gh-i36953Gh**	17	35	i03218Gh-i03216Gh	9.28	1.65	0.52	1.13
	Imeq-FE-12	III	11	15	i01036Gh-i07468Gh	18	85	i37364Gh-i31825Gh	9.22	1.04	0.17	0.87
	Imeq-FE-13	III	4	5	i26515Gh-i43091Gh	19	35	i50235Gb-i52709Gb	9.36	0.72	0.18	0.54
	Imeq-FE-14	III	16	15	i14406Gh-i01766Gh	19	55	i17039Gh-i16573Gh	11.03	0.02	0.02	0.00

	Imeq-FE-15	III	9	35	i03637Gh-i11677Gh	20	55	i12251Gh-i11715Gh	9.37	0.06	0.01	0.05
	Imeq-FE-16	III	13	35	i37089Gh-i41278Gh	22	15	i43141Gh-i60448Gb	11.23	0.29	0.08	0.21
	Imeq-FE-17	III	5	40	i08988Gh-i45534Gh	22	45	i44682Gh-i25111Gh	14.18	6.06	2.92	3.15
	Imeq-FE-18	III	9	90	i13502Gh-i25039Gh	23	30	i26750Gh-i31332Gh	14.94	0.01	0.01	0.00
FS	Imeq-FS-1	III	1	5	i21210Gh-i35065Gh	3	75	i43226Gh-i45963Gh	8.51	1.34	1.13	3.29
	Imeq-FS-2	III	4	0	i50068Gb-i26515Gh	6	20	i22608Gh-i06067Gh	8.55	3.93	2.67	1.26
	Imeq-FS-3	III	3	65	i49177Gh-i39896Gh	11	35	i40251Gh-i07190Gh	9.22	4.32	3.53	0.78
	Imeq-FS-4	III	9	5	i25689Gh-i17373Gh	11	35	i40251Gh-i07190Gh	9.86	4.66	3.28	1.39
	Imeq-FS-5	III	9	70	i00393Gh-i04801Gh	18	15	i31442Gh-i13146Gh	8.39	4.85	2.06	2.78
	Imeq-FS-6	III	6	50	i37862Gh-i06396Gh	18	75	i13532Gh-i43889Gh	8.20	3.77	2.38	1.39
	Imeq-FS-7	III	12	0	i40974Gh-i48211Gh	19	20	i08987Gh-i09220Gh	8.29	3.52	1.83	1.69
	Imeq-FS-8	III	6	40	i06505Gh-i23722Gh	20	5	i00478Gh-i11539Gh	8.48	4.61	2.30	2.31
	Imeq-FS-9	III	13	35	i37089Gh-i41278Gh	20	10	i39228Gh-i34769Gh	8.41	4.03	3.02	1.01
	Imeq-FS-10	III	14	15	i15343Gh-i31037Gh	20	40	i37554Gh-i47006Gh	8.17	3.81	2.12	1.69
	Imeq-FS-11	III	4	15	i41085Gh-i38159Gh	21	50	i35971Gh-i47631Gh	8.77	4.72	2.15	2.56
	Imeq-FS-12	III	4	0	i50068Gb-i26515Gh	24	45	i26213Gh-i00339Gh	8.25	4.48	1.49	2.98
	Imeq-FS-13	III	5	55	i09147Gh-i42098Gh	25	40	i22495Gh-i55440Gb	8.61	3.32	1.98	1.34
	Imeq-FS-14	III	15	0	i02955Gh-i02314Gh	25	40	i22495Gh-i55440Gb	8.14	4.76	1.29	3.47
	Imeq-FS-15	III	23	0	i06287Gh-i06171Gh	25	40	i22495Gh-i55440Gb	8.37	5.17	1.81	3.36
	Imeq-FS-16	III	14	25	i15375Gh-i05040Gh	25	50	i40453Gh-i46187Gh	8.69	4.14	1.72	2.42
	Imeq-FS-17	III	16	10	i13939Gh-i01279Gh	26	45	i22171Gh-i00945Gh	8.10	4.10	2.37	1.73
						**HSBCF_1s**						
FL	B_1meq-FL-1	III	2	45	i05624Gh-i02723Gh	19	35	i50235Gb-i52709Gb	8.16	3.02	2.21	0.81
	B_1meq-FL-2	III	2	55	i02246Gh-i00463Gh	24	15	i04568Gh-i25656Gh	9.54	2.36	1.78	0.59
	B_1meq-FL-3	III	3	105	i20709Gh-i23313Gh	13	15	i30934Gh-i18151Gh	10.73	3.47	2.54	0.93
	B_1meq-FL-4	III	4	5	i26515Gh-i43091Gh	15	30	i02459Gh-i02486Gh	9.42	3.59	2.60	0.99

	B_1meq-FL-5	III	5	10	i38565Gh-i36865Gh	5	55	i09147Gh-i42098Gh	9.24	2.62	2.05	0.57
	B_1meq-FL-6	III	5	10	i38565Gh-i36865Gh	7	0	i32739Gh-i37773Gh	8.09	2.77	2.09	0.69
	B_1meq-FL-7	III	5	10	i38565Gh-i36865Gh	18	70	i49258Gh-i13532Gh	9.53	2.19	1.85	0.34
	B_1meq-FL-8	III	5	20	i35761Gh-i09052Gh	11	40	i43181Gh-i16165Gh	9.72	3.45	2.59	0.86
	B_1meq-FL-9	III	5	50	i16666Gh-i09095Gh	9	10	i05758Gh-i19700Gh	8.62	3.11	2.31	0.80
	B_1meq-FL-10	III	6	25	i26917Gh-i06526Gh	22	0	i00456Gh-i12928Gh	10.67	2.94	2.12	0.81
	B_1meq-FL-11	III	8	0	i63682Gm-i37825Gh	8	50	i01126Gh-i04719Gh	8.44	3.00	2.21	0.79
	B_1meq-FL-12	III	8	5	i31145Gh-i40270Gh	13	30	i24929Gh-i13848Gh	8.55	2.53	1.87	0.66
	B_1meq-FL-13	III	8	20	i25482Gh-i25868Gh	15	0	i02955Gh-i02314Gh	8.50	3.30	2.36	0.94
	B_1meq-FL-14	III	9	40	i10438Gh-i08573Gh	22	0	i00456Gh-i12928Gh	8.55	3.04	2.24	0.80
	B_1meq-FL-15	III	9	65	i49158Gh-i00393Gh	18	85	i37364Gh-i31825Gh	8.79	2.37	1.80	0.57
	B_1meq-FL-16	III	11	40	i43181Gh-i16165Gh	19	25	i36086Gh-i27871Gh	10.59	2.94	2.23	0.72
	B_1meq-FL-17	III	14	40	i22707Gh-i38937Gh	19	30	i16566Gh-i08941Gh	10.22	3.58	2.62	0.96
	B_1meq-FL-18	III	14	50	i34963Gh-i44045Gh	16	50	i46435Gh-i58367Gb	10.16	3.02	2.20	0.82
	B_1meq-FL-19	III	18	55	i13319Gh-i42821Gh	24	20	i36485Gh-i41754Gh	9.80	3.08	2.19	0.89
	B_1meq-FL-20	III	19	20	i08987Gh-i09220Gh	25	30	i11464Gh-i46788Gh	9.00	2.20	1.65	0.55
	B_1meq-FL-21	III	20	45	i17500Gh-i47439Gh	21	40	i07558Gh-i07515Gh	10.94	2.84	2.11	0.73
	B_1meq-FL-22	III	20	60	i11915Gh-i11478Gh	21	60	i22642Gh-i41613Gh	10.69	3.85	2.87	0.98
	B_1meq-FL-23	III	24	40	i48423Gh-i43942Gh	26	20	i37251Gh-i23249Gh	10.45	3.37	2.51	0.86
	B_1meq-FL-24	III	24	55	i14999Gh-i14993Gh	25	40	i22495Gh-i55440Gb	9.40	3.27	2.45	0.82
FU	B_1meq-FU-1	II	1	40	**i21520Gh-i14552Gh**	20	35	i40942Gh-i35292Gh	9.33	2.12	1.57	0.55
	B_1meq-FU-2	III	2	55	i02246Gh-i00463Gh	24	15	i04568Gh-i25656Gh	9.57	2.36	1.74	0.61
	B_1meq-FU-3	III	5	10	i38565Gh-i36865Gh	5	55	i09147Gh-i42098Gh	9.22	2.62	2.05	0.57
	B_1meq-FU-4	III	5	10	i38565Gh-i36865Gh	18	70	i49258Gh-i13532Gh	9.53	2.20	1.85	0.35
	B_1meq-FU-5	III	5	45	i29825Gh-i01144Gh	22	30	i12906Gh-i17853Gh	9.02	2.67	1.89	0.78
	B_1meq-FU-6	III	5	50	i16666Gh-i09095Gh	9	10	i05758Gh-i19700Gh	8.54	2.92	2.18	0.74

	B_1meq-FU-7	III	6	20	i22608Gh-i06067Gh	8	30	i54149Gb-i00217Gh	10.67	3.08	2.29	0.79
	B_1meq-FU-8	III	10	65	i22625Gh-i22107Gh	19	50	i08832Gh-i09452Gh	10.38	2.65	1.99	0.66
	B_1meq-FU-9	III	12	15	i40974Gh-i48211Gh	26	10	i33827Gh-i25834Gh	9.00	2.98	2.24	0.74
	B_1meq-FU-10	III	18	70	i49258Gh-i13532Gh	25	25	i27022Gh-i11449Gh	8.97	3.36	2.40	0.96
	B_1meq-FU-11	III	19	20	i08987Gh-i09220Gh	25	30	i11464Gh-i46788Gh	9.27	2.40	1.80	0.60
	B_1meq-FU-12	III	24	40	i48423Gh-i43942Gh	26	20	i37251Gh-i23249Gh	10.16	3.30	2.47	0.83
MIC	B_1meq-MIC-1	III	1	45	i02245Gh-i44115Gh	24	15	i04568Gh-i25656Gh	8.38	1.34	1.07	0.27
	B_1meq-MIC-2	III	2	45	i05624Gh-i02723Gh	19	35	i50235Gb-i52709Gb	8.02	2.89	2.10	0.79
	B_1meq-MIC-3	III	3	105	i20709Gh-i23313Gh	13	15	i30934Gh-i18151Gh	10.46	3.33	2.42	0.90
	B_1meq-MIC-4	III	4	5	i26515Gh-i43091Gh	22	40	i12810Gh-i17697Gh	9.25	2.85	2.12	0.74
	B_1meq-MIC-5	III	5	10	i38565Gh-i36865Gh	5	55	i09147Gh-i42098Gh	9.29	2.64	2.06	0.57
	B_1meq-MIC-6	III	5	45	i29825Gh-i01144Gh	22	30	i12906Gh-i17853Gh	8.92	2.61	1.87	0.74
	B_1meq-MIC-7	III	5	50	i16666Gh-i09095Gh	9	10	i05758Gh-i19700Gh	8.64	3.09	2.30	0.80
	B_1meq-MIC-8	III	7	20	i01696Gh-i57601Gb	25	30	i11464Gh-i46788Gh	8.18	1.89	1.42	0.47
	B_1meq-MIC-9	III	8	0	i63682Gm-i37825Gh	8	50	i01126Gh-i04719Gh	8.44	3.05	2.26	0.78
	B_1meq-MIC-10	III	8	20	i25482Gh-i25868Gh	15	0	i02955Gh-i02314Gh	8.45	3.26	2.32	0.94
	B_1meq-MIC-11	III	14	40	i22707Gh-i38937Gh	19	30	i16566Gh-i08941Gh	10.21	3.50	2.63	0.88
	B_1meq-MIC-12	III	15	5	i02306Gh-i02317Gh	24	40	i48423Gh-i43942Gh	10.61	3.70	2.75	0.95
	B_1meq-MIC-13	III	17	0	i14907Gh-i14878Gh	17	20	i03593Gh-i03537Gh	10.60	3.10	2.31	0.79
	B_1meq-MIC-14	III	18	55	i13319Gh-i42821Gh	24	20	i36485Gh-i41754Gh	9.68	3.08	2.20	0.88
	B_1meq-MIC-15	III	20	45	i17500Gh-i47439Gh	21	40	i07558Gh-i07515Gh	10.82	2.76	2.06	0.70
	B_1meq-MIC-16	III	22	40	i12810Gh-i17697Gh	26	5	i08062Gh-i33827Gh	10.44	3.43	2.57	0.86
FE	B_1meq-FE-1	II	1	40	**i21520Gh-i14552Gh**	20	35	i40942Gh-i35292Gh	9.92	2.32	1.73	0.60
	B_1meq-FE-2	III	4	5	i26515Gh-i43091Gh	15	30	i02459Gh-i02486Gh	9.50	3.63	2.65	0.99
	B_1meq-FE-3	III	8	0	i63682Gm-i37825Gh	8	50	i01126Gh-i04719Gh	8.42	3.00	2.24	0.76
	B_1meq-FE-4	III	9	40	i10438Gh-i08573Gh	22	0	i00456Gh-i12928Gh	8.43	2.84	2.08	0.76

	B_1meq-FE-5	III	9	75	i05825Gh-i14639Gh	26	5	i08062Gh-i33827Gh	8.75	3.26	2.44	0.82
	B_1meq-FE-6	III	10	65	i22625Gh-i22107Gh	16	50	i46435Gh-i58367Gb	10.66	4.20	3.11	1.09
	B_1meq-FE-7	III	10	65	i22625Gh-i22107Gh	19	50	i08832Gh-i09452Gh	10.22	2.56	1.93	0.63
	B_1meq-FE-8	III	13	30	i24929Gh-i13848Gh	13	40	i46668Gh-i00187Gh	10.07	2.68	1.97	0.71
	B_1meq-FE-9	III	18	55	i13319Gh-i42821Gh	24	20	i36485Gh-i41754Gh	9.71	3.07	2.19	0.88
	B_1meq-FE-10	III	19	20	i08987Gh-i09220Gh	25	30	i11464Gh-i46788Gh	9.43	2.53	1.89	0.64
	B_1meq-FE-11	III	20	60	i11915Gh-i11478Gh	21	60	i22642Gh-i41613Gh	10.62	3.80	2.81	0.99
	B_1meq-FE-12	III	24	40	i48423Gh-i43942Gh	26	20	i37251Gh-i23249Gh	10.20	3.25	2.42	0.83
	B_1meq-FE-13	II	24	55	i14999Gh-i14993Gh	25	40	**i22495Gh-i55440Gb**	9.40	3.20	2.40	0.80
FS	B_1meq-FS-1	III	4	5	i26515Gh-i43091Gh	22	40	i12810Gh-i17697Gh	9.16	2.85	2.11	0.74
	B_1meq-FS-2	III	5	20	i35761Gh-i09052Gh	11	40	i43181Gh-i16165Gh	9.44	3.35	2.54	0.82
	B_1meq-FS-3	III	7	20	i01696Gh-i57601Gb	25	30	i11464Gh-i46788Gh	8.29	2.03	1.51	0.52
	B_1meq-FS-4	III	7	35	i42900Gh-i21721Gh	14	85	i05035Gh-i22015Gh	8.07	2.43	1.81	0.62
	B_1meq-FS-5	III	8	0	i63682Gm-i37825Gh	8	50	i01126Gh-i04719Gh	8.56	3.10	2.33	0.77
	B_1meq-FS-6	III	9	40	i10438Gh-i08573Gh	22	0	i00456Gh-i12928Gh	8.43	2.88	2.11	0.76
	B_1meq-FS-7	III	9	75	i05825Gh-i14639Gh	13	15	i30934Gh-i18151Gh	8.55	2.25	1.70	0.55
	B_1meq-FS-8	III	14	50	i34963Gh-i44045Gh	16	50	i46435Gh-i58367Gb	9.98	2.96	2.16	0.80
	B_1meq-FS-9	III	15	5	i02306Gh-i02317Gh	24	40	i48423Gh-i43942Gh	10.40	3.71	2.78	0.93
	B_1meq-FS-10	III	17	0	i14907Gh-i14878Gh	17	20	i03593Gh-i03537Gh	10.52	3.02	2.27	0.76
	B_1meq-FS-11	III	18	55	i13319Gh-i42821Gh	24	20	i36485Gh-i41754Gh	9.60	3.03	2.17	0.86
	B_1meq-FS-12	III	19	20	i08987Gh-i09220Gh	25	30	i11464Gh-i46788Gh	9.23	2.38	1.79	0.59
	B_1meq-FS-13	III	20	45	i17500Gh-i47439Gh	21	40	i07558Gh-i07515Gh	10.77	2.80	2.09	0.71
	B_1meq-FS-14	III	22	40	i12810Gh-i17697Gh	26	5	i08062Gh-i33827Gh	10.55	3.45	2.58	0.87
	B_1meq-FS-15	III	22	40	i12810Gh-i17697Gh	26	5	i08062Gh-i33827Gh	10.39	3.47	2.61	0.86
	B_1meq-FS-16	III	24	40	i48423Gh-i43942Gh	26	20	i37251Gh-i23249Gh	10.23	3.29	2.44	0.85
	B_1meq-FS-17	III	24	55	i14999Gh-i14993Gh	25	40	i22495Gh-i55440Gb	9.49	3.25	2.43	0.83

					**$MARBCF_1s$**							
FL	B_2meq-FL-1	III	1	25	i14664Gh-i02994Gh	6	55	i06396Gh-i06056Gh	7.57	2.62	2.42	0.21
	B_2meq-FL-2	III	3	45	i35903Gh-i20966Gh	7	5	i37773Gh-i30640Gh	6.75	2.68	2.34	0.34
	B_2meq-FL-3	III	7	15	i46540Gh-i01765Gh	8	55	i01126Gh-i04719Gh	7.75	2.99	2.41	0.57
	B_2meq-FL-4	III	3	40	i00971Gh-i46613Gh	9	65	i49158Gh-i00393Gh	7.01	2.76	2.71	0.05
	B_2meq-FL-5	III	1	10	i53010Gb-i21390Gh	9	75	i05825Gh-i14639Gh	6.16	2.33	1.62	0.71
	B_2meq-FL-6	III	2	55	i02246Gh-i00463Gh	11	0	i52789Gb-i07420Gh	6.75	2.62	2.61	0.01
	B_2meq-FL-7	III	3	35	i27670Gh-i40392Gh	16	55	i21384Gh-i44137Gh	6.83	2.71	2.71	0.00
	B_2meq-FL-8	III	2	95	i07717Gh-i09654Gh	17	25	i03512Gh-i22912Gh	6.10	2.34	2.11	0.23
	B_2meq-FL-9	III	15	25	i18410Gh-i38002Gh	17	40	i03508Gh-i18575Gh	7.32	2.78	2.54	0.23
	B_2meq-FL-10	III	3	35	i27670Gh-i40392Gh	17	55	i03522Gh-i03688Gh	6.35	2.54	2.34	0.20
	B_2meq-FL-11	III	14	50	i34963Gh-i44045Gh	19	5	i16591Gh-i08933Gh	6.33	2.31	2.17	0.14
	B_2meq-FL-12	III	17	55	i03522Gh-i03688Gh	20	0	i17414Gh-i17417Gh	10.10	3.99	3.81	0.18
	B_2meq-FL-13	III	6	35	i06036Gh-i06037Gh	21	65	i22642Gh-i41613Gh	9.90	3.52	3.04	0.49
	B_2meq-FL-14	III	7	55	i01629Gh-i14398Gh	24	20	i36485Gh-i41754Gh	6.76	2.54	2.20	0.34
	B_2meq-FL-15	III	16	60	i27803Gh-i58144Gb	24	20	i36485Gh-i41754Gh	8.04	3.03	2.62	0.40
	B_2meq-FL-16	III	21	60	i22642Gh-i41613Gh	26	0	i00879Gh-i08691Gh	6.14	2.52	2.50	0.03
	B_2meq-FL-17	III	10	60	i00538Gh-i22625Gh	26	25	i08562Gh-i49188Gh	6.23	2.09	1.70	0.39
FU	B_2meq-FU-1	III	7	60	i14398Gh-i01824Gh	19	0	i09067Gh-i09082Gh	6.84	2.72	1.11	1.61
	B_2meq-FU-2	II	24	45	**i26213Gh-i00339Gh**	25	50	i40453Gh-i46187Gh	6.19	2.47	0.38	2.09
MIC	B_2meq-MIC-1	III	1	25	i14664Gh-i02994Gh	6	5	i30129Gh-i06111Gh	6.92	2.89	2.85	0.04
	B_2meq-MIC-2	III	3	70	i22169Gh-i33635Gh	11	20	i36064Gh-i44818Gh	6.36	3.14	2.44	0.70
	B_2meq-MIC-3	II	14	10	**i46775Gh-i43468Gh**	14	30	i35260Gh-i35101Gh	6.21	1.56	1.56	0.00
	B_2meq-MIC-4	III	11	20	i36064Gh-i44818Gh	15	15	i02469Gh-i46030Gh	6.09	2.47	1.54	0.92
	B_2meq-MIC-5	III	9	0	i40221Gh-i15598Gh	17	30	i14920Gh-i51624Gb	7.64	2.36	1.69	0.67
	B_2meq-MIC-6	III	7	25	i33174Gh-i01631Gh	19	35	i50235Gb-i52709Gb	7.48	2.82	2.64	0.18

	B_2meq-MIC-7	III	14	30	i35260Gh-i35101Gh	20	5	i00478Gh-i11539Gh	6.03	2.43	1.50	0.93
	B_2meq-MIC-8	III	9	80	i47965Gh-i03487Gh	21	25	i22367Gh-i47711Gh	6.08	3.03	2.46	0.57
	B_2meq-MIC-9	III	6	10	i06061Gh-i05824Gh	21	70	i33529Gh-i43642Gh	6.70	3.57	2.62	0.95
	B_2meq-MIC-10	III	6	20	i22608Gh-i06067Gh	25	25	i27022Gh-i11449Gh	6.68	3.39	2.56	0.83
	B_2meq-MIC-11	III	14	60	i26838Gh-i01129Gh	26	40	i36067Gh-i08578Gh	6.20	2.08	2.03	0.05
FE	B_2meq-FE-1	III	8	0	i63682Gm-i37825Gh	13	40	i46668Gh-i00187Gh	6.35	4.42	2.23	2.19

[a] FL: fiber length; FU: fiber uniformity; MIC: micronaire; FE: fiber elongation; FS: fiber strength

[b] Type of epistasis: (I) two loci with m-QTL, (II) one loci with m-QTL and the other loci without significant m-QTL and (III) two loci without significant m-QTL

[c] Chi and Chj represent the chromosome number of the loci being tested in the analysis

[d] Position of e-QTL located on chromosome: as cM distance from the top of each chromosome

[e] Flanking markers in bold are those flanking m-QTLs identified by ICIM in additional Table S7

[f] A LOD threshold was used for declaration of QTL based on 1000 permutations at as significance level of 0.01

[g] PV: the phenotypic variation that the total epistasis effect explained; PV(AA): the phenotypic variation that the main epistasis effect explained; PV (AAE): the phenotypic variation that the environmental interaction of epistasis effect explained
