# Supplementary material for: QTL Mapping and Heterosis Analysis for Fiber Quality Traits Across Multiple Genetic Populations and Environments in Upland Cotton
Source: Front Plant Sci. 2018 Oct 15;9:1364. doi: 10.3389/fpls.2018.01364 (PMC6196769; doi:10.3389/fpls.2018.01364)
Supplement: Supplementary file 10 [file Data_Sheet_10.PDF]

Table S10 Epistatic effects and environmental interactions detected for fiber quality traits in IF_2MPH, $HSBCF_1MPH$, and $MARBCF_1MPH$ datasets by ICIM method

Traits[a]	e-QTL	Type of epistasis[b]	Chr.i[c]	Position1[d]	Flanking markers 1[e]	Chr.j[c]	Position2[d]	Flanking markers 2[e]	LOD[f]	PV[g]	PV(AA)[g]	PV(AAE)[g]
						IF_2MPHs						
FL	IMmeq-FL-1	III	4	20	i00135Gh-i47058Gh	5	30	i53001Gb-i08984Gh	8.0367	4.3004	2.7548	1.5455
	IMmeq-FL-2	III	5	10	i38565Gh-i36865Gh	5	30	i53001Gb-i08984Gh	8.4539	4.6559	2.5539	2.102
	IMmeq-FL-3	III	5	30	i53001Gb-i08984Gh	8	5	i31145Gh-i40270Gh	9.086	5.1156	2.1151	3.0006
	IMmeq-FL-4	III	5	30	i53001Gb-i08984Gh	9	45	i46552Gh-i24387Gh	8.413	4.4599	2.1927	2.2671
	IMmeq-FL-5	III	5	30	i53001Gb-i08984Gh	11	35	i40251Gh-i07190Gh	9.608	5.2064	2.7167	2.4897
	IMmeq-FL-6	III	7	10	i24917Gh-i26814Gh	13	35	i37089Gh-i41278Gh	9.9599	4.8661	2.0843	2.7819
	IMmeq-FL-7	III	9	15	i40336Gh-i07864Gh	15	30	i02459Gh-i02486Gh	8.2177	4.2355	3.6856	0.5499
	IMmeq-FL-8	III	5	30	i53001Gb-i08984Gh	15	40	i02315Gh-i07215Gh	8.6124	4.2597	2.2591	2.0006
	IMmeq-FL-9	III	10	15	i25267Gh-i30274Gh	18	15	i31442Gh-i13146Gh	8.1758	4.2546	0.5408	3.7138
	IMmeq-FL-10	III	4	0	i50068Gb-i26515Gh	20	40	i37554Gh-i47006Gh	8.1359	3.7949	2.3398	1.4551
	IMmeq-FL-11	III	18	15	i31442Gh-i13146Gh	20	40	i37554Gh-i47006Gh	8.9896	4.3877	2.211	2.1767
	IMmeq-FL-12	III	21	25	i22367Gh-i47711Gh	21	50	i35971Gh-i47631Gh	8.1939	2.9013	2.6074	0.2939
	IMmeq-FL-13	III	5	30	i53001Gb-i08984Gh	22	25	i12539Gh-i12581Gh	8.603	3.8589	2.1485	1.7104
	IMmeq-FL-14	III	21	60	i22642Gh-i41613Gh	22	45	i44682Gh-i25111Gh	8.5442	3.7811	1.8219	1.9592
	IMmeq-FL-15	III	8	20	i25482Gh-i25868Gh	24	55	i14999Gh-i14993Gh	8.0507	4.1593	1.8865	2.2728
	IMmeq-FL-16	III	21	50	i35971Gh-i47631Gh	25	50	i40453Gh-i46187Gh	8.9778	4.292	1.0059	3.2861
	IMmeq-FL-17	III	5	30	i53001Gb-i08984Gh	25	55	i11287Gh-i17145Gh	8.579	3.7994	1.3904	2.409
	IMmeq-FL-18	III	14	85	i05035Gh-i22015Gh	26	10	i33827Gh-i25834Gh	11.3347	5.9649	4.1147	1.8502
	IMmeq-FL-19	III	20	5	i00478Gh-i11539Gh	26	10	i33827Gh-i25834Gh	9.0765	4.2962	3.041	1.2552
FU	IMmeq-FU-1	III	7	65	i14398Gh-i01824Gh	9	80	i47965Gh-i03487Gh	8.3777	3.3123	0.4973	2.815
	IMmeq-FU-2	III	5	40	i08988Gh-i45534Gh	12	0	i40974Gh-i48211Gh	12.5721	2.2914	0.3011	1.9903
	IMmeq-FU-3	III	9	5	i25689Gh-i17373Gh	14	25	i15375Gh-i05040Gh	8.3126	3.5892	0.0668	3.5223
	IMmeq-FU-4	III	9	75	i05825Gh-i14639Gh	15	0	i02955Gh-i02314Gh	8.5626	3.9913	0.7365	3.2548

	IMmeq-FU-5	III	12	0	i40974Gh-i48211Gh	19	20	i08987Gh-i09220Gh	8.405	2.1434	0.4457	1.6977
	IMmeq-FU-6	III	5	65	i37142Gh-i48326Gh	20	5	i00478Gh-i11539Gh	8.153	3.9091	0.8117	3.0974
	IMmeq-FU-7	III	11	0	i52789Gb-i07420Gh	20	5	i00478Gh-i11539Gh	9.0226	1.8465	1.0817	0.7647
	IMmeq-FU-8	III	8	20	i25482Gh-i25868Gh	22	50	i44682Gh-i25111Gh	8.4857	3.3596	0.27	3.0896
	IMmeq-FU-9	III	20	40	i37554Gh-i47006Gh	24	10	i04567Gh-i15176Gh	8.4672	4.2347	0.4207	3.8139
	IMmeq-FU-10	III	23	0	i06287Gh-i06171Gh	25	20	i41210Gh-i42629Gh	8.1712	4.6046	1.1626	3.442
	IMmeq-FU-11	III	14	25	i15375Gh-i05040Gh	25	25	i27022Gh-i11449Gh	9.6489	4.4223	0.9671	3.4552
	IMmeq-FU-12	III	20	40	i37554Gh-i47006Gh	26	10	i33827Gh-i25834Gh	8.6265	3.5233	0.4931	3.0302
MIC	IMmeq-MIC-1	III	3	95	i42939Gh-i05394Gh	5	40	i08988Gh-i45534Gh	13.0563	2.0999	1.415	0.6849
	IMmeq-MIC-2	III	21	10	i06952Gh-i07714Gh	21	45	i07446Gh-i16079Gh	8.9765	0.1563	0.1432	0.0132
FE	IMmeq-FE-1	III	1	35	i02298Gh-i42430Gh	1	50	i31438Gh-i26341Gh	12.175	0.0188	1.798	0
	IMmeq-FE-2	III	4	30	i10502Gh-i36496Gh	7	45	i35445Gh-i13943Gh	9.571	0.0257	0.1682	1.2248
	IMmeq-FE-3	III	4	5	i26515Gh-i43091Gh	8	15	i04570Gh-i04506Gh	13.4406	0.0361	0.0074	0.0556
	IMmeq-FE-4	III	1	50	i31438Gh-i26341Gh	10	65	i22625Gh-i22107Gh	12.9022	0.0629	0.0636	0.7096
	IMmeq-FE-5	III	11	15	i01036Gh-i07468Gh	11	25	i07163Gh-i56975Gb	8.7282	0.0753	0.0589	0.3197
	IMmeq-FE-6	III	3	35	i27670Gh-i40392Gh	14	30	i35260Gh-i35101Gh	8.8051	0.0815	0.0897	0.298
	IMmeq-FE-7	III	11	25	i07163Gh-i56975Gb	14	65	i05772Gh-i05775Gh	12.4416	0.1288	0.0207	0.159
	IMmeq-FE-8	III	10	65	i22625Gh-i22107Gh	14	70	i38712Gh-i23682Gh	12.311	0.1734	0.2302	0.8458
	IMmeq-FE-9	III	9	10	i05758Gh-i19700Gh	16	5	i31225Gh-i35502Gh	14.3055	0.1797	0.1259	0.1789
	IMmeq-FE-10	III	3	5	i34758Gh-i49377Gh	17	5	i03245Gh-i46085Gh	9.379	0.2681	0.1288	0
	IMmeq-FE-11	III	9	10	i05758Gh-i19700Gh	17	25	i03512Gh-i22912Gh	8.9617	0.3048	0.3886	1.1576
	IMmeq-FE-12	III	11	25	i07163Gh-i56975Gb	17	40	i03508Gh-i18575Gh	12.7024	0.3737	2.5308	4.7974
	IMmeq-FE-13	III	18	55	i13319Gh-i42821Gh	19	35	i50235Gb-i52709Gb	10.7117	0.3786	0.0319	0.1415
	IMmeq-FE-14	III	13	55	i21560Gh-i46408Gh	20	55	i12251Gh-i11715Gh	8.7963	0.3878	0.8046	1.1141
	IMmeq-FE-15	III	4	0	i50068Gb-i26515Gh	21	45	i07446Gh-i16079Gh	10.7733	0.4359	0.1416	0.2943
	IMmeq-FE-16	III	12	50	i08275Gh-i16368Gh	22	15	i43141Gh-i60448Gb	12.778	0.7732	0.0361	0

	IMmeq-FE-17	III	18	50	i13451Gh-i38577Gh	22	15	i43141Gh-i60448Gb	11.7239	1.076	0.3643	0.0094
	IMmeq-FE-18	III	4	5	i26515Gh-i43091Gh	23	25	i06171Gh-i06175Gh	11.9549	1.0817	0.0058	0.013
	IMmeq-FE-19	III	12	50	i08275Gh-i16368Gh	23	30	i26750Gh-i31332Gh	12.8861	1.393	0.0753	0
	IMmeq-FE-20	III	20	55	i12251Gh-i11715Gh	24	50	i38401Gh-i04575Gh	9.9101	1.5461	0.2882	0.7935
	IMmeq-FE-21	III	23	30	i26750Gh-i31332Gh	24	50	i38401Gh-i04575Gh	12.8461	1.798	0.0051	0.0206
	IMmeq-FE-22	III	4	30	i10502Gh-i36496Gh	24	65	i03764Gh-i03721Gh	8.3015	1.9187	0.2538	0.0144
	IMmeq-FE-23	III	15	5	i02306Gh-i02317Gh	25	25	i27022Gh-i11449Gh	8.477	4.6756	2.1374	2.5381
	IMmeq-FE-24	III	1	0	i33646Gh-i40884Gh	26	15	i25512Gh-i07941Gh	8.7028	5.4793	1.7728	3.7065
	IMmeq-FE-25	III	16	45	i54957Gb-i59324Gb	26	50	i16464Gh-i28856Gh	9.9639	7.3282	0.0081	0.0735
FS	IMmeq-FS-1	III	2	0	i17680Gh-i02755Gh	3	75	i43226Gh-i45963Gh	9.9921	4.7875	2.381	2.4065
	IMmeq-FS-2	III	5	40	i08988Gh-i45534Gh	17	45	i00956Gh-i42547Gh	10.2578	0.5764	0.0871	0.4893
	IMmeq-FS-3	III	4	0	i50068Gb-i26515Gh	18	40	i26970Gh-i39369Gh	8.4703	4.6971	1.9117	2.7854
	IMmeq-FS-4	III	9	45	i46552Gh-i24387Gh	20	10	i39228Gh-i34769Gh	8.7761	4.1116	2.0121	2.0995
**$HSBCF_1MPHs$**												
FL	B_1Mmeq-FL-1	III	2	75	i02276Gh-i43470Gh	14	15	i15343Gh-i31037Gh	8.31	2.9721	2.843	0.129
	B_1Mmeq-FL-2	III	2	85	i38985Gh-i30800Gh	15	5	i02306Gh-i02317Gh	6.1571	2.0034	1.161	0.8424
	B_1Mmeq-FL-3	III	14	10	i46775Gh-i43468Gh	15	35	i17844Gh-i37620Gh	6.7396	2.253	2.0099	0.2431
	B_1Mmeq-FL-4	III	15	25	i18410Gh-i38002Gh	19	5	i16591Gh-i08933Gh	6.0457	2.2133	1.8609	0.3524
	B_1Mmeq-FL-5	III	11	0	i52789Gb-i07420Gh	19	15	i28797Gh-i09073Gh	7.5057	2.7022	2.5407	0.1615
	B_1Mmeq-FL-6	III	6	15	i19214Gh-i31843Gh	20	20	i26356Gh-i11706Gh	6.2072	2.3137	2.2736	0.0401
	B_1Mmeq-FL-7	III	7	10	i24917Gh-i26814Gh	26	5	i08062Gh-i33827Gh	6.0204	2.2817	1.4635	0.8182
FU	B_1Mmeq-FU-1	III	2	70	i00890Gh-i02271Gh	4	30	i10502Gh-i36496Gh	9.4664	3.5265	1.4195	2.1069
	B_1Mmeq-FU-2	III	4	30	i10502Gh-i36496Gh	6	45	i23722Gh-i37862Gh	8.0676	3.4883	0.8042	2.6841
	B_1Mmeq-FU-3	III	7	0	i32739Gh-i37773Gh	7	10	i24917Gh-i26814Gh	10.7565	0.7124	0.7124	0
	B_1Mmeq-FU-4	III	4	30	i10502Gh-i36496Gh	9	5	i25689Gh-i17373Gh	8.2439	2.8229	1.2525	1.5704
	B_1Mmeq-FU-5	III	4	30	i10502Gh-i36496Gh	13	55	i21560Gh-i46408Gh	8.4394	4.6434	1.3016	3.3418

	B_1Mmeq-FU-6	III	4	30	i10502Gh-i36496Gh	14	35	i04933Gh-i43400Gh	12.3256	6.7366	1.9991	4.7375
	B_1Mmeq-FU-7	III	6	45	i23722Gh-i37862Gh	14	35	i04933Gh-i43400Gh	8.5222	2.3481	0.6989	1.6493
	B_1Mmeq-FU-8	III	14	35	i04933Gh-i43400Gh	16	15	i14406Gh-i01766Gh	8.9274	2.8336	1.4495	1.3841
	B_1Mmeq-FU-9	III	4	30	i10502Gh-i36496Gh	16	45	i54957Gb-i59324Gb	8.3538	5.6999	1.5286	4.1712
	B_1Mmeq-FU-10	III	6	45	i23722Gh-i37862Gh	16	50	i46435Gh-i58367Gb	11.8929	3.5126	1.0037	2.5088
	B_1Mmeq-FU-11	III	4	30	i10502Gh-i36496Gh	18	105	i45991Gh-i13081Gh	12.05	4.7116	2.0707	2.6408
	B_1Mmeq-FU-12	III	6	55	i06396Gh-i06056Gh	22	30	i12906Gh-i17853Gh	8.3417	3.9572	1.5389	2.4183
	B_1Mmeq-FU-13	III	13	55	i21560Gh-i46408Gh	22	30	i12906Gh-i17853Gh	8.5081	4.5646	1.5302	3.0345
	B_1Mmeq-FU-14	III	14	35	i04933Gh-i43400Gh	22	30	i12906Gh-i17853Gh	12.0793	7.7982	1.7383	6.0599
	B_1Mmeq-FU-15	III	18	35	i32883Gh-i13851Gh	22	30	i12906Gh-i17853Gh	10.0649	5.361	1.5506	3.8104
	B_1Mmeq-FU-16	III	19	30	i16566Gh-i08941Gh	22	30	i12906Gh-i17853Gh	10.0845	1.9278	0.8276	1.1002
	B_1Mmeq-FU-17	III	4	30	i10502Gh-i36496Gh	24	30	i15200Gh-i04548Gh	10.0671	3.7326	1.4692	2.2634
	B_1Mmeq-FU-18	III	22	30	i12906Gh-i17853Gh	24	35	i04535Gh-i04423Gh	11.801	4.6287	1.3449	3.2837
MIC	B_1Mmeq-MIC-1	III	5	10	i38565Gh-i36865Gh	8	30	i54149Gb-i00217Gh	6.5679	1.9684	1.6094	0.359
	B_1Mmeq-MIC-2	III	10	50	i33011Gh-i22938Gh	10	65	i22625Gh-i22107Gh	6.4213	1.3198	1.3198	0
	B_1Mmeq-MIC-3	III	14	10	i46775Gh-i43468Gh	25	5	i00735Gh-i41210Gh	7.7078	1.5874	1.1696	0.4178
	B_1Mmeq-MIC-4	III	16	35	i01640Gh-i00384Gh	25	5	i00735Gh-i41210Gh	6.6601	2.7659	1.7476	1.0182
	B_1Mmeq-MIC-5	III	21	40	i07558Gh-i07515Gh	25	5	i00735Gh-i41210Gh	6.7951	0.8517	0.7917	0.06
	B_1Mmeq-MIC-6	III	25	5	i00735Gh-i41210Gh	25	60	i17145Gh-i10628Gh	6.2774	1.5845	1.1465	0.438
	B_1Mmeq-MIC-7	III	25	5	i00735Gh-i41210Gh	26	40	i36067Gh-i08578Gh	7.5683	0.9544	0.9544	0
FE	B_1Mmeq-FE-1	III	1	25	i14664Gh-i02994Gh	3	5	i34758Gh-i49377Gh	6.3922	3.0464	1.4127	1.6337
	B_1Mmeq-FE-2	III	3	5	i34758Gh-i49377Gh	18	50	i13451Gh-i38577Gh	6.2784	2.52	1.1401	1.3799
	B_1Mmeq-FE-3	III	22	20	i12927Gh-i12929Gh	22	30	i12906Gh-i17853Gh	6.2861	0.6442	0.6442	0
	B_1Mmeq-FE-4	III	3	30	i00583Gh-i27670Gh	23	0	i06287Gh-i06171Gh	6.6031	1.6711	0.9352	0.7359
FS	B_1Mmeq-FS-1	III	3	65	i49177Gh-i39896Gh	10	60	i00538Gh-i22625Gh	6.8075	2.6816	2.5122	0.1694
	B_1Mmeq-FS-2	III	8	20	i25482Gh-i25868Gh	13	40	i46668Gh-i00187Gh	6.8041	2.8153	2.492	0.3233

**MARBCF$_1$MPHs**												
FL	B_2Mmeq-FL-1	III	5	65	i37142Gh-i48326Gh	7	10	i24917Gh-i26814Gh	6.8701	2.8336	1.9991	0.8345
	B_2Mmeq-FL-2	III	7	10	i24917Gh-i26814Gh	9	0	i40221Gh-i15598Gh	6.127	2.7028	2.2783	0.4245
	B_2Mmeq-FL-3	III	4	30	i10502Gh-i36496Gh	9	40	i10438Gh-i08573Gh	6.9193	2.8432	2.6556	0.1876
	B_2Mmeq-FL-4	III	8	45	i04524Gh-i01126Gh	9	90	i13502Gh-i25039Gh	6.2454	3.0788	2.7213	0.3575
	B_2Mmeq-FL-5	III	14	45	i27231Gh-i36385Gh	14	55	i23629Gh-i15587Gh	6.368	2.8174	2.2717	0.5457
	B_2Mmeq-FL-6	III	13	40	i46668Gh-i00187Gh	15	15	i02469Gh-i46030Gh	6.5099	2.8127	2.5276	0.285
	B_2Mmeq-FL-7	III	4	30	i10502Gh-i36496Gh	17	35	i03218Gh-i03216Gh	6.7952	3.0926	2.8755	0.2171
	B_2Mmeq-FL-8	III	3	10	i32361Gh-i00583Gh	21	65	i22642Gh-i41613Gh	6.3459	2.9229	2.5671	0.3558
	B_2Mmeq-FL-9	III	2	25	i02761Gh-i02712Gh	24	50	i38401Gh-i04575Gh	6.7864	2.976	2.1636	0.8124
	B_2Mmeq-FL-10	III	5	40	i08988Gh-i45534Gh	24	50	i38401Gh-i04575Gh	6.2768	2.4087	1.631	0.7776
	B_2Mmeq-FL-11	III	16	45	i54957Gb-i59324Gb	24	50	i38401Gh-i04575Gh	6.4606	2.8911	2.669	0.2221
	B_2Mmeq-FL-12	III	10	60	i00538Gh-i22625Gh	26	25	i08562Gh-i49188Gh	6.2567	2.2239	1.6768	0.5471
FU	B_2Mmeq-FU-1	III	24	45	i26213Gh-i00339Gh	25	50	i40453Gh-i46187Gh	6.4138	2.613	0.8112	1.8018
MIC	B_2Mmeq-MIC-1	III	1	15	i02201Gh-i27043Gh	9	10	i05758Gh-i19700Gh	7.0587	2.2812	1.3183	0.9629
	B_2Mmeq-MIC-2	III	3	65	i49177Gh-i39896Gh	11	20	i36064Gh-i44818Gh	8.4532	3.7858	2.9575	0.8283
	B_2Mmeq-MIC-3	III	9	0	i40221Gh-i15598Gh	17	30	i14920Gh-i51624Gb	6.3298	1.8615	1.1828	0.6787
	B_2Mmeq-MIC-4	III	2	90	i07717Gh-i09654Gh	19	5	i16591Gh-i08933Gh	6.3251	2.5429	2.2878	0.2551
	B_2Mmeq-MIC-5	III	8	10	i24295Gh-i00234Gh	19	5	i16591Gh-i08933Gh	6.3496	2.6471	1.8656	0.7816
	B_2Mmeq-MIC-6	III	18	5	i13754Gh-i13145Gh	19	5	i16591Gh-i08933Gh	6.0061	2.1131	1.9654	0.1477
	B_2Mmeq-MIC-7	III	5	30	i53001Gb-i08984Gh	20	0	i17414Gh-i17417Gh	6.597	3.3202	2.3518	0.9685
	B_2Mmeq-MIC-8	III	14	15	i15343Gh-i31037Gh	20	35	i40942Gh-i35292Gh	6.8981	3.2302	1.0296	2.2007
	B_2Mmeq-MIC-9	III	19	0	i09067Gh-i09082Gh	21	30	i47711Gh-i07558Gh	6.544	2.072	1.8443	0.2277
	B_2Mmeq-MIC-10	III	14	30	i35260Gh-i35101Gh	25	10	i00735Gh-i41210Gh	6.9535	2.7234	0.7719	1.9514
	B_2Mmeq-MIC-11	III	17	40	i03508Gh-i18575Gh	25	10	i00735Gh-i41210Gh	6.0813	2.7761	2.5151	0.2609
	B_2Mmeq-MIC-12	III	3	60	i35903Gh-i20966Gh	26	15	i25512Gh-i07941Gh	6.3804	3.344	2.7829	0.5611

FE	B_2Mmeq-FE-1	III	2	75	i02276Gh-i43470Gh	14	60	i26838Gh-i01129Gh	8.2751	3.3932	1.5325	1.8606
	B_2Mmeq-FE-2	III	5	30	i53001Gb-i08984Gh	14	60	i26838Gh-i01129Gh	6.7615	3.3714	1.4121	1.9593
	B_2Mmeq-FE-3	III	12	15	i40974Gh-i48211Gh	14	60	i26838Gh-i01129Gh	7.9597	3.2229	1.3061	1.9168
	B_2Mmeq-FE-4	III	10	50	i33011Gh-i22938Gh	15	5	i02306Gh-i02317Gh	7.0991	3.1367	1.1673	1.9694
	B_2Mmeq-FE-5	III	5	40	i08988Gh-i45534Gh	24	50	i38401Gh-i04575Gh	6.0952	3.3757	1.1204	2.2554
FS	B_2Mmeq-FS-1	III	8	30	i54149Gb-i00217Gh	10	0	i43940Gh-i25267Gh	6.0881	2.3392	0.4182	1.921

[a] FL: fiber length; FU: fiber uniformity; MIC: micronaire; FE: fiber elongation; FS: fiber strength

[b] Type of epistasis: (I) two loci with m-QTL, (II) one loci with m-QTL and the other loci without significant m-QTL and (III) two loci without significant m-QTL

[c] Chi and Chj represent the chromosome number of the loci being tested in the analysis

[d] Position of e-QTL located on chromosome: as cM distance from the top of each chromosome

[e] Flanking markers in bold are those flanking m-QTLs identified by ICIM in additional Table S8

[f] A LOD threshold was used for declaration of QTL based on 1000 permutations at as significance level of 0.01

[g] PV: the phenotypic variation that the total epistasis effect explained; PV(AA): the phenotypic variation that the main epistasis effect explained; PV (AAE): the phenotypic variation that the environmental interaction of epistasis effect explained
