# Supplementary material for: QTL Mapping and Heterosis Analysis for Fiber Quality Traits Across Multiple Genetic Populations and Environments in Upland Cotton
Source: Front Plant Sci. 2018 Oct 15;9:1364. doi: 10.3389/fpls.2018.01364 (PMC6196769; doi:10.3389/fpls.2018.01364)
Supplement: Supplementary file 11 [file Data_Sheet_11.PDF]

Table S11 HLs identified for fiber quality traits in IF_2MPH, $HSBCF_1MPH$, and $MARBCF_1MPH$ datasets by CIM method

Trait[a]	HL[b]	Env.[c]	Flanking markers	Position[d]	LOD[e]	A[f]	D[f]	A+D[f]	R²（%）[g]	Population
FL	qFL-C02-1	2014Bg	i43421Gh-i24299Gh	41.21	5		-2.15		19.31	$MARBCF_1MPH$
		2015Bg	i14776Gh-i16398Gh	43.91	2.59		-2.19		12.2	IF_2MPH
	qFL-C02-2	2015Bg	i02276Gh-i01044Gh	75.71	2.8		0.56		6.28	IF_2MPH
		2015Yc	i49488Gh-i14841Gh	78.11	3.03		0.18		9.51	$HSBCF_1MPH$
	**qFL-C05-3**	2014Yc	i16666Gh-i51323Gb	50.81	3.37		0.76		13.32	$HSBCF_1MPH$
	qFL-C06-1	2015Bg	i21566Gh-i14061Gh	25.61	2.65		0.49		14.64	IF_2MPH
		2014Bg	i06526Gh-i34827Gh	27.81	2.93		-2.47		10.6	$MARBCF_1MPH$
	qFL-C14-1	2014Bg	i05482Gh-i18840Gh	4.01	4.21		-2.61		19.34	$MARBCF_1MPH$
	**qFL-C14-3**	2015Yc	i15343Gh-i15345Gh	18.41	2.69		-0.3		7.93	$MARBCF_1MPH$
	qFL-C16-2	2015Bg	i21384Gh-i42534Gh	57.21	4.2		0.79		6.93	IF_2MPH
	qFL-C18-1	2014Bg	i31525Gh-i26380Gh	58.61	4.72		-2.3		20.92	$MARBCF_1MPH$
		2014Bg	i48138Gh-i13492Gh	59.01	3.25		-2.68		10.35	$HSBCF_1MPH$
	qFL-C19-1	2014Yc	i28797Gh-i50906Gb	16.01	4.91		0.8		10.36	$HSBCF_1MPH$
	qFL-C20-2	2014Bg	i11727Gh-i39228Gh	8.01	2.76		-0.61		4.99	IF_2MPH
	qFL-C21-1	2014Bg	i16082Gh-i00284Gh	44.61	3.26		-0.76		3.82	$HSBCF_1MPH$
	qFL-C22-1	2014Yc	i00125Gh-i30763Gh	7.51	2.52		0.86		14.63	$MARBCF_1MPH$
	qFL-C22-2	2014Bg	i22757Gh-i12927Gh	16.71	5		-2.24		13.08	$MARBCF_1MPH$

	qFL-C22-3	2014Bg	i12927Gh-i33209Gh	20.11	2.78	0.37	7.12	IF_2MPH
	qFL-C22-4	2014Bg	i12906Gh-i12725Gh	30.21	2.94	0.39	6.56	IF_2MPH
	qFL-C24-1	2015Bg	i04105Gh-i18808Gh	5.41	4.86	-1.27	14.48	$MARBCF_1MPH$
FU	qFU-C01-2	2015Bg	i02245Gh-i02457Gh	45.71	6.83	-7.4	15.2	$HSBCF_1MPH$
	qFU-C02-1	2015Bg	i02758Gh-i02723Gh	44.81	3.75	-6.04	8.84	$HSBCF_1MPH$
	qFU-C03-1	2015Bg	i35903Gh-i39896Gh	52.51	3.17	0.48	14.96	IF_2MPH
	qFU-C03-2	2014Bg	i39896Gh-i33635Gh	69.11	3.11	-0.66	6.05	IF_2MPH
	**qFU-C05-2**	2015Bg	i19536Gh-i34270Gh	46.91	2.72	0.64	5.57	IF_2MPH
	qFU-C06-2	2015Yc	i06037Gh-i06505Gh	36.01	3.91	-0.9	3.89	$HSBCF_1MPH$
		2015Yc	i06037Gh-i23722Gh	36.01	3.13	1.12	3.7	IF_2MPH
	**qFU-C09-1**	2014Bg	i07864Gh-i50078Gb	17.81	93.26	27.7	17.47	$HSBCF_1MPH$
	qFU-C11-1	2015Bg	i33855Gh-i43823Gh	5.31	3.69	3.44	10.43	IF_2MPH
	qFU-C14-1	2015Bg	i15536Gh-i05487Gh	1.11	3.74	4.14	11.1	IF_2MPH
	qFU-C16-1	2015Bg	i54704Gb-i01693Gh	72.91	9.97	4.93	19.23	$HSBCF_1MPH$
	qFU-C20-2	2015Bg	i11714Gh-i37554Gh	38.01	11.31	4.69	11.01	$HSBCF_1MPH$
	qFU-C20-3	2015Yc	i17505Gh-i47439Gh	49.11	3.14	-0.7	11.41	$HSBCF_1MPH$
	qFU-C20-4	2015Bg	i18012Gh-i11478Gh	57.81	3.68	-5.39	10.76	$HSBCF_1MPH$
	qFU-C21-1	2015Bg	i16082Gh-i00284Gh	44.61	2.86	-1.43	13.42	$MARBCF_1MPH$

		2015Yc	i16082Gh-i00284Gh	44.61	2.69	0.01	6.98	IF_2MPH
	qFU-C21-2	2015Yc	i41432Gh-i22642Gh	58.91	3.07	-0.1	7.32	IF_2MPH
		2015Yc	i07219Gh-i41613Gh	59.91	3.1	0.63	3.58	$MARBCF_1MPH$
	qFU-C23-1	2015Bg	i06287Gh-i06171Gh	0.01	3.26	-1.32	5.23	$MARBCF_1MPH$
	qFU-C26-1	2015Bg	i00879Gh-i32452Gh	1.01	3.75	-0.59	10.03	$MARBCF_1MPH$
		2015Yc	i00879Gh-i33827Gh	2.51	3.32	0.39	10.82	$HSBCF_1MPH$
MIC	qMIC-C01-3	2015Yc	i30614Gh-i02994Gh	24.91	4.54	0.57	3.6	IF_2MPH
	qMIC-C01-4	2015Yc	i02245Gh-i44115Gh	45.71	3.74	-0.32	3.88	IF_2MPH
	qMIC-C02-1	2015Yc	i18644Gh-i27649Gh	35.81	4.17	0.33	4.35	$MARBCF_1MPH$
	qMIC-C05-2	2014Bg	i52021Gb-i09236Gh	60.51	2.73	0.03	4.97	$MARBCF_1MPH$
	qMIC-C08-1	2015Yc	i30195Gh-i04557Gh	35.61	3.02	-0.7	5.78	IF_2MPH
		2015Bg	i30195Gh-i04565Gh	35.91	3.11	0.68	5.88	$HSBCF_1MPH$
		2014Yc	i40070Gh-i01126Gh	38.01	2.91	-0.58	5.89	IF_2MPH
	qMIC-C11-1	2015Yc	i07468Gh-i36064Gh	18.11	3.96	0.01	6.29	$HSBCF_1MPH$
	qMIC-C15-1	2015Bg	i29719Gh-i49465Gh	20.51	5.05	0.07	9.85	$HSBCF_1MPH$
		2015Yc	i29719Gh-i49465Gh	20.51	3.3	0.07	10.16	$HSBCF_1MPH$
	qMIC-C15-2	2015Yc	i02486Gh-i22421Gh	32.81	3.42	0.53	10.58	IF_2MPH
	qMIC-C19-2	2014Yc	i09400Gh-i09637Gh	35.81	4.83	-0.15	17.42	$MARBCF_1MPH$
	qMIC-C24-2	2014Bg	i15200Gh-i28524Gh	31.01	2.55	0.11	18.8	IF_2MPH

FE	qFE-C01-2	2015Bg	i02201Gh-i32863Gh	15.91	6.42	0.19	4.3	$HSBCF_1MPH$
		2014Yc	i23944Gh-i39024Gh	17.41	3.65	-0.7	24.18	IF_2MPH
	qFE-C01-3	2014Bg	i14664Gh-i25056Gh	25.71	3.58	-0.38	9.94	$HSBCF_1MPH$
	qFE-C02-1	2015Yc	i17680Gh-i02712Gh	23.11	2.71	0.07	5.51	IF_2MPH
		2014Yc	i02712Gh-i20804Gh	27.31	2.86	1.23	4.7	IF_2MPH
	qFE-C09-1	2015Yc	i05712Gh-i48103Gh	20.51	2.53	-0.03	5.36	$MARBCF_1MPH$
	qFE-C09-3	2015Bg	i03687Gh-i02498Gh	50.61	5.88	0.09	3.39	$HSBCF_1MPH$
	qFE-C09-4	2014Yc	i15489Gh-i22655Gh	57.61	2.72	0.47	3.46	$MARBCF_1MPH$
	qFE-C10-1	2015Bg	i12268Gh-i32655Gh	38.01	4.66	0.09	16.24	$MARBCF_1MPH$
	qFE-C14-1	2015Bg	i15284Gh-i48509Gh	5.31	5.28	-0.48	5.13	$MARBCF_1MPH$
	qFE-C14-5	2014Yc	i38481Gh-i27231Gh	44.41	4.01	-0.59	10.92	IF_2MPH
		2015Bg	i38809Gh-i15488Gh	45.01	3.28	-0.11	3.81	$MARBCF_1MPH$
	qFE-C15-1	2015Yc	i21698Gh-i24483Gh	25.31	2.62	0.06	5.17	$MARBCF_1MPH$
	qFE-C21-1	2015Yc	i06952Gh-i07714Gh	10.91	2.8	0.1	17.29	$MARBCF_1MPH$
	qFE-C24-2	2014Bg	i04688Gh-i04069Gh	38.11	4.35	-0.28	8.97	$MARBCF_1MPH$
		2015Bg	i31637Gh-i15169Gh	40.11	4.8	0.15	23.41	$HSBCF_1MPH$
	qFE-C26-1	2015Bg	i28856Gh-i23175Gh	51.31	2.8	-0.35	19.6	$MARBCF_1MPH$
FS	qFS-C03-1	2014Yc	i43226Gh-i21218Gh	78.81	3.95	-1.19	10.32	$MARBCF_1MPH$

qFS-C07-1	2015Bg	i01696Gh-i01453Gh	21.91	2.64	0.06	9.1	MARBCF$_1$MPH
	2015Yc	i01453Gh-i33174Gh	23.21	2.84	1.46	18.41	MARBCF$_1$MPH
qFS-C09-2	2015Yc	i03687Gh-i02498Gh	50.61	3.02	-2.05	17.51	HSBCF$_1$MPH
qFS-C09-3	2015Yc	i22037Gh-i05194Gh	59.01	2.54	0.98	3.7	MARBCF$_1$MPH
qFS-C13-2	2015Yc	i32083Gh-i18150Gh	32.41	3.05	0.61	8.17	MARBCF$_1$MPH
qFS-C13-3	2015Yc	i20297Gh-i37629Gh	39.21	4.88	-2.12	3.32	HSBCF$_1$MPH
qFS-C18-1	2014Yc	i31442Gh-i20346Gh	20.11	3.05	1.87	7.02	MARBCF$_1$MPH
qFS-C24-1	2015Yc	i04718Gh-i33113Gh	59.01	2.57	1.23	3.24	MARBCF$_1$MPH
	2015Bg	i03705Gh-i03832Gh	60.61	3.04	-0.83	5.05	MARBCF$_1$MPH
qFS-C25-1	2014Bg	i30564Gh-i36160Gh	2.81	2.57	0.28	6.55	IF$_2$MPH

[a] FL: fiber length; FU: fiber uniformity; MIC: micronaire; FE: fiber elongation; FS: fiber strength

[b] HLs in bold are those also identified by CIM in RILs in our previous study (Li et al. 2016)

[c] 2014Yc: Yacheng of Hainan Province in 2014; 2014Bg: Baogang of Hainan Province in 2014; 2015Yc: Yacheng of Hainan Province in 2015; 2015Bg: Baogang of Hainan Province in 2015

[d] Position ofHL located on chromosome: as cM distance from the top of each chromosome

[e] A LOD threshold was used for declaration of QTL based on 1000 permutations at as significance level of 0.01

[f] The genetic expectation of a HL effect obtained is the additive effect (A) and dominant effect (D) when estimated from the IF$_2$s, the additive and dominance effects (A+D) from the BCF$_1$s, and the dominance effect (D) from the MPH values

[g] Phenotypic variance explained by HL
