# Supplementary material for: QTL Mapping and Heterosis Analysis for Fiber Quality Traits Across Multiple Genetic Populations and Environments in Upland Cotton
Source: Front Plant Sci. 2018 Oct 15;9:1364. doi: 10.3389/fpls.2018.01364 (PMC6196769; doi:10.3389/fpls.2018.01364)
Supplement: Supplementary file 12 [file Data_Sheet_12.PDF]

Table S12 HL Clusters/Hotspots for fiber quality

Cluster/Hotspot[a]	Location[b]	HL	Mapping method
Chr01-cluster-1	15-26 cM	qFE-C01-2, qMIC-C01-3, qFE-C01-3	CIM
Chr02-cluster-1	24-44 cM	qFE-C02-1, qMIC-C02-1, qFL-C02-1	CIM
Chr05- cluster-1	46-61 cM	qFU-C05-2, qFU-C05-3, qMIC-C05-2	CIM
Chr09-cluster-1	22 - 38 cM	B_2MmaqFU-C09-1, B_2MmaqFE-C09-1, B_1MmaqFL-C09-1	ICIM
Chr09- cluster-2	50 - 60 cM	qFE-C09-3, qFS- C09-2, qFE-C09-4, qFS- C09-3	CIM
Chr14-cluster-1	1-19 cM	qFU-C14-1, qFE-C14-1, qFL-C14-3	CIM
Chr15-cluster-1	20-33 cM	qMIC-C15-1, qFE-C15-1, qMIC-C15-2	CIM
Chr20-hotspot-1	38-58 cM	qFU-C20-2, qFU-C20-3, qFU-C20-4	CIM
Chr21- cluster-1	44-60 cM	qFL-C21-1, qFU-C21-1, qFU-C21-2	CIM
Chr22-hotspot-1	20-28 cM	qFL-C22-1, qFL-C22-2, qFL-C22-3, qFL-C22-4	CIM

[a] Cluster, a number of HLs for different traits within approximately 20cM regions; Hotspot, a multiple HLs for the same traits within a 20 cM region

[b] Position of HL Cluster/Hotspot located on chromosome: as cM distance from the top of each chromosome
